# Femicide, intimate partner femicide, and non-intimate partner femicide in South Africa: An analysis of 3 national surveys, 1999–2017

**DOI:** 10.1371/journal.pmed.1004330

**Published:** 2024-01-18

**Authors:** Naeemah Abrahams, Shibe Mhlongo, Esnat Chirwa, Bianca Dekel, Asiphe Ketelo, Carl Lombard, Nwabisa Shai, Leane Ramsoomar, Shanaaz Mathews, Gérard Labuschagne, Richard Matzopoulos, Megan Prinsloo, Lorna J. Martin, Rachel Jewkes

**Affiliations:** 1 Gender & Health Research Unit, South African Medical Research Council, Cape Town, South Africa; 2 School of Public Health and Family Medicine: Faculty of Health Sciences, University of Cape Town, Cape Town, South Africa; 3 School of Public Health, University of Witwatersrand, Johannesburg, South Africa; 4 Office of the Executive Scientist, South African Medical Research Council, Pretoria, South Africa; 5 Biostatistics Unit, South African Medical Research Council, Cape Town, South Africa; 6 Division of Epidemiology and Biostatistics, Department of Global Health, Faculty of Medicine and Health Sciences, Stellenbosch University, Cape Town, South Africa; 7 Children’s Institute, University of Cape Town, Cape Town, South Africa; 8 Department Forensic Medicine & Pathology, University of the Witwatersrand, Johannesburg, South Africa; 9 Burden of Disease Research Unit, South African Medical Research Council, Cape Town, South Africa; 10 Forensic Medicine & Toxicology, Department of Pathology, University of Cape Town, Cape Town, South Africa; Addis Ababa University / King’s College London, ETHIOPIA

## Abstract

**Background:**

In most countries, reliable national statistics on femicide, intimate partner femicide (IPF), and non-intimate partner femicide (NIPF) are not available. Surveys are required to collect robust data on this most extreme consequence of intimate partner violence (IPV). We analysed 3 national surveys to compare femicide, IPF, and NIPF from 1999 to 2017 using age-standardised rates (ASRs) and incidence rate ratios (IRRs).

**Methods and findings:**

We conducted 3 national mortuary-based retrospective surveys using weighted cluster designs from proportionate random samples of medicolegal laboratories. We included females 14 years and older who were identified as having been murdered in South Africa in 1999 (*n* = 3,793), 2009 (*n* = 2,363), and 2017 (*n* = 2,407). Further information on the murdered cases were collected from crime dockets during interviews with police investigating officers. Our findings show that South Africa had an IPF rate of 4.9/100,000 female population in 2017. All forms of femicide among women 14 years and older declined from 1999 to 2017. For IPF, the ASR was 9.5/100,000 in 1999. Between 1999 and 2009, the decline for NIPF was greater than for IPF (IRR for NIPF 0.47 (95% confidence interval (CI) 0.42 to 0.53) compared to IRR for IPF 0.69 (95% CI 0.63 to 0.77). Rates declined from 2009 to 2017 and did not differ by femicide type. The decline in IPF was initially larger for women aged 14 to 29, and after 2009, it was more pronounced for those aged 30 to 44 years. Study limitations include missing data from the police and having to use imputation to account for missing perpetrator data.

**Conclusions:**

In this study, we observed a reduction in femicide overall and different patterns of change in IPF compared to NIPF. The explanation for the reductions may be due to social and policy interventions aimed at reducing IPV overall, coupled with increased social and economic stability. Our study shows that gender-based violence is preventable even in high-prevalence settings, and evidence-based prevention efforts must be intensified globally. We also show the value of dedicated surveys in the absence of functional information systems.

## Abstract 2

Using data from three national surveys, Naeemah Abrahams and colleagues compare rates of femicide, intimate partner femicide and non-intimate partner femicide in South Africa between 1999 and 2017.

## Author summary

**Why was the study done?:**

- Femicide, the most severe form of gender-based violence, is not well described in middle- and low-income countries.
- Reliable routine data from police, justice and statistical departments are not available, and dedicated studies are needed to understand the scope of femicide in the country.

**What did the researchers do and find?:**

- South Africa has a methodogy to collect national data from forensic and police services to estimate femicide and repeated surveys on 3 occasions (1999, 2009, and 2017) allowed for the assessement of change over 18 years.
- We found that an estimated 3,793 women 14 years and older were victims of femicide in 1999, and this decreased to an estimated 2,407 femicide victims in 2017. We further found a decrease in intimate partner femicide with the age-standardised rates decreasing from 9.5/100,000 population in 1999 to 4.9/100,000 population in 2017.

**What do the findings mean?:**

- The 3 surveys over 18 years shows that femicide is preventable but that South Africa remains a country with the highest globally recorded rate of femicide.
- The reduction in femicide rates may be partially due to the contribution of activism of women and community-based organisations in bringing about change in Government’s gender-based violence policy and programme.
- A concern is the increase in missing data from police sources over the years, which points to the urgent need to improve information systems and integration of data across departments.

## Introduction

Femicide has been a relatively neglected area within the overall field of violence against women and girls. It was addressed for the first time at a global level in a 2013 resolution of the United Nations (UN) General Assembly “taking action against the gender-related killings of women and girls” (Resolution 68/191). A systematic review in the same year found that national data on the prevalence of IPF were only available from 66 countries, and these were mostly high-income. It concluded that 13.5% of all homicides were committed by intimate partners, with women 6 times more likely affected than men [1]. A recent report of the UN Office of Drugs and Crime (UNODC) used the term “femicide” to refer to the intentional killing of women with a gender-related motivation and clarified this as referring to murders by an intimate partner or a family member [2]. It concluded that 58% of all female murders globally were perpetrated by an intimate partner or a family member, a rate of 1.1/100,000 female population, and the highest rates were reported in Surinam (4.3/100,000 female population) and Belize (2.1/100,000 female population) [2]. In South Africa, information on IPF is not available from routine statistics such as from police, justice, and home affairs death registration databases [3,4]. However, in 2009, the rate measured in a national survey was 5.6/100,000, which was 5 times higher than the UNODC-estimated overall global rate [5].

This paper reports the findings of 3 national surveys on femicide in South Africa conducted across 18 years. We use the term “femicide” to refer to the intentional killing of women and girls. This includes intimate partner femicide (IPF), refering to the murder of a woman by a current or former intimate partner (e.g., husband or boyfriend), a definition aligned to that of intimate partner homicide used by, for example, the Office of Justice in the United States of America and the Australian Bureau of Statistics, as well as non-intimate partner femicide (NIPF) (i.e., killing by someone else) [6,7]. This differs from the UNODC definition above, and indeed definitional differences are a challenge for global comparisons [6]. The 2021 UNODC report mainly used countries’ statistical data but noted that, in most lower- and middle-income countries (LMICs), police administrative data are not a reliable source of information on IPF because perpetrator information is frequently not recorded; thus, the relationship between the victim and the perpetrator is not known [8].

In South Africa, the solution to the problem of data access has been circumvented by the development of a methodology for conducting national cluster sample surveys, and repetition of these surveys at intervals enabled femicide surveillance. This paper presents the findings of 3 surveys over 18 years and compares femicide, IPF, and NIPF between 1999 and 2009 and between 2009 and 2017 in South Africa.

## Methods

### Study background and data sources

In South Africa, “unlawful and intentional killing” is referred to as murder. We conducted 3 national retrospective studies of the murder of female victims aged 14 years and older, using a common approach in all respects with some variations (see details below). Our primary interest was to understand IPF and choose the starting age of 14 years as this is commonly the age when South African young women start dating. The definitions used in the study are shown in Box 1.

**Box 1. Femicide definitions used in the 3 South African Femicide Surveys**

Femicide–Murder of a woman

Intimate Partner Femicide (IPF)–Murder of a woman by an intimate partner (i.e., a current or ex-husband/boyfriend, same-sex partner, or a rejected would-be lover)

Non-Intimate Partner Femicide (NIPF)–Murder of a woman by someone other than an intimate partner (i.e., stranger, family member, acquaintance, etc.)

Our 2 main categories were IPF, e.g., women killed by a current or ex-intimate partner, and NIPF, included women killed by everyone else other than an intimate partner, which include family members as perpetrators. Two data sources were used in this study. The primary data source was medicolegal mortuary (MLM) records of murdered women, and the second source was data from police dockets based on the police investigation of each murdered women in the sample. The primary data source of MLM data was used because according to the Inquests Act of 1959, all cases of unnatural deaths must undergo a postmortem examination and records of all unnatural deaths, which include murder cases, are available from MLMs [9].

### Study design, sample, and sampling process

To draw national representative samples for each survey, we developed a sampling frame listing all MLMs operating in the country in the study year. The MLM was stratified by size based on the number of autopsies performed per annum, i.e., small MLM = <500 autopsies, medium size MLM = 500 to 1,499 autopsies performed per annum, and large MLM = >1,499 autopsies per year as shown in Table 1. The sample selection for all 3 surveys was done by the study statistician (CL).

**Table 1:** Sampling frame and sampling fraction used across the three surveys.

Mortuary strata	1999		2009		2017	
	Number of mortuaries	Sample (Sampling Fraction)	Number of mortuaries	Sample (Sampling Fraction)	Number of mortuaries	Sample (Sampling Fraction)
Small: <500 autopsies performed per year	176	12 (6.8)	81	20 (24.7)	96	55 (57.3)
Medium: 500–1,499 autopsies performed per year	34	5 (14.7)	33	13 (39.4)	31	19 (61.3)
Large: >1,499 autopsies performed per year	15	8 (53.3)	8	5 (55.5)	10	7 (70.0)
**Total**	225	25 (11.1)	122	38 (31.1)	137	81 (59.1)

The sampling frame was prepared in an MS Excel worksheet, and the relevant stratification was applied. Due to the stratification by size, simple random sampling within strata was used for selection of mortuaries using the uniform random number generator available in MS Excel. To avoid single-unit strata, a minimum number of the mortuaries per stratum was selected (*n* = 2). The stratification ensured that the sample was representative of both small rural mortuaries and larger ones attached to medical schools, and enhanced precision of national estimates. The number of MLM differed across the 3 studies. In the first study (1999), we based our assumption about the sample size on available surveillance data and a small study conducted in Gauteng [10].

The differences in the sampling frame was due to rationalisation of MLM after 1999 and ongoing operational changes to the forensic services, it meant that the number of mortuaries and their sizes differed for each of the 3 study years. In addition, the larger sampling fraction for each successive year was due to better research funding. While the sample size for the 1999 and 2009 studies was designed to produce national estimates, the 2017 sample size was intended to produce provincial estimates.

### Case identification and data collection

At the sampled mortuaries, we identified females (sex as recorded in the autopsy report) aged 14 and older who were registered between 1 January and 31 December in the year of study with death due to murder. We collected data from the MLM file and autopsy report for each case. Approval was granted by national and provincial police management who facilitated the identification of cases using the South African Police Services (SAPS) Crime Administration System (CAS) number documented in the MLM files to link each death with a police investigation docket. In 2017, we requested from the Research Ethics Committee to use personal identification number to assist in tracing murder cases in the police system if CAS numbers were absent. The research assistants sought consent from the police officers and, using a questionnaire, asked them questions about the case, which the police answered by extracting the information from the police dockets.

We excluded cases where both age and sex could not be determined and cause of death remained undetermined. Sex was unknown in 50 cases in the 2017 study, and these were usually cases which were decomposed bodies. We also excluded 9 cases where both age and sex were unknown. Autopsy reports include description of the deceased such as “adult” or “child,” and we used this information if date of birth was unknown and included adult female cases. These cases with no date of birth but identified as “adults” were excluded from the age group analysis. We also excluded if the cause of death was remained undetermined. In 2017, we decided to do interviews on the undetermined cause of death cases. A total of 502 cases identified at the mortuaries had undetermined cause of death. These were deaths related to poisoning, fire injuries, or deaths where the forensic report could not identify a cause, e.g., a woman found on her bed with no evidence of force entry or injuries on body. These cases were investigated by the police as “inquest cases,” and during police interviews, we found that 62 of the undetermined cases were murder cases. The remaining undetermined cases were excluded.

In 1999 and 2009, we used a paper-based questionnaire to collect data, and in 2017, we used REDCap, a web-based data entry tool [11]. At the MLMs, we collected data on age, cause of death (stab, gunshot, blunt force, etc.), CAS number, name of police station, samples taken, and if forensic evidence were collected. We also identified rape femicides (which could be in either and IPF or NIPF case) and collected data on whether rape was identified or suspected, i.e., a sexual assault evidence kit was used, victim’s underwear removed, or genital injuries.

The first author, as Principal Investigator, trained research assistants in all 3 studies to collect data at the MLMs and police. Research assistants were always supported by a study coordinator. In all studies, interviews with the police were either conducted in-person or telephonically with case files (dockets) the primary source of data from which the police officers extracted the data. The study team was not allowed to handle police dockets. We sought to interview the primary investigating officer, and, if this was not possible, a senior detective extracted the information from the case docket. A single police officer may have been investigating more than one murder (if more than one murder located at a police station) and often provided data on more than one case. We asked whether murder was suspected, additional victim information (education, employment), and information on the perpetrator, which revealed the victim–perpetrator relationship and was crucial to identify the case as an IPF (perpetrator was an intimate partner) or an NIPF (perpetrator was a non-partner). We also collected data on perpetrator age, sex, education, employment, known use of alcohol, firearm ownership, and prior convictions. For IPF perpetrators, we collected data on any prior history of IPV. We also gathered further information on the crime, its investigation, and legal outcome. For cases that were not definitively closed, we followed convention in the field of research on homicides used in the supplementary homicide data by the Bureau of Justice in the US where the perpetrator is defined as the person the Investigating Officer perceives to be primarily responsible [12].

In 1999, we collected data approximately 3 years after the deaths (in 2002 to 2003) in order to allow time for the legal cases to have been closed. In both the 2009 and 2017, studies data collection started within 2 years after the deaths. The 2017 data collection extended over a longer period ending only in November 2021, due to lockdown restrictions during the Coronavirus Disease 2019 (COVID-19) pandemic.

Race remains an important factor for risk profiles for violence and health in South Africa, and we define racial categories following conventions that were developed under Apartheid, which may not have the same meaning as for other countries and regions. It is acknowledged that the notion of race is a social construct, and in South Africa, “coloured” race refer to a mixed but a culturally specific group.

### Data management and statistical analysis

The protocol approved by the Ethics Committee include an analysis plan and is presented in the Supporting Information file (S1 Text). The analysis in this manuscript followed what was done in the analysis that compared 1999 and 2009 surveys [5]. The analysis related to imputing missing data on victim perpetrator relationships was not anticipated and not presented in the protocol analysis plan (S1 Text) and is described below. This change was necessitated by the large proportion of missing information due to the perpetrator not being identified by police. Data management and analysis for all the 3 studies were done in MS Excel and Stata 17. Procedures for cleaning and management were similar across the 3 studies. A difference was that a paper-based data collection process was followed in 1999 and 2009 studies, while a web-based data collection was followed in 2017. The PI (NA), senior statistician (CL), and CO PI (RJ) were involved in all 3 study procedures. In 2017, the statisticians (SM and EC) took more responsibilities for recoding, using the codes prepared in the 2 earlier studies.

Analysis weighting was based on the first 2 surveys designed to report on the national level only, and the strata were based on mortuary size. In 2017, additional funding allowed us to increase the sample size to allow for both national and provincial analysis. Province as a stratum allowed us to have femicide estimates at the provincial, which is critical for planning prevention and response to gender-based violence and femicide (GBVF) in the country as noted in the national strategic plan [13]. South African provinces are different across domains including population structures (age, education, etc.) and other socioeconomic factors including levels of crime [14,15]. Therefore, for the 2017 survey, stratification was done by mortuary size and province. The survey analysis weights were applied to account for the selection probabilities of mortuaries within survey strata and the sample realisation. Selection probabilities were based on the number of mortuaries randomly selected in each stratum of the total number of facilities.

Weight calculations were performed in Microsoft Excel using the formula:

*Weight = realisation weight (RW)* × *1/sampling fraction (SF)* × *1/ primary sampling unit selection probability (PSUP)*

where

RW = expected sample/realised sample for each mortuary

SF = 1/2 for mortuaries that had half their postmortem folders surveyed

SF = 1 for mortuaries that had all their postmortem folders surveyed

PSUP = number of mortuaries selected for stratum

total number of mortuaries in stratum

For analysis, we considered the 3 studies to be independent surveys due to the time separation and the independent sampling procedures used for each. All analysis procedures considered the multistage design of the study as described above with weighting stratified by mortuary size (and in 2017, also by province) and the mortuaries as clusters. After defining the datasets as survey data using “svyset,” “svy” command was used to account for stratification and weights. Taylor linearization was used to get robust variance estimators for parameter estimates.

To calculate the age-standardised rates (ASRs), we used the female population estimates derived from a mathematical model (Thembisa model version 4.4) for demographic statistics. This is a source used extensively for all government and adminstrative purposes at national and provincial levels [16,17]. We also used the World Health Organization (WHO) world standard population distribution normalised weightings for the ASR calculation to take into account the subgroup analysis for the ages 14 and above and for comparison with other countries [18]. The rates were calculated to account for missing ages (see details in Table 2 footnotes). To calculate rates by race, we used the midyear population estimates from Statistics South Africa [19].

**Table 2:** Age-standardised population rates for 1999, 2009, and 2017 for all female murders: IPF and NIPF by age and IRRs of population rate estimates between study years: weighted and imputed data.

	1999 Unweighted = 1,052 Weighted = 3,793			2009 Unweighted = 930 Weighted = 2,363			IRR of population rate estimates: 2009/1999 (95% CI)	2017 Unweighted = 1,301 Weighted = 2,407			IRR of population rate estimates: 2017/2009 (95% CI)
	N	Percent (95% CI)	Rate per 100,000 pop (95% CI)	N	Percent (95% CI)	Rate per 100,000 pop (95% CI)		N	Percent (95% CI)	Rate per 100,000 pop (95% CI)	
Overall female murders*	3,793		24.2 (15.5–32.9)	2,363		12.6 (8.5–16.6)	0.52 (0.48–0.56)	2,407		11.1 (9.8–12.4)	0.88 (0.81–0.95)
IPF	1,610	48.9 (42.0–55.7)	9.5 (6.4–12.7)	1,294	57.1 (52.7–61.6)	6.6 (5.3–8.0)	0.69 (0.63–0.77)	1,089	55.0 (51.6–58.4)	4.9 (4.1–5.8)	0.74 (0.66–0.83)
NIPF	1,686	51.1 (44.3–58.0)	11.4 (6.9–15.9)	971	42.9 (38.4–47.3)	5.4 (4.2–6.6)	0.47 (0.42–0.53)	890	45.0 (41.6–48.4)	4.2 (3.4–4.9)	0.78 (0.69–0.88)
Overall femicide by age group*											
14–29 y	1,359	35.8 (31.7–40.2)	6.8 (4.7–8.9)	867	36.7 (33.8–39.6)	3.8 (2.6–5.0)	0.56 (0.50–0.63)	943	39.2 (37.7–40.7)	4.2 (3.8–4.6)	1.11 (0.97–1.26)
30–44 y	1,310	34.5 (29.8–39.6)	8.1 (5.7–10.5)	784	33.2 (30.0–36.5)	4.2 (2.9–5.6)	0.52 (0.46–0.59)	774	32.2 (30.2–34.2)	3.4 (2.9–3.8)	0.81 (0.71–0.93)
45–59 y	512	13.5 (10.9–16.6)	4.4 (3.0–5.9)	408	17.3 (14.9–19.9)	2.5 (1.7–3.3)	0.57 (0.47–0.68)	365	15.2 (13.0–17.6)	1.8 (1.6–2.1)	0.72 (0.59–0.88)
60+ y	391	10.3 (6.4–16.4)	3.4 (1.2–5.7)	290	12.3 (9.9–15.1)	2.0 (1.3–2.7)	0.59 (0.48–0.73)	303	12.6 (11.2–14.2)	1.6 (1.4–1.8)	0.80 (0.64–1.00)
Undetermined age	222	5.8 (3.6–9.3)		15	0.6 (0.3–1.3)			22	0.3 (0.1–0.6)		
**IPF by age group**											
14–29 y	739	45.9 (38.3–53.4)	3.7 (2.7–4.7)	540	41.8 (37.1–46.4)	2.4 (1.9–2.8)	0.65 (0.56–0.76)	446	40.9 (37.7–44.2)	2.0(1.7–2.3)	0.95 (0.80–1.14)
30–44 y	579	35.9 (28.2–43.7)	3.6 (2.6–4.6)	534	41.3 (36.4–46.1)	2.9 (2.4–3.4)	0.81 (0.68–0.95)	417	38.3 (35.3–41.3)	1.8 (1.6–2.1)	0.78 (0.65–0.94)
45–59 y	127	7.9 (5.3–10.6)	1.1 (0.6–1.6)	156	12.1 (9.3–14.9)	1.0 (0.7–1.2)	0.91 (0.66–1.26)	137	12.5 (9.6–15.5)	0.7 (0.5–0.8)	1.00 (0.69–1.45)
60+ y	63	3.9 (1.1–6.7)	0.5 (0.1–1.0)	59	4.6 (2.6–6.5)	0.4 (0.2–0.6)	0.80 (0.49–1.31)	76	7.0 (4.9–9.0)	0.4 (0.3–0.5)	2.00 (0.84–4.75)
Undetermined age	102	6.4 (1.7–11.0)		4	0.3 (0.0–0.8)			14	1.3 (0.3–2.3)		
**NIPF by age groups**											
14–29 y	477	28.3 (22.2–34.4)	2.4 (1.5–3.2)	299	30.8 (26.9–34.6)	1.3 (1.0–1.6)	0.54 (0.44–0.66)	319	35.9 (32.4–39.3)	1.4 (1.2–1.6)	1.08 (0.86–1.34)
30–44 y	546	32.4 (26.1–38.7)	3.4 (2.4–4.4)	234	24.0 (20.0–28.0)	1.3 (1.0–1.5)	0.38 (0.31–0.47)	243	27.3 (22.9–31.7)	1.1 (0.8–1.3)	0.85 (0.66–1.09)
45–59 y	323	19.2 (14.4–23.9)	2.8 (1.7–3.9)	217	22.4 (18.4–26.4)	1.3 (1.0–1.7)	0.46 (0.37–0.59)	149	16.7 (13.4–20.1)	0.7 (0.6–0.9)	0.54 (0.40–0.72)
60+ y	271	16.1 (8.8–23.4)	2.4 (1.0–3.7)	222	22.8 (17.9–27.7)	1.5 (1.1–1.9)	0.63 (0.49–0.80)	171	19.2 (16.1–22.4)	0.9 (0.7–1.1)	0.60 (0.45–0.79)
Undetermined Age	69	4.1 (0.5–7.6)						8	0.9 (0.0–1.9)		
Overall femicide by race group*											
African	3,019	80.3 (67.0–89.1)	25.8 (14.4–37.3)	1,884	80.3 (69.1–88.1)	12.9 (9.2–16.5)	0.50 (0.46–0.54)	2,078	86.4 (83.9–88.5)	12.3 (11.2–13.5)	0.96 (0.88–1.05)
Coloured	516	13.7 (6.3–27.5)	37.5 (6.7–68.3)	309	13.2 (6.2–25.9)	17.7 (3.4–32.0)	0.47 (0.39–0.57)	233	9.7 (7.9–11.9)	11.9 (9.5–14.4)	0.67 (0.53–0.85)
Indian	41	1.1 (0.4–3.0)	9.7 (0.0–19.9)	31	1.3 (0.5–3.7)	5.9 (0.0–12.4)	0.61 (0.32–1.16)	21	0.9 (0.6–1.4)	3.6 (2.0–5.2)	0.60 (0.28–1.30)
White	183	4.9 (2.9–8.2)	9.3 (2.9–15.7)	116	4.9 (2.8–8.5)	5.5 (2.0–9.1)	0.60 (0.43–0.82)	72	3.0 (2.4–3.7)	3.4 (2.6–4.3)	0.62 (0.42–0.94)
**IPF by race group**											
African	1,255	78.2 (66.7–89.7)	10.7 (7.7–13.8)	1,041	80.6 (72.3–88.9)	7.1 (6.0–8.2)	0.66 (0.59–0.74)	948	86.5 (83.6–89.4)	5.6 (4.9–6.3)	0.79 (0.70–0.89)
Coloured	271	16.9 (5.7–28.1)	19.7 (5.6–33.7)	179	13.9 (5.1–22.6)	10.3 (3.4–17.2)	0.52 (0.40–0.68)	115	10.5 (7.7–13.3)	5.9 (4.2–7.5)	0.57 (0.41–0.79)
Indian	22	1.4 (0.2–2.6)	5.2 (1.0–9.4)	21	1.6 (0.4–2.8)	4.0 (1.1–6.9)	0.76 (0.33–1.76)	9	0.8 (0.1–1.6)	1.5 (0.1–2.9)	0.38 (0.13–1.13)
White	57	3.5 (1.9–5.2)	2.9 (1.4–4.4)	48	3.7 (1.8–5.7)	2.3 (1.1–3.5)	0.79 (0.46 1.35)	24	2.2 (1.3–3.1)	1.1 (0.7–1.6)	0.50 (0.25–0.99)
**NIPF by race groups**											
African	1,296	78.2 (66.4–90.0)	11.1 (7.3–14.9)	760	79.0 (70.2–87.9)	5.2 (4.2–6.2)	0.47 (0.41–0.53)	788	87.4 (84.8–89.9)	4.7 (4.2–5.2)	0.90 (0.78–1.03)
Coloured	225	13.6 (3.2–24.0)	16.4 (4.1–28.6)	126	13.1 (5.0–21.3)	7.2 (2.5–12.0)	0.44 (0.33–0.60)	83	9.2 (7.2–11.1)	4.2 (3.4–5.0)	0.59 (0.40–0.86)
Indian	15	0.9 (0.3–1.6)	3.6 (1.2–6.0)	10	1.1 (0.0–2.2)	1.9 (0.0–4.0)	0.53 (0.18–1.62)	0			
White	121	7.3 (3.9–10.6)	6.1 (2.8–9.4)	63	6.6 (3.2–10.0)	3.0 (1.5–4.5)	0.49 (0.32–0.75)	31	3.5 (2.3–4.6)	1.5 (1.0–2.0)	0.50 (0.27–0.90)

All analysis on ASRs was calculated using the Thembisa model except for race group that used Statistics South Africa midpopulation estimates.

**1999 female population**: Overall: 15,775,803; By Age: 14–29 years: 6,976,810; 30–44 years: 4,564,389; 45–59 years: 2,427,045; and 60 + years: 1,807,559.

**2009 female population**: Overall: 18,982,433; By Age: 14–29 years: 7,960,305; 30–44 years: 5,248,792; 45–59 years: 3,460,820; and 60 + years: 2,312,516.

**2017 female population**: Overall: 21,520,499; By Age: 14–29 years: 7,872,159; 30–44 years: 6,459,190; 45–59 years: 4,195,543; and 60 + years: 2,993,607.

For race groups age specific rates are reported using Statistics South Africa mid population estimates:

1999 female population: Overall: 15,458,162; By race: African: 11,683,651; Coloured: 1,375,413; Indian: 424,331; and White: 1,984,767.

2009 female population: Overall: 19,027,717; By race: African: 14,655,388; Coloured: 1,744,521; Indian: 529,557; and White: 2,098,251.

2017 female population: Overall: 21,501,234; By race: African: 16,862,856; Coloured: 1,958,404; Indian: 595,297; and White: 2,084,677.

ASRs were calculated to accounted for undetermined age by multiplying the ASR not accounting for undetermined age with a factor calculated by taking total number of female homicide cases divided by the total number of female homicide cases minus the total number of female homicide cases with undetermined age.

IRR is calculated by taking the fractions of the exposed and their total population. The 95% CIs are then adjusted from normal approximation calculation to account for the design effect of the survey.

* No imputation on overall descriptive summaries.

ASR, age-standardised rate; CI, confidence interval; IPF, intimate partner femicide; IRR, incidence rate ratio; NIPF, non-intimate partner femicide.

We presented overall femicide statistics and those for IPF and NIPF. To account for missing information due to the perpetrator not being identified by police, we used the Hot deck multiple imputation procedure to allocate cases to the IPF and NIPF groups [20]. The Hot deck imputation is a commonly used imputation method for surveys. It allowed for the multilevel structure of the data to be taken into account to maintain the clustering across the mortuaries. If this is ignored, the clustering is diluted, and, hence, the effective sample size is inflated. Thus, imputation was carried out within the province and mortuary size strata to preserve the study design. Resampling was straightforward in the large and medium sized mortuaries, but not in the small mortuaries. For the latter, we considered the resampling from the pooled province by place-of-injury data. Place of injury was included as a theoretically known variable that predicts intimate femicide and non-intimate femicide. It is known that intimate femicide deaths commonly occur in a home setting. The inclusion of place of injury in the imputation procedure was done to improve on the classification of unknown cases as intimate or non-intimate femicide cases. We compared the intraclass correlation coefficient (ICC), which is an indicator of the extent of clustering. Using standard univariate logistic regression multiple imputation, the ICC become much smaller, whereas the multiple Hot deck imputation maintained the ICC since it took multilevel design into account. This procedure was followed in all the 3 surveys, and 30 Hot deck imputations were used in each survey. In each survey, the 30 Hot deck multiple imputed datasets were then used to get pooled parameter estimates and their standard errors. Although Hot deck imputation has a limitation on the number of variables that can be included in the procedure and may not perform very well for large proportions of missing data as was the case for the 2017 survey, the multiple imputation and inclusion of a predictor of intimate femicide helped in improving the precision of the estimates.

We summarised all data, both imputed and non-imputed, using weighted counts and percentages and estimated the homicide ASRs for all female homicides in 1999, 2009, and 2017 and within femicide subgroups (IPF and NIPF). We calculated incidence rate ratios (IRRs) and their respective 95% confidence intervals (CIs) to compare rates between 2009 and 1999, and between 2017 and 2009. The standard errors for the rates and IRRs took into account the design effects of each survey.

This study is reported as per the Strengthening the Reporting of Observational Studies in Epidemiology (STROBE) checklist for cross sectional studies (see S1 STROBE Checklist).

### Ethical approval

Ethical approval for the study was granted by the Ethics Committee of the South African Medical Research Council (EC 008-5-2018), and further approval and access to data were obtained from the National and Provincial Departments of Health, the Forensic Pathology Service, and the South African Police Service. The study was based on people (the victims) who have died, and consent was therefore not required. Also, data for both victims and perpetrators were analysed anonymously.

## Results

### Weighted estimates and missing data

**Fig 1:**
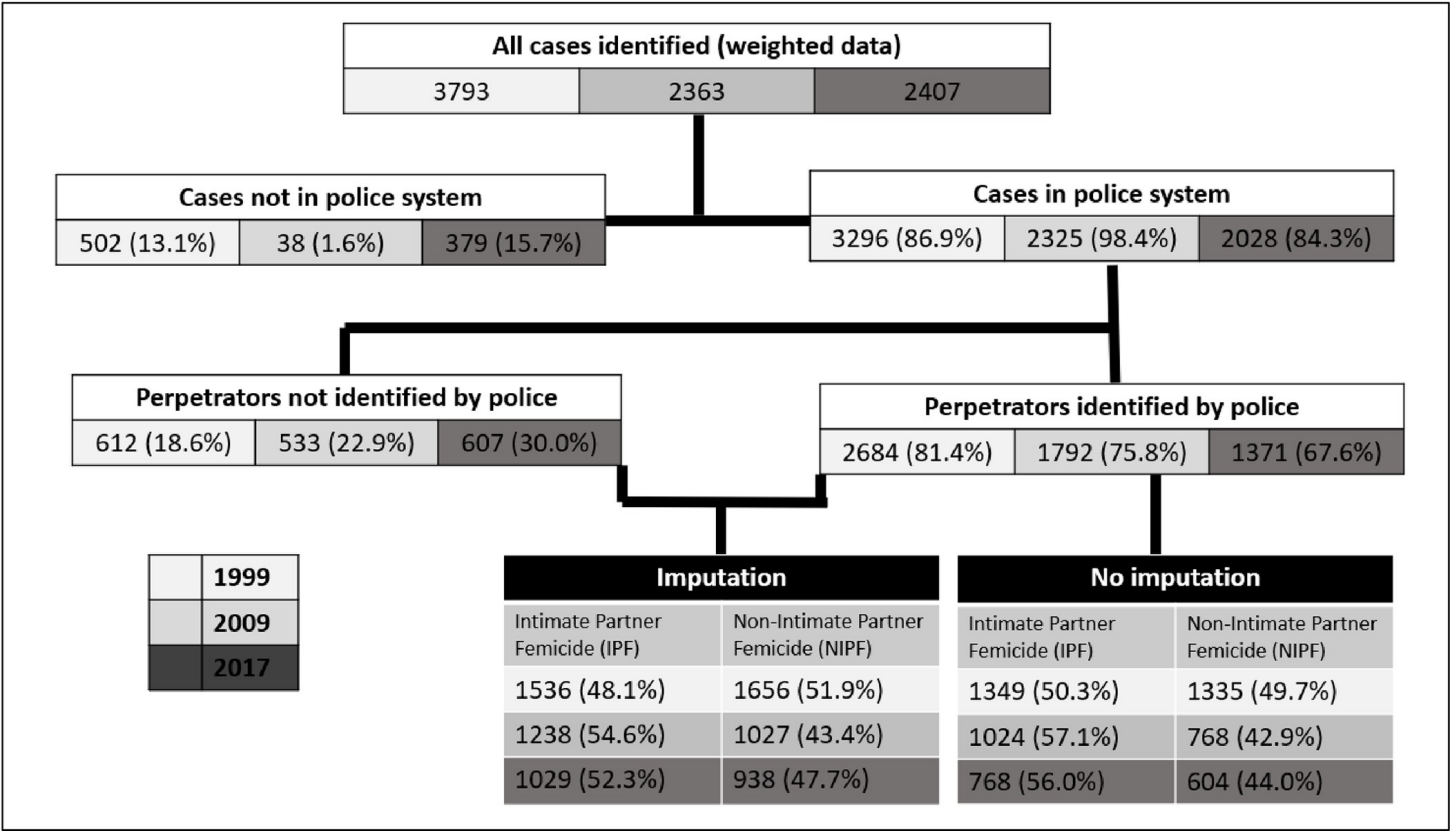
Flow chart of all estimated femicide cases of women 14 years and older across the 3 studies: weighted and imputed data.

The weighted estimates of the number of murdered women 14 years and older across the 3 studies are presented in Fig 1. All sampled mortuaries contributed data. The greatest proportion of cases with police data not traced in the police system or police interviews not done was found in 2017, with no police interviews done for 15.7% of the victims identified at the mortuaries. We, however, found similar proportion of missing data from police across the 3 strata of mortuaries (see S1 Table). Among the cases with police interviews done and investigation data identified, we found a successive increase in the proportion of perpetrators not identified by the police during the investigation, from 18.6% of cases in 1999 to 30% in 2017, hence the need for imputation.

### Age-standardised rates (ASRs) and incident rate ratios (IRRs)

Table 2 shows the age-standardised population rates (ASRs) for the different subgroups of femicide, as well as the IRRs of the population estimates comparing 1999 with 2009 and 2009 with 2017. In 2017, the ASR for overall femicide was 11.1/100,000 (95%CI 9.8 to 12.4) female population, the ASR for IPF was 4.9/100,000 (95% CI 4.1 to 5.8) female population, and that for NIPF was 4.2/100,000 (95% CI 3.4 to 4.9) female population. The ASRs for all femicides declined from 24.2/100,000 (95% CI 15.5 to 32.9) female population in 1999 to 12.6/100,000 (95% CI 8.5 to 16.6) female population in 2009. A similar reduction in IPF was found from 9.5/100,000 (95% CI 6.4 to 12.7) in 1999 to 6.6/100,000 (95% CI 5.3 to 8.0) in 2009. In 2017, the rate for IPF was 4.9/100,000 (95% CI 4.1 to 5.8) female population. A similar decrease was found in the NIPF rates, which were 11.4/100,000 (95% CI 6.9 to 15.9) in 1999, 5.4/100,000 (95% CI 4.2 to 6.6) in 2009, and 4.2/100,000 (95% CI 3.4 to 4.9) in 2017. The IRRs of the population estimates showed that the declines between 1999 and 2009 and 2009 and 2017 were statistically significant for all femicide types. However, it was notable that between 1999 and 2009, the decline for NIPF was greater than for IPF (IRR for NIPF 0.47 (95% CI 0.42 to 0.53)) compared to IRR for IPF 0.69 (95% CI 0.63 to 0.77). A similar difference by femicide type was not seen between 2009 and 2017 (IRR for IPF = 0.74 (95% CI 0.66 to 0.83)) versus IRR for NIPF = 0.78 (95% CI 0.69 to 0.88)).

### Age-standardised rates and incident rate ratios by age and race

In all the surveys, the IPF ASRs by age groups showed higher rates for the age groups 14 to 29 and 30 to 44 than for the age groups from 45 years and over. The decline in IPF from 1999 to 2009 was greater for younger women (aged 14 to 44 years) than older women (45 years old and older). A greater reduction in IPF was seen for the 14- to 29-year age group (IRR 0.69 (95% CI 0.63 to 0.77)) than for the 30- to 44-year group (IRR 0.81 (95% CI 0.68 to 0.95)) from 1999 to 2009. Between 2009 and 2017, the decline in IPF by age group was only significant for women aged 30 to 44 years (IRR 0.78 (95% CI 0.65 to 0.94)). There was a decline in NIPF between 1999 and 2009 across all age groups, and between 2009 and 2017, this was seen for the age groups of women aged 45 and over. The same analysis was done on the non-imputed data, and a similar pattern of decrease was shown (S2 Table).

### Provincial estimates and rates

We present the provincial estimates and rates of overall femicide, IPF, and NIPF from the 2017 study in Table 3. The Eastern Cape Province had the highest overall femicide rate at 22.3/100,000 female population, which was nearly double the province with the next highest rate, i.e., KwaZulu-Natal Province at 14.0/100,000 female population. The lowest rate was found in the Limpopo Province at 4.9/100,000 female population. The Eastern Cape Province also had the highest rates of both IPF and NIPF.

**Table 3:** Age-standardised population rates for 2017 for all female murders, IPF, and NIPF by South African Provinces: weighted and imputed data.

Provinces	Female Population 14 years+	Overall Female Murders		
		N (95% CI)	% (95% CI)	Rate/100,000 population (95% CI)
Western Cape	2,575,176	316 (309–323)	13.1 (12.0–14.3)	12.3 (12.0–12.5)
Eastern Cape	2,533,854	565 (506–624)	23.5 (20.9–26.3)	22.3 (20.0–24.6)
Northern Cape	389,291	43 (21–65)	1.8 (1.1–3.0)	11.1 (5.3–16.8)
Free-State	1,060,513	137 (117–158)	5.7 (4.8–6.7)	12.9 (11–14.9)
Kwa-Zulu Natal	4,165,842	583 (490–675)	24.2 (20.9–27.8)	14.0 (11.8–16.2)
North West	1,358,271	105 (97–113)	4.4 (4.1–4.7)	7.7 (7.1–8.3)
Gauteng	5,630,018	458 (299–617)	19.0 (14.2–25.0)	8.1 (5.3–11)
Mpumalanga	1,687,111	96 (69–123)	4.0 (3.0–5.3)	5.7 (4.1–7.3)
Limpopo	2,105,317	104 (84–124)	4.3 (3.5–5.3)	4.9 (4.0–5.9)
**Overall**	**21,520,499**	**2,407 (2,204–2,610)**	**100.0**	**11.2 (10.2–12.1)**
**IPF**				
Western Cape	2,575,176	125 (115–135)	49.9 (28.6–71.2)	4.9 (4.5–5.3)
Eastern Cape	2,533,854	203 (155–252)	44.7 (32.6–56.8)	8 (6.1–9.9)
Northern Cape	389,291	24 (11–38)	68.1 (25.7–100.0)	6.3 (2.8–9.7)
Free-State	1,060,513	62 (52–71)	54.3 (40.6–67.9)	5.8 (4.9–6.7)
Kwa-Zulu Natal	4,165,842	246 (190–302)	52.4 (44.4–60.3)	5.9 (4.6–7.3)
North West	1,358,271	50 (40–59)	58.5 (17.8–99.2)	3.7 (3–4.4)
Gauteng	5,630,018	216 (133–299)	58.1 (23.2–93.1)	3.8 (2.4–5.3)
Mpumalanga	1,687,111	53 (31–74)	62.5 (46.6–78.5)	3.1 (1.9–4.4)
Limpopo	2,105,317	49 (29–70)	49.1 (7.7–90.6)	2.3 (1.4–3.3)
**Overall**	**21,520,499**	**1,029 (911–1,146)**	**52.3 (49.1–55.5)**	**4.8 (4.2–5.3)**
**NIPF**				
Western Cape	2,575,176	126 (116–136)	50.1 (28.8–71.4)	4.9 (4.5–5.3)
Eastern Cape	2,533,854	252 (217–286)	55.3 (43.2–67.4)	9.9 (8.6–11.3)
Northern Cape	389,291	11 (6–17)	31.9 (0.0–74.3)	2.9 (1.5–4.4)
Free-State	1,060,513	52 (40–65)	45.7 (32.1–59.4)	4.9 (3.8–6.1)
Kwa-Zulu Natal	4,165,842	224 (175–273)	47.6 (39.7–55.6)	5.4 (4.2–6.6)
North West	1,358,271	35 (28–42)	41.5 (0.8–82.2)	2.6 (2.1–3.1)
Gauteng	5,630,018	156 (119–193)	41.9 (6.9–76.8)	2.8 (2.1–3.4)
Mpumalanga	1,687,111	31 (22–41)	37.5 (21.5–53.4)	1.9 (1.3–2.4)
Limpopo	2,105,317	51 (32–70)	50.9 (9.4–92.3)	2.4 (1.5–3.3)
**Overall**	**21,520,499**	**938 (862–1015)**	**47.7 (44.5–50.9)**	**4.4 (4.0–4.7)**

CI, confidence interval; IPF, intimate partner femicide; NIPF, non-intimate partner femicide.

### Mechanisms of death

We present trends in the 3 main mechanisms of death in Table 4. Death by gunshot showed a decrease for all forms of femicide between 1999 and 2009; however, this did not continue between 2009 and 2017 for NIPF. Similarly, there was a decrease in rates of death by stabbing for all forms of femicide between 1999 and 2009; however, an increase was found for all femicides between 2009 and 2017 (IRR 1.17 (95% CI 1.02 to 1.35)). Rates of death from blunt force injuries showed a continued decline with a difference found for all forms of femicide. Overall, a higher proportion of IPF victims died from blunt force injuries compared to the NIPF group. The analysis on the non-imputed data shows similar patterns of distribution and decreases (S3 Table).

**Table 4:** Age-standardised population rates for 1999, 2009, and 2017 for all female murders, IPF, and NIPF by the mechanism of death and IRRs of population rate estimates between study years: weighted and imputed data.

Characteristics	1999			2009			IRR of population rate estimates: 2009/1999 (95% CI)	2017			IRR of population rate estimates: 2017/2009 (95% CI)
	Overall Unweighted = 1,052 Overall Weighted = 3,793 IPF Weighted = 1,610 NIPF Weighted = 1,686			Overall Unweighted = 930 Overall Weighted = 2,363 IPF Weighted = 1,294 NIPF Weighted = 971				Overall Unweighted = 1,301 Overall Weighted = 2,407 IPF Weighted = 1,089 NIPF Weighted = 890			
	N	Percent (95% CI)	Rate per 100,000 pop (95% CI)	N	Percent (95% CI)	Rate per 100,000 pop (95% CI)		N	Percent (95% CI)	Rate per 100,000 pop (95% CI)	
**Firearm deaths**											
All female murders*	1,147	33.4 (24.9–43.1)	7.2 (2.7–11.7)	462	19.5 (15.1–25.0)	2.4 (1.2–3.6)	0.33 (0.29–0.39)	563	23.4 (22.2–24.7)	2.6 (2.1–3.1)	1.08 (0.91–1.29)
IPF	495	31.9 (22.1–41.8)	2.9 (1.4–4.5)	259	20.0 (14.9–25.1)	1.3 (0.8–1.9)	0.45 (0.36–0.55)	215	19.8 (16.6–22.9)	1.0 (0.6–1.4)	0.77 (0.60–0.99)
NIPF	556	34.7 (24.8–44.5)	3.8 (1.5–6.0)	190	19.6 (14.6–24.6)	1.0 (0.5–1.5)	0.26 (0.21–0.33)	213	24.0 (21.0–26.9)	1.0 (0.7–1.3)	1.00 (0.76–1.31)
**Stab injury deaths**											
All female murders*	1,049^¥^	30.5 (24.0–37.9)	6.6 (3.1–10.0)	668	28.3 (23.6–33.5)	3.5 (2.0–5.0)	0.53 (0.46–0.61)	897^¥¥^	37.6 (35.8–39.4)	4.1 (3.4–4.8)	1.17 (1.02–1.35)
IPF	468	30.2 (23.7–36.7)	2.8 (1.3–4.4)	356	27.5 (22.7–32.3)	1.8 (1.2–2.4)	0.64 (0.53–0.78)	423	38.9 (35.5–42.3)	1.9 (1.4–2.4)	1.06 (0.87–1.28)
NIPF	509	31.8 (23.0–40.6)	3.3 (1.6–5.1)	301	31.0 (25.7–36.4)	1.6 (1.1–2.2)	0.48 (0.40–0.59)	331	37.2 (33.5–40.9)	1.5 (1.1–2.0)	0.94 (0.75–1.16)
**Blunt force injury deaths**											
All female murders*	943	24.9 (17.7–33.8)	6.0 (2.5–9.6)	580	24.5 (20.5–29.0)	3.1 (1.7–4.5)	0.52 (0.45–0.60)	501	20.8 (19.3–22.3)	2.3 (1.9–2.8)	0.74 (0.63–0.88)
IPF	515	32.0 (21.5–42.5)	3.0 (1.4–4.7)	354	27.4 (23.0–31.7)	1.8 (1.1–2.5)	0.60 (0.50–0.72)	250	24.6 (21.6–27.5)	1.1 (0.8–1.4)	0.61 (0.49–0.77)
NIPF	350	20.8 (13.0–28.5)	2.5 (0.7–4.3)	209	21.5 (17.1–25.9)	1.2 (0.6–1.8)	0.48 (0.38–0.61)	161	16.7 (13.6–19.7)	0.8 (0.5–1.0)	0.67 (0.50–0.89)

**1999 female population 14 years and older:** Overall: 15,775,803.

**2009 female population 14 years and older**: Overall: 18,982,433.

**2017 female population 14 years and older**: Overall: 21,520,499.

^¥^*N* = 3,439 (*n* = 354 missing data on stab injury).

^¥¥^*N* = 2,387 (*n* = 20 missing data on stab injury).

* No imputation on overall descriptive summaries.

CI, confidence interval; IPF, intimate partner femicide; IRR, incidence rate ratio; NIPF, non-intimate partner femicide.

### Murder, victim, and perpetrator characteristics

Table 5 presents further aspects of the IPF and NIPF murders. There was a decrease in the proportion of rape femicides between 2009 and 2017 for both IPF and NIPF, but these remained much more common among the NIPF compared to IPF cases. In 2017, the proportion of overall cases that were closed with the perpetrators convicted of murder was approximately 1 in 3. The proportion of perpetrators convicted was significantly higher for NIPF cases in 2017 compared to 2009. There was no change in the proportion of IPF cases with convictions across the 3 surveys. The profile of perpetrators of NIPF shows acquaintances/friends known by sight to be the most common perpetrators across the 3 studies with strangers the second most common perpetrators (Table 6). Approximately 1 in 7 of the perpetrators of IPF died by suicide within 6 days of the murder. This proportion is unchanged across the 3 data points and is much higher than the proportion of NIPF perpetrators who died by suicide. A history of IPV provided by the police during the interviews shows similar patterns across the surveys, with a slight decrease in 2017, which was not statistically significant.

**Table 5:** Aspects of the murder and the case legal outcome over the 3 studies for IPF and NIPF: weighted and imputed data.

Characteristic	IPF			NIPF		
	1999 *N* = 1,610 % (95% CI)	2009 *N* = 1,294 % (95% CI)	2017 *N* = 1,089 % (95% CI)	1999 *N* = 1,686 % (95% CI)	2009 *N* = 971 % (95% CI)	2017 *N* = 890 % (95% CI)
Rape femicides	13.6 (8.7–18.4)	14.6 (11.6–17.7)	4.9 (3.3–6.5)	17.0 (9.4–24.6)	28.7 (23.2–34.2)	12.1 (8.8–15.4)
Perpetrator convicted	29.4 (20.7–38.2)	29.7 (23.7–35.7)	35.7 (32.6–38.7)	25.9 (19.6–32.3)	18.3 (14.2–22.4)	33.5 (30.2–36.8)
Perpetrator died by suicide	14.0 (9.1–18.8)	14.5 (11.7–17.3)	12.9 (10.9–14.9)	2.5 (0.1–4.8)	2.7 (1.1–4.3)	1.8 (1.1–2.5)
History of IPV	31.6 (21.9–41.3)	33.2 (27.7–38.6)	28.6 (25.0–32.2)			

IPF, intimate partner femicide; IPV, intimate partner violence; NIPF, non-intimate partner femicide.

**Table 6:** Victim and perpetrator characteristics over the 3 surveys for IPF and NIPF: weighted and non-imputed data.

Characteristic	IPF			NIPF		
	1999: *N* = 1,349 % (95% CI)	2009: *N* = 1,024 % (95% CI)	2017: *N* = 768 % (95% CI)	1999: *N* = 1,335 % (95% CI)	2009: *N* = 768 % (95% CI)	2017: *N* = 604 % (95% CI)
**Victim perpetrator relationship**						
Husband-current	17.1 (11.3–25.1)	17.1 (13.9–21)	17.1 (15.6–18.7)			
Ex-husband	1.3 (0.6–2.7)	0.7 (0.3–2.1)	1.2 (1.1–1.3)			
Cohabitating boyfriend-current	49.6 (39.7–59.5)	23.8 (17.2–32)	19.4 (15.8–23.5)			
Ex cohabitating boyfriend	0.6 (0.3–1.2)	1.1 (0.5–2.5)	0.6 (0.3–1.3)			
Boyfriend/girlfriend-current*	28.3 (21.9–35.9)	47.8 (41.3–54.5)	53.6 (48.7–58.5)			
Ex-boyfriend	2.8 (1.5–5.2)	7.4 (5.8–9.3)	7.2 (5.9–8.9)			
Rejected man proposing a relationship	0.3 (0.1–0.7)	2.0 (1.1–3.5)	1.0 (0.4–2.2)			
Family**				14.2 (8.2–23.5)	18.7 (14.7–23.5)	17.4 (15.1–19.9)
Friend/known by sight/acquaintance				51.1 (44.4–57.6)	53.6 (48.3–58.9)	42.1 (37.4–46.9)
Stranger/unknown				33.9 (26.9–41.6)	19.9 (15.3–25.4)	31.8 (27.3–36.7)
Other***				0.8 (0.5–1.3)	7.8 (5.8–10.5)	8.8 (6.9–11.0)
**Injury setting**						
Major urban	46.9 (30.3–64.2)	32.2 (26.2–38.8)	33.4 (28.0–39.3)	44.8 (27.2–63.9)	33.6 (26.4–41.7)	32.8 (29.5–36.4)
Rural	53.1 (35.8–69.7)	64.2 (57.5–70.4)	65.9 (59.9–71.4)	55.2 (36.1–72.8)	64.1 (56.0–71.4)	67.2 (63.6–70.5)
Unknown		3.6 (2.5–5.3)	0.7 (0.3–1.7)		2.3 (1.5–3.4)	
**Suspected rape murder**						
No	88.8 (84.8–91.9)	84.7 (81.3–87.5)	97.1 (96–97.9)	87.2 (80.1–92.0)	68.5 (62.2–74.2)	87.4 (83.2–90.7)
Yes	11.2 (8.1–15.2)	10.5 (8.3–13.2)	2.9 (2.1–4)	12.8 (8.0–19.9)	27.4 (22.6–32.9)	12.6 (9.3–16.8)
Missing info		4.8 (3.3–7)			4.1 (2.3–7.1)	
**Perpetrator convicted**						
No	64.9 (54.9–73.7)	62.5 (55.9–68.7)	49.4 (45.7–53.2)	67.2 (58.7–74.7)	76.9 (71.4–81.6)	50.6 (46.9–54.4)
Yes	35.1 (26.3–45.1)	37.5 (31.3–44.1)	50.6 (46.8–54.3)	32.8 (25.3–41.3)	23.1 (18.4–28.6)	49.4 (45.6–53.1)
**Perpetrator died by suicide**						
No	83.4 (76.7–88.4)	80.8 (76.5–84.5)	86.9 (84.8–88.8)	96.9 (92.1–98.8)	95.6 (92.4–97.5)	97.8 (96.8–98.5)
Yes	16.6 (11.6–23.3)	18 (14.6–21.9)	12.9 (11.0–15.0)	3.1 (1.2–7.9)	3.2 (1.8–5.7)	1.8 (1.2–2.7)
Missing info		1.2 (0.5–2.9)	0.2 (0.1–0.6)		1.2 (0.5–2.7)	0.3 (0.1–1.4)
**History of IPV**						
No	68.4 (58.0–77.2)	66.9 (61.2–72.1)	68.8 (65.2–72.2)			
Yes	31.6 (22.8–42)	33.1 (27.9–38.8)	28.1 (24.7–31.8)			
Missing info			3.1 (2.3–4.0)			

*Current boyfriend includes same-sex partner: 1999 IF includes 9 same-sex partners; 2009-no same-sex partner reported; 2017–1 same sex partner.

**Family includes mother, father, brother, sister, stepfather/mother’s boyfriend, relatives, and in-laws.

***Other includes female perpetrator romantically involved with current/ex-husband/boyfriend (love triangle), current/ex-client/customer, tenant, community members, employee (gardener, caregiver, and domestic worker), and colleague.

CI, confidence interval; IPF, intimate partner femicide; IPV, intimate partner violence; NIPF, non-intimate partner femicide.

## Discussion

In 2017, the rate of IPF was 4.9 per 100,000 female population, which is more than 4 times the estimated global rate for intimate partner and family-related homicide, and higher than the rate for any other country [2]. However, South Africa is the only country that has collected femicide data with a dedicated national survey, and it is possible that rates in other countries would be shown to be higher with more accurate estimations. More women were murdered by their intimate partner than by other persons in 2009 and 2017. We have shown a decline in all forms of femicide in South Africa between the 1999 and 2009 and 2009 and 2017 studies. The analysis by age group shows that women aged 14 to 44 are most at risk of IPF; however, this age group also benefitted the most from the decrease in IPF, which contrasts with NIPF where the decrease initially impacted all age groups, but between 2009 and 2017 was only seen among older women (45 years and older). There is some evidence that the decline in IPF may have initially benefitted the youngest women most, with a greater decline seeing in women aged 30 to 44 after 2009 than in other age-groups. The decline in IPF was visible across African and Coloured groups but was most notable for Coloured women who had the highest race subgroup rates in 1999 and 2009. The decline for African women for NIPF was not sustained from 2009 to 2017.

We have also shown that the rate in some provinces of the country is exceptionally high, with more than a 4-fold variation in rates among provinces. Notably, in no province is the rate low by international comparison. The provinces with higher rates, in particular Eastern Cape and Kwa-Zulu Natal, are known to have multiple challenges across social, economic, and political sectors [21]. These results are important for planning responses and prevention intervention at the provincial level, and the 2017 estimates provide a baseline to assess future changes. Provincial data can also be used by advocates and provincial government structures to mobilise resources for provinces that requires them the most. We have also seen a decrease in IPF by gunshot between 2009 and 2017, but no change in gun-related NIPF. There was a decrease in femicide by gunshot between 1999 and 2009, which paralleled an overall decrease in gun-related murders among both men and women that has been widely attributed to effective implementation of the Firearm Control Act [22,23]. However, a general increase in corruption and lawlessness after 2009 has resulted in a relaxation of gun control with greater circulation of licensed and unlicensed firearms [24]. Our findings suggest that these were particularly used in NIPFs, which generally are perpetrated in the context of other crime, and this coincides with an increase in other firearm-related deaths and injuries [22,23]. This shows the value of strict control of gun ownership and licensing in reducing gun-related murder. The increase in the overall murder by stabbing mirrors an increase that has been seen in male homicides [25], the reason for this is unclear.

The decline in femicide cannot be attributed to improved performance of the police and criminal justice system as there was no evidence of this having occurred, and, notably, there was no change in the proportion of IPF in which there was conviction of the perpetrator across the 3 surveys. The decrease in the number of cases in which a perpetrator was identified and the increase in the number of missing dockets are evidence of a decline in the quality of case investigation and management. These problems with policing and undermining of the criminal investigation capabilities are well documented and reflect poor management and corruption [26].

The decrease in femicide overall mirrors changes that have been noted in deaths from interpersonal violence of men. The latter declined from 25,886 in 1997 to 15,854 in 2012, a 52.4% decline in the age-standardised death rate [27]. The changes in femicide in part reflect overall changes in the country that have resulted in a reduction in the use of violence and murder. These were driven by the end of apartheid (1994) and improved social conditions and access to the economy for the previously marginalised majority [28]. However, our analysis has shown that IPF and NIPF are distinct social phenomena, with different patterns of prevalence across the 3 data points, by age group and by race, and they have differences in underlying drivers. For example, between 1999 and 2009, the IRRs show that NIPF declined faster than IPF, and across all age-groups, whereas the IRRs for IPF only declined for women under 45. It was also seen across femicide types for African and Coloured women, but among African women, IPF declined less than NIPF. Between 2009 and 2017, IPF only declined significantly for women aged 30 to 44 years, whereas the greatest decline in NIPF was seen for women aged 45 years and over. Studies have consistently shown younger women to be at higher risk for IPV [29]. The extent of poverty and social norms around the use of violence may impact both IPF and NIPF, but IPF is also importantly driven by IPV and social norms around gender equality [21]. The patterns of change in IPF fit well with what is known about changes in social norms around gender equity and the acceptability of IPV that have occurred in the country, that have seen great reductions in acceptability of IPV from 1998 to 2016, but more traditional views on the acceptability of wife beating held by African women living in rural settings may contribute to slower change [15].

The femicide surveys are currently the only national data on IPV for South Africa. They provide the most comprehensive insights into the likely impact of national efforts to eliminate violence against women that have been conducted over the last 2 decades. The research has been conducted against a backdrop of a decrease in the overall rate of murder (South African legal term) in the country since 1994 [14]. While this led to a reduction in female murder overall, it raised a key question about whether the murder of women by intimate partner perpetrators would be just as preventable as murder by non-intimate partners. In South Africa, combatting violence against women has been a national priority since the advent of democracy in 1994. One of the first laws passed by the Government was the Domestic Violence Act, signed into law in 1998, which included provisions for access to protection orders [30]. The law was a major focus of activism in 1998 led by the Soul City Institute and linked to an edutainment television series on domestic violence, which at the time had 8 million viewers. In the early 2000s, there was considerable activism around demands for public access to postexposure prophylaxis for HIV after rape. Activism around rape heightened from 2005 to 2006 when the then Deputy-President, Jacob Zuma, was charged and tried for rape. As he defended himself, rape and violence against women became a major national focus for debates and anti-rape activism. This provided impetus for the 2007 amendments to the Sexual Offences legislation, which substantially widened the definition of rape [30]. In 2013 and 2014, there was a similar national focus on femicide when the para-Olympic sprinter Oscar Pistorius was charged with, and tried for, the murder of his girlfriend. Gender-based violence activism continued and came to the fore in 2018, resulting in the President convening a national summit and establishing a process that led to the adoption of a National Strategic Plan to Combat Gender-based Violence and Femicide (GBVF) in 2020 [13]. Throughout these years, violence against women has been kept in the public eye by the media and women’s organisations especially during Women’s Month in August and the 16 days of Activism in November each year, as well as the periodic release of research results, and many Government Departments have developed strategies for addressing it. The South African experience of feminist activism that led to gender-based violence (GBV) policy change is not unique. Indeed, in a systematic review by Weldon and Htun [31], the authors reviewed the GBV policies of 70 countries and found women’s right movements were the most consistent factor that brought about GBV policy change.

Thus, it seems likely that the decrease in IPF indicates progress in reducing IPV in South Africa. Despite the considerable amount of research on IPV conducted over more than 30 years, which include prevalences studies among women as victims and men as perpetrators [32,33], studies within marginalised and high risk groups [34,35], risk factor studies [36], the large body of research on development and testing of primary prevention interventions [37,38], and the impact of IPV on women’s health (e.g., HIV, mental health) [39–41], the country has no surveillence through national IPV prevalence surveys, and the research on IPF represents the only IPV surveillance within the country. We do not know if the decline in IPF mirrors a similar decline in all forms of IPV in the country over the 18 years, but we would expect that there should have been a decrease in IPV for this to have been observed. Given the broad range of responses to GBV, it is impossible to pinpoint moments when activities contributed to the decline in femicide; rather, it is likely that the sum total has influenced a decline in violence, as has been shown in other countries [42]. This is in line with a review of GBV policy change in 70 countries that showed the crtical role of local feminist movements in changing patriarchal gender norms [31]. Many high-income countries have shown a decrease in femicide at a population level [8]; however, to the best of our knowledge, this is the first time that similar declines have been described through national surveillance in a middle-income country. An important conclusion of our research, taken together with that from other countries, is that IPF is preventable at a population level, and we suspect it provides further evidence that real shifts in population prevalence of IPV can occur when countries focus on it as a national problem.

Our study’s strength is our use of a consistent methodology conducted over 3 studies spanning 18 years and which was initially based on a study design developed by author Martin [43]. We also show the value of dedicated surveys in the absence of functional information systems, and we support the call from UN bodies for focused attention to the collection of data to improve government responses to killing of women and to allow for comparisons across communities and regions [44]. A study limitation includes the variability in sampling across the 3 years; however, it is very unclear what the impact of this would have been. We also considered the role of missing data in our estimates. Firstly, it is possible that we missed murder cases that were not processed by the official MLMs of the country or we missed cases that were misclassified as undetermined cause of death. We, however, believe this would have been only a few cases as all unnatural deaths in the country must undergo a postmortem examination and a recent injury mortality report show reasonable congruency between murders reported by SAPS and forensic data [25]. Undetermined cause of death is a well-described phenomenon of death certification in South Africa, and the 2017 Injury Mortality Study reported 11.2% of unnatural deaths remaining undetermined. A second aspect of missing data are the cases not traced in the police system. The proportion of cases in 2017 that were not in the police system was higher than the proportion in 2009, although similar to that in 1999. Access to the police was more challenging in the 2017 study due to COVID-19, as this limited the research team’s ability to visit the police stations and assist the police staff in locating dockets, and this may have contributed to higher number of non-traceable police cases. Although we relied on the CAS number, we discovered many challenges with CAS numbers being changed or documented incorrected (inconsistent allocation of CAS numbers), and we requested ethical approval to use ID numbers as an additional tracing tool. For some cases where a SAPS investigation was not identified, i.e., a CAS and ID number not found on the police data systems, we requested SAPS to open an investigation. This, however, happened at the end of the study, and any further perpetrator data identified by the police could not be included in our database. The inability to not trace the investigation of cases within the police system (15.7% in 2017) may have contributed to the underestimation of IPF and NIPF as these cases were not included in this analysis. The third aspect of missing data related to cases where a police investigation had occurred, but the investigation process was unable to identify a perpetrator. This may also have had a potential impact on the estimates of NPF and NIPF. We used imputation (details described in methods) as this approach was robust since we had very good information on a large number of cases. We calculated rates on non-imputed data and a similar pattern of decline across the 3 surveys was found for IPF and NIPF. (See S2 and S3 Tables for non-imputed results.) The improvement of an integrated information system with linkages between data sets (police, justice, forensic, etc.) is a key strategic objective in the recently developed Femicide Prevention Strategy developed for South Africa. This strategy is awaiting final Government approval [3].

We also mostly used the population data from the Thembisa model for calculation of the ASR, but because of unavailability of race population data, we used the Statistics South Africa midyear population data for the calculation of rates by race group. There were minimal differences between overall population data across the 2 population data sources (Table 2 footnote).

We also acknowledge that the suspect identified by the police may not have always been the one responsible in some cases, but we have been consistently aware of this possibility in our research and have formed our opinion on whether a women was killed by a partner or a non-partner based on a description of circumstances of the death. It is well known that convictions are not possible in cases with insufficient evidence and depending on a final court outcome will result in underestimations [26]. Our estimates for 1999 and 2009 differ very slightly from what has been previously published without using imputation. However, the non-imputed data presented in the supplemental file shows similar patterns and statistically significant reductions between studies for most of the analysis. We believe imputation allowed us to get closer to describing the full dimension of femicide in the country.

We have shown that South Africa has the highest globally recorded rate of femicide, and yet there have been large declines in all types of femicide over a period spanning 18 years. This shows that femicide can very likely be impacted by important social dynamics, including overall increases in political stability and less general violence, as well as economic well-being, but furthermore that activism, policies, and programmes to build greater gender equity and combat the violence against women, can result in important reductions in IPF, and we suspect underlying levels of IPV. The public health implications point to the huge importance of population-level structural interventions in reducing levels of femicide as well as the need to support feminist movements and community activism against violence against women.

## Supporting information

S1 STROBE Checklist STROBE statement. (DOCX)

S1 Text Research protocol. (PDF)

S1 Table Count and percentages distribution of all female murder cases in the police system by mortuary strata across the 3 surveys. (DOCX)

S2 Table Age-standardised population rates for 1999, 2009, and 2017 for overall female murders: IPF and NIPF by age and race and IRRs of population rate estimates between surveys: weighted and non-imputed data. IPF, intimate partner femicide; IRR, incidence rate ratio; NIPF, non-intimate partner femicide.
(DOCX)

S3 Table Age-standardised population rates for 1999, 2009, and 2017 for all female murders:·IPF and NIPF by death mechanism and incidence. IPF, intimate partner femicide; NIPF, non-intimate partner femicide.
(DOCX)

## Abbreviations

ASR: age-standardised rate
CAS: Crime Administration System
CI: confidence interval
COVID-19: Coronavirus Disease 2019
GBV: gender-based violence
GBVF: gender-based violence and femicide
ICC: intraclass correlation coefficient
IPF: intimate partner femicide
IPV: intimate partner violence
IRR: incidence rate ratio
MLM: medicolegal mortuary
NIPF: non-intimate partner femicide
SAPS: South African Police Services
UN: United Nations
UNODC: UN Office of Drugs and Crime
WHO: World Health Organization
