# Supplementary material for: Femicide, intimate partner femicide, and non-intimate partner femicide in South Africa: An analysis of 3 national surveys, 1999–2017
Source: PLoS Med. 2024 Jan 18;21(1):e1004330. doi: 10.1371/journal.pmed.1004330 (PMC10796052; doi:10.1371/journal.pmed.1004330)
Supplement: S1 STROBE Checklist — (DOCX) [file pmed.1004330.s001.docx]

S1 Check List STROBE Statement

|  | Item No | Recommendation | Section and paragraph |
| --- | --- | --- | --- |
| **Title and abstract** | 1 | (*a*) Indicate the study’s design with a commonly used term in the title or the abstract | Tittle & Abstract |
|  |  | (*b*) Provide in the abstract an informative and balanced summary of what was done and what was found | Abstract |
| Introduction | | | |
| Background/rationale | 2 | Explain the scientific background and rationale for the investigation being reported | Introduction |
| Objectives | 3 | State specific objectives, including any prespecified hypotheses | Introduction:  Last paragraph |
| Methods | | | |
| Study design | 4 | Present key elements of study design early in the paper | Study background and data sources: First paragraph |
| Setting | 5 | Describe the setting, locations, and relevant dates, including periods of recruitment, exposure, follow-up, and data collection | Introduction  Last paragraph |
| Participants | 6 | (*a*) Give the eligibility criteria, and the sources and methods of selection of participants | Study background and data sources: First paragraph |
| Variables | 7 | Clearly define all outcomes, exposures, predictors, potential confounders, and effect modifiers. Give diagnostic criteria, if applicable | Case Identification and data collection: paragraph 1-3 &6 |
| Data sources/ measurement | 8* | For each variable of interest, give sources of data and details of methods of assessment (measurement). Describe comparability of assessment methods if there is more than one group | Case Identification and data collection:  Paragraph 1-2 |
| Bias | 9 | Describe any efforts to address potential sources of bias | Data management and statistical analysis : paragraph 1,4-5. |
| Study size | 10 | Explain how the study size was arrived at | Study Design, sample and sampling process: paragraph 1-3. |
| Quantitative variables | 11 | Explain how quantitative variables were handled in the analyses. If applicable, describe which groupings were chosen and why | Data management and statistical analysis: paragraph 1-5 |
| Statistical methods | 12 | (*a*) Describe all statistical methods, including those used to control for confounding | Data management and statistical analysis: paragraph 2-3 |
|  |  | (*b*) Describe any methods used to examine subgroups and interactions | Data management and statistical analysis: paragraph 5 |
|  |  | (*c*) Explain how missing data were addressed | Data management and statistical analysis: paragraph 5 |
|  |  | (*d*) If applicable, describe analytical methods taking account of sampling strategy | Data management and statistical analysis: paragraph 2 |
|  |  | (*e*) Describe any sensitivity analyses | Data management and statistical analysis: paragraph 6 |
| Results | | | |
| Participants | 13* | (a) Report numbers of individuals at each stage of study—eg numbers potentially eligible, examined for eligibility, confirmed eligible, included in the study, completing follow-up, and analysed | Fig 1  Case identification and data collection:  Paragraph 2 |
|  |  | (b) Give reasons for non-participation at each stage | NA |
|  |  | (c) Consider use of a flow diagram | Fig1 |
| Descriptive data | 14* | (a) Give characteristics of study participants (eg demographic, clinical, social) and information on exposures and potential confounders | Table2-6 |
|  |  | (b) Indicate number of participants with missing data for each variable of interest | Fig1  Table 6 |
| Outcome data | 15* | Report numbers of outcome events or summary measures | NA |
| Main results | 16 | (*a*) Give unadjusted estimates and, if applicable, confounder-adjusted estimates and their precision (eg, 95% confidence interval). Make clear which confounders were adjusted for and why they were included | Table S2 and S3 provides information on unadjusted data |
|  |  | (*b*) Report category boundaries when continuous variables were categorized | NA |
|  |  | (*c*) If relevant, consider translating estimates of relative risk into absolute risk for a meaningful time period | NA |
| Other analyses | 17 | Report other analyses done—eg analyses of subgroups and interactions, and sensitivity analyses | Supporting Information Table S2 and Table S3 |
| Discussion | | | |
| Key results | 18 | Summarise key results with reference to study objectives | Discussion  Paragraph 1 & 2 |
| Limitations | 19 | Discuss limitations of the study, taking into account sources of potential bias or imprecision. Discuss both direction and magnitude of any potential bias | Discussion  Paragraphs 6-8 |
| Interpretation | 20 | Give a cautious overall interpretation of results considering objectives, limitations, multiplicity of analyses, results from similar studies, and other relevant evidence | Discussion  Paragraph 3 & 8 |
| Generalisability | 21 | Discuss the generalisability (external validity) of the study results | National study |
| Other information | | | |
| Funding | 22 | Give the source of funding and the role of the funders for the present study and, if applicable, for the original study on which the present article is based | Provided |
