# Supplementary material for: Femicide, intimate partner femicide, and non-intimate partner femicide in South Africa: An analysis of 3 national surveys, 1999–2017
Source: PLoS Med. 2024 Jan 18;21(1):e1004330. doi: 10.1371/journal.pmed.1004330 (PMC10796052; doi:10.1371/journal.pmed.1004330)
Supplement: S1 Text — (PDF) [file pmed.1004330.s002.pdf]

A national study of injury-related mortality, with a focus on homicide in South Africa

Ethics Application:

South African Medical Research Council Ethics Committee

Principle Investigator: Prof. Naeemah Abrahams

Acting Unit Director and Chief Specialist Scientist: Gender and Health Research Unit: South African Medical Research Council

T: 021 938 0445: F: 021 938 0310

Email: naeema.abrahams@mrc.ac.za

Co-investigator: Prof. Rachel Jewkes

Executive Scientist: Research Strategy in the Office of the President: South African Medical Research Council

T: 012 3398525: F: 021 938 0310

Email: rachel.jewkes@mrc.ac.za

Co-investigator: Prof. Lorna J. Martin

Head of Division: Forensic Medicine & Toxicology, Department of Pathology

University of Cape Town, Groote Schuur Hospital

T: 021 4066412 : F: 021 4481249

Email: Lornaj.Martin@uct.ac.za

Co-investigator: Prof. Carl Lombard

Biostatistics Unit: South African Medical Research Council

T: 021 9380328: F: 021 9380310

Email: Carl.Lombard@mrc.ac.za

Co-investigator: Prof. Shanaaz Mathews

Director: Children's Institute, University of Cape Town

T: 021 650 1473 F: 021 650 1467

Email: shanaaz.mathews@uct.ac.za

Co-investigator: Dr. Richard Matzopoulos

Deputy Director and Chief Specialist Scientist: Burden of Disease Research Unit, South Africa

T: 021 9380305: F: 021 9380310

Email: Richard.Matzopoulos@mrc.ac.za

Co-investigator: Prof. Gérard Labuschagne
Director: L&S Threat Management
Division of Forensic Medicine & Pathology
University of the Witwatersrand
T: 082 414 0527: F: 021 9380310
Email: doc@threatsa.co.za

Co-investigator: Miss. Bianca Dekel
Senior Scientist: Gender and Health Research Unit: South African Medical Research Council
T: 021 938 0838: F: 021 938 0310
Email: bianca.dekel@mrc.ac.za

Co-investigator: Miss. Megan Prinsloo
Senior Scientist: Burden of Disease Research Unit, South Africa
T: 021 938 0952: F: 021 938 0310
Email: megan.prinsloo@mrc.ac.za

Co-investigator: Miss. Bongwekazi Rapiya
Research Technologist: Gender and Health Research Unit: South African Medical Research Council
T: 021 938 0822: F: 021 938 0310
Email: bongwekazi.rapiya@mrc.ac.za

Gender and Health Research Unit and the Burden of Disease Research Unit
South African Medical Research Council
Francie van Zijl Drive
Parowvallei, Cape;
PO Box 19070
7505 Tygerberg, South Africa

Key Words
Murder, homicide, female murder/homicide, child murder, male murder, femicide, forensic pathology

Declaration

I, **Naeemah Abrahams**, have read the Department of Health: *Ethics in Health Research: Principles, Structures and Processes* (2004), the *Guidelines for Good Practice in the Conduct of Clinical Trials with Human Participants in South Africa,* Second Edition, 2006, Department of Health, Pretoria, South Africa (where applicable), and the Declaration of Helsinki (2013) and have prepared this proposal with due cognisance of its content. Furthermore, I will adhere to the principles expressed when conducting this proposed research project.

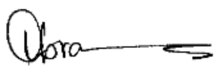

Signed:

PI: Prof Naeemah Abrahams

10 May 2018

South African Medical Research Council, Parow

Table of Contents

Declaration

Table of Contents

Definition of Terms

Executive Summary

1. Background…9

1.1. Injury-Related Mortality Data…9

1.2. Female Homicide and Intimate Femicide…9

1.3. Child Homicide…10

1.4. Male Homicide…10

2. Study Rationale…11

3. Study Aim…12

3.1. Secondary Aims…12

4. Methods…13

4.1. Study Design…13

4.2 Population and Sampling…13

4.3. Sample Size…14

4.4. Data Collection…15

4.4.1. Phase 1: FPS Mortuary Data Collection…15

4.4.2. Phase 2: Police Data Collection…17

4.5. Data Analysis…17

4.6. Data Management and Storage…18

5. Ethical Considerations…18

6. Study Outputs…19

7. Management Details…19

7.1. Project Implementation/Leadership…19

7.2. Project Manager/Co-ordinator…19

7.3. Fieldworkers…20

7.4. Co-Investigators…21

8. Time Schedule…21

9. Budget …22

9.1. Budget Justification…23

10. Short and Long-Term Consequences of the Study…27

References

Appendix A: Confidentiality Form for Fieldworkers

Appendix B: Information Sheet for Police Members Interviewed Telephonically

Appendix C: Information Sheet for Police Members Interviewed Face-to Face

Appendix D: Consent Form for Police Members

Appendix E: Part 1: FPS Homicide Mortuary Data Collection Questionnaire

Appendix F: Injury Mortality Survey

Appendix G: Part 2: Police Homicide Data Collection Questionnaire

Appendix H: Principle Investigator CV (Prof Abrahams)

Appendix I: Prof Jewkes

Appendix J: Prof. Martin

Appendix K: Prof. Lombard

Appendix L: Prof. Mathews

Appendix M: Dr. Matzopoulos

Appendix N: Prof. Labuschagne

Appendix O: Miss Dekel

Appendix P: Miss Prinsloo

Appendix Q: Miss. Rapiya

Appendix R: Checklist

Definition of Terms

- **Non-natural deaths** - include all deaths that are not due to, or may not have been due to natural causes and in terms of the Inquests Act are subject to medico-legal investigation. Non-natural deaths can be grouped by external cause of death (e.g. firearm, drowning, and burns) and apparent manner of death (e.g. homicide, unintentional, etc.).
- **Apparent manner of death -** is the terminology used in Forensic Pathology Services (FPS) to describe the intention prior to the injury that resulted in the death. Forensic services categorise unnatural deaths into five apparent manners of death:
  - Homicides,
  - Suicide,
  - Transport death,
  - Other unintentional injury death,
  - Undetermined death
- **Murder[1] -** The unlawful and intentional killing of another person.
- **Intimate Femicide** - The killing of a woman by an intimate partner. This includes the woman's husband, boyfriend (dating or co-habiting), ex-husband (divorced or separated) or boyfriend, same sex partner or a rejected would-be lover.
- **Non-Intimate Femicide** – The intentional killing of a woman by someone other than an intimate partner.
- **Child homcide** - The unlawful and intentional killing of a person 18 years and below including neonates.
- **Infanticide** – The killing of a child under 1 year of age (infant).
- **Neonaticide –** The killing of a child within the first 28 days of life (newborn).

[1] In this study, we use the term homicide in phase 1 as used by FPS. The term 'murder' will be used interchangeably with 'homicide' during Phase 2.

Executive Summary

Aim

The study has two main aims: to establish the injury-related mortality burden for South African and to establish the national incidence of homicide in South Africa. Specific aims are:

- To describe the incidence of fatal injury rates in South Africa for 2017 by age, sex and cause.
- To describe the national incidence of homicide in South Africa.
- To describe the national incidence of female homicide and intimate femicide in South Africa for 2017 and to compare this with the 1999 and 2009 studies.
- To describe the socio-demographic profile of female homicide and intimate femicide victims for 2017 and to compare this with the 1999 and 2009 studies.
- To determine whether there is a shift in the mechanism of death, in particular firearm related homicides, by describing and comparing mechanism of death of female homicide victims for 1999, 2009 and 2017.
- To describe the national incidence of child homicide in South Africa and to compare this with the 2009 study.
- To describe neonaticide and infanticide in South Africa and to compare this with the 2009 study.
- To compare incidence and circumstances of male and female child homicide in 2009 versus 2017.
- To determine what proportion of homicides (male, female, child) have a sexual component, and to compare this with the 2009 study.
- To describe the national incidence of male homicide.
- To describe the socio-demographic profile and the circumstances of male homicide.

Methods

A retrospective Forensic Pathology Services[2] (FPS) mortuary based study utilising a randomly selected, proportionate sample of FPS mortuaries will be used. This will be the same methods used in the injury-related mortality study and the previous two national female homicide studies (1999 and 2009). Data collection will be conducted through two phases: Phase 1 involves the identification of injury-related cases from death registers from 1 January 2017 until 31 December 2017 at sampled FPS mortuaries and extraction of data from the FPS mortuary files. Phase 2 will focus on the identified homicide cases and will involve follow-up interviews with the investigating officers (South African Police Service (SAPS) members) to collect victim, alleged perpetrator, police and justice data to establish victim-perpetrator relationship and to obtain information about the crime.

[2] All Forensic Pathology Services FPS mortuaries or facilities in South Africa as per the National Health Act (61 of 2003) and the Regulations Regarding the Rendering of Forensic Pathology Service (No. 341, 2005).

Outcomes

The study will provide a wide range of outcomes. Trend data for injury-related mortality will feed into the National Burden of Disease Study. Similarly, trend data for intimate femicide and child homicide in South Africa will assist in providing a comprehensive overview of these forms of violence against women and children. The study will for the first time also provide detailed information on male homicides. Since this study will constitute the third femicide study, the findings will allow us to assess the extent to which policy shifts and service provisions have increased women and children's safety.

Intended Feedback and Research Translation

We will use various platforms for research dissemination. The results will be written up and published in peer-reviewed journals and research briefs will be distributed widely with all stakeholders including advocacy groups. Report back meetings will also be held with the Department of Health (DOH), SAPS, Department of Correctional Services and the Department of Justice to feedback results and recommendations for each section. The findings will also be presented at local and international conferences. The previous injury-mortality study and the female and child homicide study have published more than 12 peer-reviewed journal articles as well as three research briefs and a report that informed the National Burden of Disease Study. The 2009 femicide research findings (i.e. three women killed by a partner per day/one woman killed by a partner every eight hours) is one of the most common cited statistics to refer to when addressing violence against women in South Africa by media, activists and policy makers. Robust data on violence can provide invaluable information towards monitoring the two key Sustainable Development Goals (SDGs) i.e. Goal 5 (achievement of gender equality) (Garcia-Moreno & Amin, 2016) and Goal 16 (peace, justice and strong institutions) (Matzopoulos & Bowman, 2016).

1. Background

South Africa has one of the highest injury burdens in the world. This is largely due to the unusually high rates of homicide and road traffic injury mortality, which have been placed second and fourth amongst the leading causes of premature mortality (Msemburi et al., 2014). In 2009 the Injury Mortality Survey (IMS) estimated 52 493 injury-related deaths (mortality rate for all injuries was 109 per 100, 000 population); of which nearly half (48.6%; 25 499/52 493) were intentionally inflicted. Homicide was the leading apparent manner of death, accounting for 36.2% (19 028/52 493) of all external causes or 38.4 per 100, 000 population (Matzopoulos et al., 2015). This rate is also significantly higher than the 2012 global homicide rate (6.2 per 100,000) (UNODC, 2013).

1.1 Injury-Related Mortality Data

The absence of reliable surveillance data and known deficiency in vital registrations creates challenges to access data on injury-related mortality. The IMS has shown the value of a dedicated study and abstracting data from post mortem reports. The first comprehensive profile of injury mortality in 2009 provided cause specific rates and contributed towards a better understanding of the high underreporting of homicides and road traffic deaths. Such data is vital to model and validate cause of specific burden of disease trends, such as the road traffic injury mortality rate, which remained consistent between 2000 and 2009 and the female suicide rate, which decreased significantly during this period (Matzopoulos et al., 2015).

1.2 Female Homicide and Intimate Femicide

Two national female homicide studies have been conducted providing a comparison between 1999 and 2009 data. A female homicide rate from 24.7 per 100, 000 population in 1999 to 12.9 per 100, 000 population provided evidence of a decline over ten years (Abrahams, Mathews, Martin, Lombard, & Jewkes, 2013). A similar decline was observed in the IMS for the same year (2009) (Matzopoulos et al., 2015). However, the rate of 12.9 per 100, 000 population remains higher than the global female homicide rate (2.7 per 100,000 females) (UNODC, 2013). The decrease of 37.7% (i.e. 3793 deaths in 1999 to 2363 deaths in 2009), reflects 1430 fewer female homicides (defined as homicides of women 14 years and older) over the ten years (Abrahams et al., 2013).

The primary aim of the first female homicide study was to describe intimate femicide and the study found that half (50.3%) of the female homicides were committed by an intimate partner in 1999. Despite the observed decrease in the population rate of intimate femicide (8.8 per 100,000 vs 5.6 per 100, 000) 10 years later in 2009, the proportion of intimate femicide among all female homicides increased to 57% (Abrahams, Mathews, Jewkes, Martin, & Lombard, 2012). The 2009 rate was still more than double the United States rate (2.0 per 100,000 population) (Logan, Smith, & Stevens, 2011). Intimate femicide is the leading cause of female homicide in South Africa. It is well established that femicide is rarely the first act of violence against a woman and thus, it is crucial that this work is

prioritised so that those who kill women are held responsible and appropriately punished (Abrahams et al., 2012).

1.3 Child Homicide

Violence against children is a common feature in South Africa (Burton, Ward, Artz, & Leoschut, 2015). The addition of child homicides to the second female homicide study in 2009 provided the first opportunity to explore child homicides in South Africa in detail (Mathews, Abrahams, Jewkes, Martin & Lombard, 2013). The study found 1018 child homicides occurred in 2009, providing a rate of 5.5 homicides per 100, 000 children under the age of 18 years. This rate was double the World Health Organisation's (WHO) estimated global rate of 2.4 per 100, 000 (Pinheiro, 2006) and reflects children's vulnerable position in South African society. Almost half (44.5%) of all the child homicides were identified as related to child abuse and neglect (Mathews et al., 2013). Violence against children studies have shown that by the time children in South Africa are 15–17 years old, many would have experienced abuse, neglect and maltreatment (e.g. Burton et al., 2015). For example, the findings from the Optimus Study found that between 16.8% and 35.4% report experiences of some form of sexual abuse; 20.8–34.8% of children report experiences of physical violence, emotional abuse ranges between 16.1% and 26.1% and 12.2% and 15.1% experiences of neglect (Burton et al., 2015). These forms of abuse have serious consequences both for the children who suffer them and for national development, as they are more likely to develop long-term physical and mental issues, which in turn, undermine their capacity to succeed in life.

The analysis of the child homicide data from the second female homicide study showed distinct age and sex patterns. Overall, the homicide rate was much higher in boys (6.9 per 100, 000) than in girls (3.9 per 100, 000). In contrast, amongst young children under the age of five, females had the highest homicide rate (8.3 per 100,000), which likely reflects the lower social value assigned to girls (Mathews et al., 2013). The study found 454 children under the age of five were killed in 2009. More than half (53.2%) were neonates (0–28 days old), and 74.4% were infants (under 1 year of age), giving a neonaticide rate of 19.6 per 100,000 live births and an infanticide rate of 28.4 per 100,000 live births (Abrahams, Mathews, Martin, Lombard, Nannan, & Jewkes, 2016). These rates are amongst the highest reported rates for neonaticide and infanticide, surpassed only by the estimate for Dar es Salaam (27.7 per 100,000 live births) (Outwater, Mgaya, Campbell, Becker, Kinabo, & Menick, 2010).

1.4 Male Homicide

The high rate of homicide in South Africa is largely driven by male homicides (67.4 per 100, 000 male population) (Matzopoulos et al., 2015) and the prevailing feature is the disproportionate role of young men as perpetrators and victims. Dominant masculinity ideals ensure that demonstrations of toughness, bravery, and defence of honour, translate into the ready use of violence, while a higher status is gained by fighting as opposed to resolving conflicts peacefully (Seedat, Van Niekerk, Jewkes, Suffla, & Ratele, 2009).

South Africa's male homicide rate (67.4 per 100, 000 male population) in 2009 was significantly higher than the global male homicide rate (9.7 per 100,000) (UNODC, 2013). The IMS found a male-to-female ratio of six males per female with men mainly killed as a result of sharp force/stabbing (28.1 per 100,000) (Matzopoulos et al., 2015). Despite male homicides constituting a huge injury burden, research on the profile of victims and perpetrators of male homicide has not been done, other than the work done by IMS (Matzopoulos et al., 2015). It is critical to understand the epidemiology, including data on the crime such as victim-perpetrator relationships, perpetrator data, alcohol relatedness of the crime, mechanisms – particular firearm related deaths, and the justice outcomes. Some of this information is available from administrative data i.e. police annual reports on homicide, but the various aspects of the homicide cases are not linked between the departments and a full understanding cannot be gained (i.e. linked data between the forensic pathology service, SAPS, the prosecution services and justice). The inclusion of male homicides in this national study will provide the link between departmental data for the first time. The value of linked data was shown for both women and children in the previous studies and provided a valuable opportunity to engage in-depth with these extreme forms of violence. These previous studies also proved invaluable for service providers and policy makers (police, justice, health, and social development) to understand the extent of the problem. We require the same for male homicides – particularly, as it is the most vulnerable group for homicides in the country.

2. Study Rationale

This study will provide multiple opportunities to better understand injury burden and more specifically interpersonal violence trends in the country. Updated injury data are not readily available from the South African administrative systems, while the analysis of trend data is critical for monitoring the disease burden and interpersonal violence.

The study will contribute hugely to building national data on homicide in South Africa. Although the 2009 femicide study showed a decrease in overall female homicide and an overall decrease in intimate femicides, this decrease was not statistically significant. This may be an indication that the impact of interventions or national efforts to prevent gender-based violence were limited. If these efforts had been effective, a larger reduction in intimate femicide than in non-intimate femicide would have been noted (Abrahams et al., 2012). Doing a third study will provide a third data point to establish a definite trend over 18 years. This third study could also be considered a monitoring tool for violence against women.

Furthermore, the 1999 study highlighted that in the majority of cases of intimate femicide, SAPS dockets had not recorded past history of Intimate Partner Violence (IPV), despite research indicating that this is very common and valuable in securing a conviction. This is also of concern since it is seldom that the first act of violence against an intimate partner is fatal. Having highlighted the failure to enquire about prior violence after the 1999 study, the researchers had hoped to see this recorded more frequently. However, in 2009 they found no difference in the identification of prior IPV during the

investigation of the intimate femicide cases (Abrahams et al., 2012). Thus, a follow up into the investigation of prior IPV is needed.

In addition, the 2009 data showed a decrease in convictions of perpetrators (Abrahams et al., 2012). Further research is needed to determine whether there has been an improvement in conviction rates. Our study will cover 2017 and we plan to collect police data from April 2019 until the end of September 2020.

In South Africa, children experience high rates of abuse, maltreatment and neglect. The need for child protection services far outweighs the ability of existing services to respond (Mathews et al., 2013). Understanding the epidemiology of child abuse that culminates in death is imperative for developing and monitoring interventions to prevent it. To explore this problem in South Africa, a second national child homicide study is needed as monitoring such violence provides insights on the impact of laws, policies and programs for violence against children prevention. This study will provide us with data to compare with the patterns identified in the previous study. In addition, the Child Death Review (CDR) project conducted by the Children's Institute in two provinces (Western Cape and Kwa Zulu Natal) identified gaps in service provision (Mathews et al., 2016) and this study will allow us to compare this with national patterns. Such data is critical in the development of interventions to influence protection of children. This will allow us to understand whether the policy shifts and services provision have increased children's' safety in the home.

Finally, an in-depth investigation into male homicide has not yet been undertaken in South Africa. This study will provide the first detailed and comprehensive national study of male homicide in South Africa. Although the IMS provided a male homicide rate and provided information surrounding manner of death (Matzopoulos et al., 2015), this study will provide crucial data on further aspects related to the crimes, such as victim and perpetrator socio-demographic characteristics, victim-perpetrator relationship and crime related data.

3. Study Aim

The study has two main aims: The one is to establish the incidence of homicide in South Africa with a focus on male, female, intimate femicide, and child homicide for the year 2017. The second aim is to determine the injury burden.

3.1. Secondary Aims

3.1.1. To describe the incidence of fatal injury rates in South Africa for 2017 by age, sex and cause.

3.1.2. To describe the national incidence of homicide in South Africa.

3.1.3. To describe the national incidence of female homicide and intimate femicide in South Africa for 2017 and to compare this with the 1999 and 2009 studies.

3.1.4. To describe the socio-demographic profile of female homicide and intimate femicide victims for 2017 and to compare this with the 1999 and 2009 studies.

3.1.5. To determine whether there is a shift in the mechanism of death, in particular firearm related homicides, by describing and comparing mechanism of death of female homicide victims for 1999, 2009 and 2017.

3.1.6. To describe the national incidence of child homicide in South Africa and to compare this with the 2009 study.

3.1.7. To describe neonaticide and infanticide in South Africa and to compare this with the 2009 study.

3.1.8. To compare incidence and circumstances of male and female child homicide in 2009 versus 2017.

3.1.9. To determine what proportion of homicides (male, female, child) have a sexual component, and to compare this with the 2009 study.

3.1.10. To describe the national incidence of male homicide.

3.1.11. To describe the socio-demographic profile and the circumstances of male homicide.

4. Methods

4.1 Study Design

The study is designed as a retrospective national mortuary based study, utilising routine data collected through death registers to identify all cases of injury-related deaths and more specifically for male, female and child homicides for 2017 at sampled FPS mortuaries in South Africa. All homicide cases will be followed up with the investigating officer (police detective/member) to collect data recorded in the police docket on both the victim and perpetrator, to establish victim-perpetrator relationship and to obtain information about the crime.

4.2 Population and Sampling

The study population comprises all unnatural deaths (injury-related deaths) during the year 2017 (i.e. all cases that were admitted to a FPS mortuary). All suspected unnatural deaths in South Africa are admitted to the FPS for the medico-legal investigation of that death and all suspected homicides fall into the definition of an unnatural death, according to the regulations rendering forensic pathology services in South Africa. The sampling frame will be the list of operating FPS mortuaries in South Africa for the year 2017 and the number of autopsies done in each. This will be compiled once a list of all the operating FPS mortuaries and bodies has been obtained from the DOH. This information has been promised after ethical approval of the study is obtained.

The sampling frame will be prepared for sampling with mortuaries as the primary sampling unit. The identified FPS mortuaries will be stratified into three strata based on the number of admissions at each FPS mortuary for 2017: under 500 bodies (strata 1), 500 to 1499 bodies (strata 2), and 1500 or more bodies (strata 3) and by metro and non-metro. We are not able to provide the detail of the sample as the complete sampling frame is not available. However, it is unlikely that FPS services have changed

in the last eight years (since the 2009 studies) and thus, we expect to have a similar sampling frame used by both the IMS and the female homicide study. The sampling frame in 2009 was 57 274 post mortem reports from 106 mortuaries. As before, the final samples will be based on a weighted proportionate random sample of each stratum, where each strata of FPS mortuaries are considered to be a cluster. The date of admission will be taken as date for sampling. The sample will allow only for national estimates and not for provincial estimates. Doing provincial estimates will require a bigger sample and this will require more resources to do data collection.

4.3 Sample Size

We expect to draw a larger sample size than the sample of 45 (45/109) mortuaries, which was the figure for the 2009 IMS. This is because the 45 mortuaries were drawn from eight provinces and all data for the Western Cape was provided from the Provincial Injury Mortality Systems. We do expect to receive the IMS data for Western Cape again, however, a sample of mortuaries will still be drawn for the female and child homicide part of the study in the Western Cape. We will also take into account that the authors of the IMS study reported they may have under-sampled the Mpumalanga province. We therefore, estimate the sample will be in the region of 50-52 mortuaries and we used this estimate to develop the fieldwork plan and budget. This larger sample will ensure adequate power to meet both the injury mortality study aim and which; will allow for sub-analysis of the intimate femicide and child homicide data. To note: women aged 14-18 years will be considered for two analyses: that of female homicides and that of child homicides. As with the 2009 studies, we want to ensure that the sample is representative and therefore, we will include both small, rural FPS mortuaries and large ones attached to medical schools. The sampling frame will also be stratified into metro and rural mortuaries. The IMS has shown the value in this stratification as injury-related deaths linked to motor vehicles accidents showed higher pattern in Gauteng only. We are confident that the larger sample of mortuaries will be adequate as the IMS validated and found a similar female homicide rate as the female homicide study (11.3 per 100, 000 vs 12.9 per 100,000) (Abrahams et al., 2013; Matzopoulos et al., 2015).

We therefore, expect the sample size to be adequate to conduct a comparison of intimate femicides for the three study years: 1999, 2009, and 2017, with 90% power and at 5% significance. Collecting all homicides in South Africa will allow us to do a sub-population analysis. We would be able to analyse all female homicides from the age of 14 years as we did for the 1999 and 2009 study and this will also enable us to do an intimate femicide analysis. We will be able to look at all child homicides i.e. all children under the age of 18 years, as we did for the 2009 study and to do a comparison. Lastly, we will also do a sub-analysis of all male homicides (children and adults) which will comprise a first detailed analysis of male homicides in the country.

We will collect and abstract data on all the child and female non-natural deaths in all sampled mortuaries. Since males comprise a huge number of cases across the injury profile, we will extract data on all males in the small and medium strata mortuaries and every second male case in the strata 3

mortuaries (more than 1500 bodies per year). Finally, our sampling frame will be used to draw a nationally representative sample of mortuaries stratified by metro and non-metro areas with provincial level selection based on mortuary location.

4.4 Data Collection

The study's data collection will be conducted through two phases as outlined below:

4.4.1. Phase 1: FPS Mortuary Data Collection

The first phase of data collection will entail fieldwork at the selected mortuaries. This phase is also divided into two processes. The first process will largely follow what was done in the IMS. All cases recorded in the register from 0:00hrs on 1.1.2017 to 23.59hrs on the 31.12.2017 will be assessed to determine if it was a natural, non-natural or undetermined cause of death. All non-natural deaths and undetermined deaths will be identified for further abstraction and clearly marked natural deaths will be excluded for further extraction and only a head count of all the natural death cases per mortuary will be done (see figure 1). As described above: at the large mortuaries every second male case (non-natural) will be included for further extraction and head counts of those excluded, including the apparent manner of death (homicide, suicide, transport related, other unintentional or undetermined intent) will be recorded from this first line of data extraction from the mortuary register. Inter-observer reliability will be tested independently by two fieldworkers, who will collect data from the same folder, for 10% of the sample. Every 20th folder will be reserved for independent capture by another fieldworker.

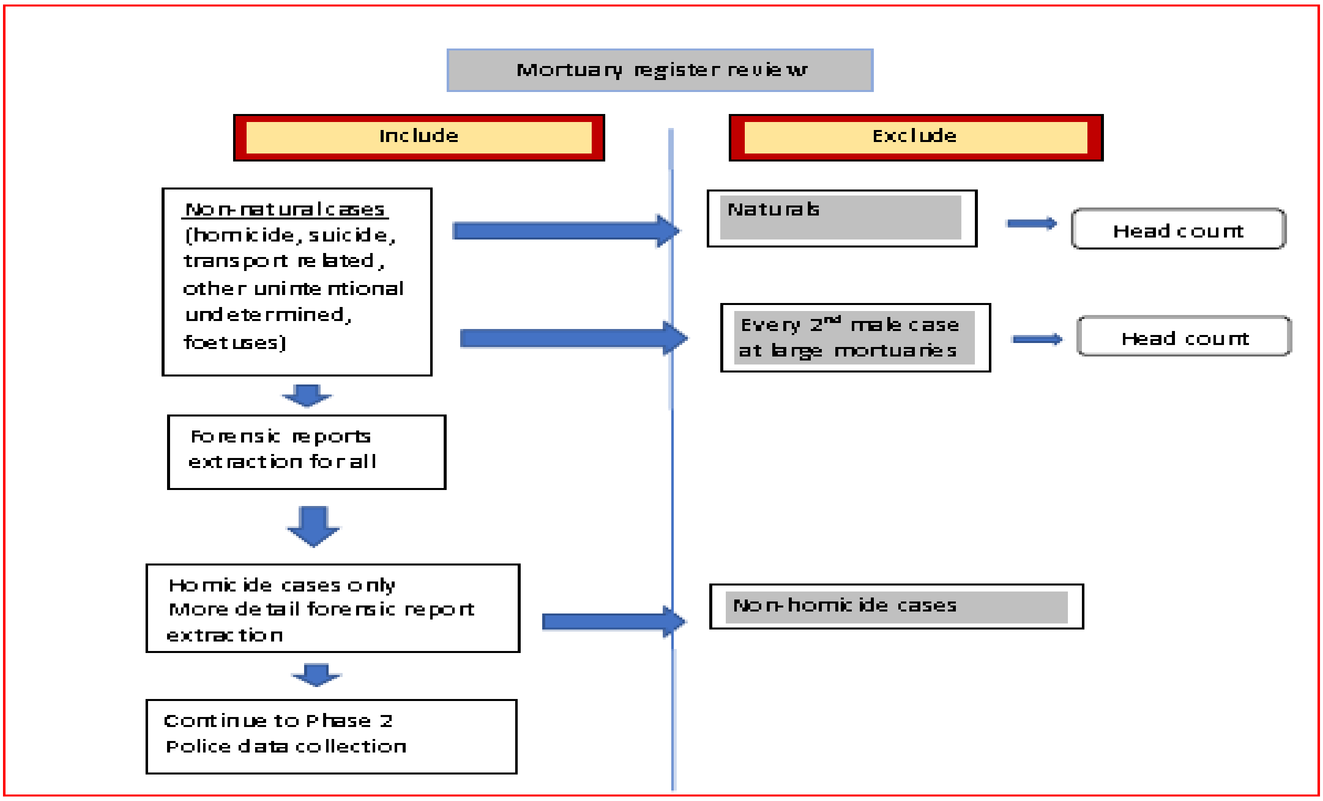

Figure 1 Flow of data extraction in sampled mortuaries

The second process will be the further abstraction of data on the identified non-natural deaths/undetermined cases from the mortuary files. The manner of death categories used will be similar to those used in the 2009 IMS corresponding to the International Classification of Disease (10th edition) (see Table 1 below). Alcohol data on all these cases will be collected if available in the file. Additional data will be extracted for the homicide cases and these cases will progress to the 2nd phase of data collection (i.e. police data collection) (see below).

Data will be collected on tablets, through using REDCap (Research Electronic Data Capture), which is designed to collect and manage data for research studies and is a secure web application for building, and managing online surveys and databases. REDCap has been used and proven beneficial in a number of studies of similar size (e.g. Christopoulos et al., 2014; Divo et al., 2012; Green-Simms, Ekdawi, & Bakri, 2011). Through using REDCap, a standardised data collection questionnaire, based on the IMS and female homicide study will be used (Appendix E and F). This questionnaire has been adjusted based on the experience of the researchers – in particular related to abandoned babies and criminal justice studies done on rape. The information to be collected for the homicide cases from the FPS mortuary records will include: police case number, police station, social and demographic characteristics of the victim (e.g. age), estimated date and time of death, blood alcohol levels if taken, Sexual Assault Examination Collection Kit (SAECK) used, indication of a sexual assault/rape reported, indication of pregnancy, mechanism of death and multiple nature of injuries. For neonates and babies', we will collect measurement data such as weight, height, estimated gestation and evidence of viability, the latter to identify 'concealed pregnancies' (a crime in South Africa) and to differentiate from stillborn.

Table 1: Categories to be extracted and coded corresponding to the ICD 10

Cause of injury	ICD-10 code
Homicide	X85–X99, Y00–Y09
Suicide	X60–X84
Transport injuries	V00–V99
Road traffic injuries	V00–V04,V06, V09–V80, V82–V85, V87, V89
Other transport injuries	V05, V81, V86, V88, V90–V99
Poisonings	X40–X49, X67–X69
Falls	W00–W19
Fires, heat and hot substances	X00–X19
Drowning	V90, V92, W65–W70, W73, W74
Mining accidents	W77, Y37
Other threats to breathing	W75–W84
Mechanical forces	W24–W34, W45–W46
Exposure to natural forces	X30–X39
Adverse effects of medical and surgical treatment	Y39–Y66, Y68–Y84, Y88
Animal contact	W53–W59, X20–X27, X29
Other unintentional injuries	W20–W23, W35–W44, W49–W52, W60, W64, W85–W94, W99, X28, X50–X59, Y38
Unspecified or not listed	Y09, Y10-Y34, Y36, Y85-Y87, Y89

Source: World Health Organization (2016).

4.4.2. Phase 2: Police Data Collection

The police data collection will only include the homicide cases in the second phase and will comprise the collection of data from police members/detectives which, is usually information drawn from the docket. We will attempt to begin this phase of data collection during the middle of 2019 with the hope that some of the early cases would have progressed through the investigation process. However, we plan on ending this phase of data collection towards the end of September 2020, so this gives us 1 year and 5 months to collect police data. We have given thought to this period and if we choose an earlier period, there is a concern that the mortuary registers might not be available. The data collection in this phase will focus on the victim, alleged perpetrator and the crime. An interview with the primary investigating officer will be sought. We know cases are moved between police detectives and we will request to interview the member who knows the case best. If this is not possible, a secondary police source or a record review will be used. Telephonic interviews will be done when face-to-face interviews are not possible. Information collected from police will include, for example, social and demographic characteristics of the alleged perpetrator, circumstances surrounding the killing, victim-perpetrator relationship, and history of IPV (in cases of intimate relationships). The Rape Adjudication and Prosecution Study in South Africa (RAPSSA) provided us with much learnings on data collection on crime and legal case progression and we have developed questions related to this (Appendix G). The type of homicide for females (intimate femicide/non-intimate femicide) and children (abuse or non-abuse) will be coded once the police interview has been completed. As for the previous studies, we expect to have cases where there is no suspect and these will be coded as 'perpetrator unknown'.

We do not expect to find blood alcohol levels to be collected as a routine measure in child victims below five years. As with the previous study, we will add questions that pertain to children. The exact age in day/months would be important for neonatal deaths, details on the primary caregiver, living arrangements and if the child was ever referred or known by social workers or other child support workers.

Lastly, the questionnaire has been prepared in English as our experience in the previous studies has shown that police were all able to conduct interviews in English.

4.5 Data Analysis

Data will be analysed using Stata version 15.0. We will take into account the survey design, including the different sampling weights taking into account the selection probabilities of mortuaries in the survey strata. We will use standard methods for the analysis of data from a sample survey estimate for the estimates such as number and proportions of deaths (incidence rates) and the mechanisms of death will be calculated. Pearson's chi-square test will be used to determine significant differences between groups (e.g., those murdered from IPV and those murdered non-intimate partners). Mortality rates will be calculated (male, female and children) overall and presented for age-groups and race

groups (to create a socio-demographic profile), where the denominator source will be the South African 2011 national census. These population estimates will be adjusted for annual growth using procedures commonly used in mortality studies. Descriptive statistics (survey means and proportions) will be calculated, as well as standard errors and 95% confidence intervals. Categorical variables will be compared using Pearson's $\chi 2$ test, and standard errors and 95% confidence intervals will be calculated using methods for complex sample surveys (Taylor linearization). We will use a regression analysis to test differences between means for continuous variables and logistic regression to test differences between proportions for categorical variables. Finally, we will use domain analysis to allow us to analyze the subpopulations of interest, e.g. adult females, children (0-17 yrs), children <5 years, abandoned neonates, adult males, adolescent males etc.

4.6. Data Management and Storage

Data collected through REDCap will be securely stored on South African Medical Research Council (SAMRC), password protected computers, which are the property of the Gender and Health Research Unit (GHRU), and which, complies with the SAMRC IT policy for electronic storage of data. All data will be stored under the authority of Prof. Abrahams, for at least 15 years.

5. Ethical Considerations

Access to FPS mortuaries will be gained through the DOH and through each provincial head responsible for FPS mortuaries. The person in charge of each FPS mortuary will also be approached to access death registers and post mortem reports at the sampled FPS mortuaries. The study will be conducted anonymously as questionnaires will not collect the name of the victim or perpetrator. Cases will only be identified by the unique research number assigned to them. As study subjects are deceased, records are reviewed at FPS mortuaries and cases are followed-up with investigating officers and thus, informed consent is not necessary. However, the confidentiality of victims and perpetrators will be ensured at all times and all fieldworkers will sign confidentiality forms (Appendix A).

Permission to access information from investigating officers will be obtained via the office of the National Commissioner of Police and the research department. Similarly, we will seek permission from the relevant Research Committees within the DOH at national and provincial level. We will provide them with an information sheet (Appendix B and C) and written informed consent will be sought from each investigating officer prior to the telephonic or face to face interview (Appendix D). Regarding the interviews with officers done telephonically: information and consent forms will be emailed to the officers and only once we receive the completed form will we proceed to make telephonic contact. Regarding face to face interviews done with officers: we will provide these forms in person before conducting the interview. Investigating officers will be reassured that all data collected is confidential and anonymous. The researchers believe that one has an ethical responsibility toward the deceased. Where it is found that their deaths have not been investigated, the researcher will ensure that such instances will have a case opened by working with SAPS.

We will start with the collection of the pathology data at the mortuaries in October 2018 and will only commence data collection from police in 2019 to ensure 2017 cases have progressed through the investigation and justice systems.

6. Study Outputs

The three previous study findings were used extensively as an advocacy tool to place the issue of intimate femicide on the agenda of both civil society as well as policy makers nationally and globally. Locally, this was achieved through a partnership with the Commission on Gender Equality as well as using the study findings to lobby for stricter firearm control with submissions to parliament for the Firearms Control Act. It is anticipated that the findings from this study will be used in a similar manner to further influence policy development in the area of intimate femicide through the dissemination of findings at both a national and international level. Similarly, the study will provide a second national incidence of child murders. All findings will be distributed and shared with all stakeholders. The findings will be published in peer-reviewed journals and presented at conferences. The findings will form the basis for recommendations on the surveillance and prevention of male murder, intimate femicide and child murders by targeting service providers and decision makers.

7. Management Details

7.1. Project Implementation/Leadership

Prof. Naeemah Abrahams (PI) will oversee the overall implementation of the study and will provide leadership to the project. She was involved in the previous three studies (1999 and 2009 femicide studies as well as the IMS) and has extensive experience in overseeing a study of this nature. She will also be responsible for obtaining the necessary approval from the DOH and Ministry for Police to be able to conduct the study. She will liaise with the necessary heads of provincial health departments to gain access to the sampled FPS mortuaries. She will lead the analysis and the interpretation of the data as well as the scientific writing up of the study. She will be supported by Dr. Matzopoulos from the Burden of Disease (BOD) Unit who was the PI on the IMS.

7.2. Project Manager/Co-ordinator

Miss. Bianca Dekel will be responsible for the overall co-ordination of the study and for setting up the fieldwork. Miss. Bongwekazi Rapiya will assist Miss. Dekel with the planning of the fieldwork. Miss. Dekel and Miss. Rapiya are both working in the GHRU and both have experience in working within the criminal justice system. Miss. Dekel is currently completing her PhD on child homicide perpetrators and Miss. Rapiya is a Research Technologist who has been with the GHRU since 2002 and has had an array of fieldwork experiences, including the 2009 Femicide Study.

Miss. Dekel will also be responsible for liaising with co-investigators in developing the protocol, for scientific advice, and for getting the protocol through the Ethics application. She will assist in the

analysis and the interpretation of the data as well as the scientific writing up of the study. Miss. Megan Prinsloo from the BOD Unit will provide support as she coordinated the IMS.

7.3. Fieldworkers

Miss. Dekel and Miss. Rapiya will be responsible for organising the fieldwork and for conducting some of the data collection. They will be supported by approximately 20 fieldworkers in phase 1. Matriculants with fieldwork experience will be recruited from the nine provinces for data collection at the mortuaries. A competency test will be developed to ensure that the appointed fieldworkers are well-organized and logical thinkers who will have the necessary skill and familiarity to capture information quickly on tablets. The competency test will also inform the suitability of the fieldworkers to fulfil a "team-leader" function to liaise and provide progress reports to Miss. Rapiya. In phase 2 more senior level fieldworkers will be employed to do interviews with police.

The training of fieldworkers will take place in one location with all provinces represented to build a sense of the importance of this research for the entire country and to enhance team cohesion. Fieldworkers will be bussed in from differing provinces. Fieldworkers will be trained on the purpose and importance of the study, sampling strategy, research ethics, post mortem investigation procedures, structure of post mortem folders, the data capture instrument, data capture using tablets, their roles and responsibilities in the field, the project organisational structure, logistics of the project, the reporting structure within the project team, the SAMRC's values, SAMRC procedures for grievance/injury on duty/accidents and reasons for disciplinary action or termination of their contracts. A practical exercise will be incorporated into the training whereby the fieldworker will be required to capture data from post mortem report scenario's created by injury mortality experts/forensic pathologists. Each fieldworker will be required to successfully complete a competency assessment before they are contracted as a fieldworker for the study. Miss. Dekel and Miss. Rapiya will develop an extensive and in-depth fieldwork manual to train fieldworkers prior to data collection. This manual will be provided to each fieldworker after they successfully complete the training. The manual will provide information on all topics discussed in the training and include contact details for the project team and counselling support services.

The electronic data collection tool, data management and quality control software will be piloted. This piloting will form part of an intensive orientation and training programme that will also include the review and finessing of training materials prior to their use for fieldworker training.

The aim is to have fieldworkers recruited from each province, working in pairs. Miss. Dekel and Miss. Rapiya will be present and will assist with data collection in four of the nine provinces where large mortuaries are located (and very likely to be included in the sample): the Western Cape, Gauteng, Eastern Cape and Kwa-Zulu Natal. Data from these provinces will be collected first so as to ensure that all fieldworkers are well-equipped when collecting data from the remaining provinces.

Overall, fieldwork pairs/teams will be required to:

- Find[3] the selected mortuaries within their province;
- Gain access to post mortem registers and folders by negotiating a working agreement with the mortuary managers and administrators (once permission has been obtained by the project management team);
- Capture required data from post mortem folders;
- Each fieldworkers to reserve every 20th folder so that it can be captured by a different fieldworker on the same day;
- Report access challenges or any other issues to the coordinator; and
- Attend group debriefing at the end of fieldwork.

Miss. Dekel and Miss. Rapiya will set up a helpline that will be available to all fieldworkers during the daily course of data collection if they require assistance or if they need help dealing with any potential challenges that may arise. All cases will be identified and data collected under the guidance of Prof. Abrahams, Prof. Lorna Martin and Dr. Richard Matzopoulos. Quality control measures will be developed with monitoring of data collection and data entry processes in the field.

7.4. Co-Investigators

The core team that led the previous female homicide studies include: Prof. Jewkes, Prof. Martin, Prof. Mathews, Prof. Lombard and Prof. Abrahams and they will continue to provide scientific advice on the study design and analysis and will also assist with the writing up of the study. Most of the core team was also part of the IMS where they joined the team of Dr. Richard Matzopoulos. Prof. Lombard will continue to assist in the selection of the sample and weighting of sample as he has done for the three previous studies. He will also advise and assist with the statistical analysis, as he did in the previous studies. Prof. Martin will provide advice regarding the Forensic Pathology Service and the forensic pathology aspects of the cases. Prof. Labuschagne joins the team on this study and will provide advice regarding the overall criminal justice process and surrounding the collection of police data. Lastly, although the study is housed within the GHRU in Cape Town, it is a collaboration between GHRU and the BOD Unit. Most of the primary operational support will be provided by the GHRU.

8. Time Schedule

	April 2018 – Sep 2018	Oct 2018 - March 2019	April 2019 - Sep 2019	Oct 2019 - March 2020	April 2020 - Sep 2020	Oct 2020 - March 2021
**Gain access**<br>**Prepare sample**<br>**Prepare research tools**<br>**Plan data collection**<br>**Training of**	xxxxxxxxxxx					

[3] Miss. Dekel and Miss. Rapiya will provide fieldworkers with details as to where each mortuary is located – however, fieldworkers need to be able to get to the mortuaries for work.

**fieldworkers**						
**Data collection:**<br>**Phase 1: FPS Mortuary data collection**		xxxxxxxxxxx				
**Data collection:**<br>**Phase 2: Police data collection**			xxxxxxxxxxx	xxxxxxxxxxx	xxxxxxxxxxx	
**Data analysis**<br>**& Report writing**					xxxxxxxxxxx	xxxxxxxxxxx
**Dissemination of Findings**						xxxxxxxxxxx

9. Budget

	**Year 1: Phase 1 (Mortuary data collection)**	**Year 2: Phase 2 (Police data collection)**	**Year 3: Data analysis, report writing & dissemination of findings**
**Salaries**:			
Bianca	R588 500	R732 222	R783 477
Bongwekazi	R280 000	R299 600	R320 572
Research Assistants	R165 600	R399 364	/
Administrator	R160 500	R80 250	R80 250
Consulting Psychologist	R80 000	/	/
**Local Travel & Accommodation for Training & Data Collection**:			
Flights	R140 600	R79 180	/
Accommodation	R186 000	R51 360	/
Per diem	R44 450	R9200	/
Car hire and petrol	R143 988	R44 600	/
Shuttle services	R34 200	R18 000	/
**Equipment**:			

20 Tablets for 20 data collectors	R50 000	/	/
**Office Supplies**:			
E.g. Paper, ink cartridges, stationary	R12 000	R12 000	R6000
**Other**:			
Mobile data for tablets for data collectors	R8640	R3840	/
Monthly cell phone data allowance for fieldworkers	R9600	R13 200	/
Monthly cell phone data allowance for Bianca & Bongwekazi	R13 200	R13 200	R13 200
Courier	R2750	/	/
Laptop needed for Bongwekazi and Bianca	R22 000	R22 000	/
**Investigator Meeting:**	R16 038	R16 038	R36 535
**Dissemination of findings:**	/	/	R150 000
**Feedback meeting with stakeholders in Pretoria:**	/	/	R30 423
**TOTAL COSTS:**	**R1 953 066**	**R1 794 054**	**R1 420 457**

9.1. Budget Justification:

Salaries:

Co-investigators:

Although co-investigators will provide scientific and technical expertise to the study, they will not draw a salary from this project. Most of the senior staff are full time employees of SAMRC and since we will be applying to SAMRC for funding for this study, we are not allowed to request salaries for senior staff. However, this project will cover Miss. Dekel, Miss. Rapiya and the administrator’s salary

(who will be paid according to SAMRC policies). Additionally, Miss. Dekel's year 2 and 3 salary will differ from her year 1 salary, as she would have graduated and will be promoted to a higher level.

Project administration:

A full time administrator will be employed for the first six months of phase 1 when the data collection process will be administratively intensive i.e. travel bookings, procurement and financial management. Thereafter the current Administrator of the GHRU for the Cape Town Branch will dedicate 20% of her time to assisting with further administration until the completion of the project.

Consulting Psychologist:

A consulting psychologist will be employed on a part-time basis to provide support to fieldworkers. He/she will be employed for a total of 200 hours (i.e., 8 hours per week x 25 days (one month) = 200 hours), at a rate of approximately R200 per hour, which will be spread out over the period of data collection.

Fieldworkers:

All 20 fieldworkers will be paid per SAMRC salaries based on qualifications (the approximate assistant rate for year 1 is R75 p/h and R81 p/h for year 2).

Phase 1:

Each fieldworker will have two days of training. We know from previous experience that the larger mortuaries (based within Western Cape, Gauteng, Eastern Cape and Kwa-Zulu Natal) should take approximately 15 days for phase 1 data collection to be complete. The smaller mortuaries within the remaining provinces should take approximately 5 days for phase 1 data collection to be complete. Therefore, assistants in different provinces will be paid differing amounts. For phase 1's data collection from the larger provinces: it will entail 15 days of work plus 2 days of training = 17 days in total (@R75 p/h) = R10 200 per fieldworker. The smaller mortuaries will entail 5 days of work plus 2 days of training = 7 days in total (@R75 p/h) = R4200 per fieldworker.

Phase 2:

The second phase of study requires more complex interviews and two research assistants will be hired from the GHRU's Rape Impact Cohort Evaluation Study (RICE) that is due to end in mid-2019. Eligible staff will be drawn from a group of fieldworkers already trained in research done in the GHRU. Their salary will be approximately R199 682 (per annum as phase 2 data collection is 12 months) each according to their qualifications and prior experience.

<u>Local Travel and Accommodation</u>:

Phase 1:

Flights: Approximately 38 flights needed for training and data collection at R3700 per flight

Accommodation: Approximately 71 nights' accommodation for training and data collection at R1200 per night per person.

Per diem: Approximately 71 nights' per diem at R250 per night

Car hire and petrol: Covers car hire (R588 per day), transport for fieldworkers (R900 per bus ticket), travel reimbursements (@ R3.30 per km), and fuel purchases.

Shuttle services: Approximately 76 shuttle drives needed (two shuttle drives needed per flight) at R749 per shuttle.

Phase 2:

Flights: Approximately 20 flights for Miss. Dekel and Miss. Rapiya to the different mortuaries at R3959 per flight

Accommodation: Approximately 40 nights' accommodation needed for Miss. Dekel and Miss. Rapiya to visit the different mortuaries at R1284 per night per person.

Per diem: Approximately 40 nights' per diem needed for Miss. Dekel and Miss. Rapiya at R250 per night

Car hire and petrol: Approximately 40 days' car hire needed for Miss. Dekel and Miss. Rapiya at R629 per day. (Fuel averaging at R321 per day)

Shuttle services: Approximately 40 shuttle drives needed for Miss. Dekel and Miss. Rapiya (two shuttle drives needed per flight) at R450 per shuttle.

<u>Equipment</u>:

20 Tablets (at R2500 per tablet) will need to be purchased in year 1 for 20 data collectors to collect data through the use of REDCap. Miss. Dekel and Miss. Rapiya will also need laptops, purchased on year 1 (Miss. Rapiya) and year 2 (Miss Dekel).

<u>Office Supplies</u>:

Office supplies such as paper, ink cartridges, and stationary will need to be purchased.

<u>Other</u>:

Mobile data:

During phase 1 of the study: the entire research team (Miss. Dekel and Miss. Rapiya and the research assistants: totalling 22 people) will need mobile data (1GB at R80 p/m) for their tablets, in order for captured mortuary data to be uploaded to a central web based database. During phase 2 of the study: Miss. Dekel and Miss. Rapiya and two research assistants, will need mobile data (1GB at R80 p/m) for their tablets, in order for captured police data to be uploaded to a central web based database. Miss. Dekel and Miss. Rapiya will be based in office in year 3 and therefore, there will be no need for data for tablets. Research assistants will not be needed in year 3.

Cell phone Data:

Miss. Dekel and Miss. Rapiya will need monthly cell phone data (at R550 p/m) as well as the assistants who will be allocated R100 for cell phone data (R100 x 20 fieldworkers for 6 months = R9600) while collecting data from mortuaries (phase 1), should they need to contact Miss. Dekel and Miss. Rapiya for assistance/clarification. The two assistants will also be allocated data for phase 2 (R100 x 2 fieldworkers x 12 months = R2400). Miss. Dekel and Miss. Rapiya will need the data to contact Prof. Abrahams while they are in the field (phase 1 and 2), should they need assistance/clarification. Finally, the entire research team will need cell phone data for their cell phone's GPS to assist them in finding the different mortuaries and police stations (phase 1 and 2).

Investigator meeting in Cape Town:

Year 1: One day investigator meeting in CPT:

Flights (Needed for: Prof. Jewkes, Prof. Labuschagne, & Miss Rapiya.) @ R3700 x 3

Shuttles (6 x R450 per shuttle drive)

Car hire (1 x R588)

Catering for total of 11 investigators (11 x R150)

Year 2: One day investigator meeting in CPT:

Flights (Needed for: Prof. Jewkes, Prof. Labuschagne, & Miss Rapiya.) @ R3700 x 3

Shuttles (6 x R450 per shuttle drive)

Car hire (1 x R588)

Catering for total of 11 investigators (11 x R150)

Year 3:

A two-day investigator meeting (housed at MRC CPT) with all co-investigators will take place in year 3 in order to discuss preliminary findings. In order to achieve this, flights, accommodation, shuttle services, a car hire and per diem would be needed for Prof. Jewkes, Prof. Labuschagne, and Miss. Rapiya:

Flights x 6 (@ R4237 per flight): R25 422

Accommodation x 3 (@ R1374): R4122

Shuttle services x 12 (to and from airport) (@ R450): R5400

Car hire x 1 (@R673): R673

Per diem x 3 (@ R306): R918

Feedback meeting with stakeholders in Pretoria:

A one-day feedback meeting with stakeholders (housed at MRC Pretoria) will take place to disseminate findings. In order to achieve this: flights, shuttle services, and a car hire would be needed for Prof. Abrahams, Prof. Mathews, Dr. Richard Matzopoulos, Miss. Prinsloo, and Miss. Dekel:

Flights x 5: R21 180

Shuttle services x 10 (to and from airport): R8570

Car hire x 1: R673

Dissemination of findings:

Research briefs:

R50 000 Layout and printing costs for (1) male, (2) female and (3) child research brief = R150 000.

10. Short and Long-Term Consequences of the Study

This injury mortality study and the homicide study will contribute to updating the data needed for the South African National Burden of Disease Study and male, female and child homicide data. It is our hope that the forthcoming findings will stimulate the development of more closely targeted and sustainable male violence, violence against women and violence against children interventions. It is crucial that these programs are able to bring about a reduction in the murder rate in South Africa. In anticipating adverse outcomes, the wellbeing of fieldworkers is crucial. The fieldworkers will discuss fieldwork experiences, including any distressing issues that emerge, in once a week meetings with the PI, either face to face, over telephone or via skype. All fieldworkers (including Miss Dekel and Miss Rapiya) will be encouraged to access the SAMRC counselling services (at least once every two weeks), accessible at the Cape Town, Pretoria and Durban offices. The counselling services forms part of the SAMRC wellness program. Debriefing is also accessible via Skype. This debriefing will be led by a neutral (non-study team) professional. In addition, a consulting psychologist will be employed during the intense data collection periods, for example, during mortuary data collection. Finally, post-employment psychological support will be provided to assistants for three months after leaving the study.

References

Abrahams, N., Mathews, S., Jewkes, R., Martin, L.J., & Lombard, C. (2012). *Every eight hours: Intimate femicide in South Africa 10 years later*. (Research brief). South African Medical Research Council.

Abrahams, N., Mathews, S., Martin, L. J., Lombard, C., & Jewkes, R. (2013). Intimate partner femicide in South Africa in 1999 and 2009. *PLoS Medicine, 10*(4), e1001412. DOI: 10.1371/journal.pmed.1001412.

Abrahams, N., Mathews, S., Martin, L.J., Lombard, C., Nannan, N., & Jewkes, R. (2016). Gender differences in homicide of neonates, infants, and children under 5 y in South Africa: Results from the cross-sectional 2009 National Child Homicide Study. *PLoS Med, 13*(4), 1-15. DOI:10.1371/journal.pmed.1002003.

Burton, P., Ward, C. L., Artz, L., & Leoschut, L. (2015). *The Optimus study on child abuse, violence and neglect in South Africa*. Cape Town: The Centre for Justice and Crime Prevention.

Divo, M., Cote, C., de Torres, J. P., Casanova, C., Marin, J. M., Pinto-Plata, V., ... & Celli, B. (2012). Comorbidities and risk of mortality in patients with chronic obstructive pulmonary disease. *American journal of respiratory and critical care medicine, 186*(2), 155-161. DOI: 10.1164/rccm.201201-0034OC

Garcia-Moreno, C., & Amin, A. (2016). The sustainable development goals, violence and women's and children's health. *Bulletin of the World Health Organization, 94*(5), 396-397. DOI: 10.2471/BLT.16.172205.

Green-Simms, A. E., Ekdawi, N. S., & Bakri, S. J. (2011). Survey of intravitreal injection techniques among retinal specialists in the United States. *American journal of ophthalmology, 151*(2), 329-332. DOI: 10.1016/j.ajo.2010.08.039

Logan, J. E., Smith, S. G., & Stevens, M. R. (2011). Homicides-United States, 1999-2007. *MMWR supplements, 60*(1), 67.

Mathews, S., Abrahams, N., Jewkes, R., Martin, L. J., & Lombard, C. (2013). The epidemiology of child homicides in South Africa. *Bulletin of the World Health Organization*, *91*(8), 562-568. DOI: 10.1136/injuryprev-2012-040590q.20

Mathews, S., Martin, L. J., Coetzee, D., Scott, C., Naidoo, T., Brijmohun, Y., & Quarrie, K. (2016). The South African child death review pilot: A multiagency approach to strengthen healthcare and protection for children. *SAMJ: South African Medical Journal, 106*(9), 895-899. DOI:10.7196/SAMJ.2016.v106i9.11234

Matzopoulos, R., & Bowman, B. (2016). Sustainable development goals put violence prevention on the map. *Journal of public health policy, 37*(2), 260-262. DOI: 10.1057/jphp.2016.13

Matzopoulos, R., Prinsloo, M., Pillay-van Wyk, V., Gwebushe, N., Mathews, S., Martin, L.J., Laubscher, R., Abrahams, N., Msemburi, W., Lombard, C., & Bradshaw, D. (2015). Injury-related mortality in

South Africa: A retrospective descriptive study of postmortem investigations. *Bulletin of the World Health Organization, 93*, 303-313. DOI: 10.2471/BLT.14.145771.

Msemburi, W., Pillay-van Wyk, V., Dorrington, R. E., Neethling, I., Nannan, N., Groenewald, P., ... & Nojilana, B. (2014). *Second National Burden of Disease Study for South Africa: Cause-of-death profile for South Africa, 1997–2010*. Cape Town: South African Medical Research Council. ISBN: 978-1-920618-35-3.

Outwater, A., Mgaya, E., Campbell, J. C., Becker, S., Kinabo, L., & Menick, D. M. (2010). Homicide of children in Dar es Salaam, Tanzania, 2005. *East African journal of public health, 7*(4), 358-364.

Parliament, S. A. (2003). National Health Act No. 61 of 2003. *Pretoria: Government Printers*.

Parliament, S. A. (2003). Regulations Regarding the Rendering of Forensic Pathology Service (No. 341, 2005). *Pretoria: Government Printers*.

Pinheiro, P. S. (2006). *World report on violence against children*. United Nations.

Seedat, M., Van Niekerk, A., Jewkes, R., Suffla, S., & Ratele, K. (2009). Violence and injuries in South Africa: Prioritising an agenda for prevention. *The Lancet, 374*(9694), 1011-1022. DOI: 10.1016/S0140-6736(09)60948-X.

United Nations Office on Drugs and Crime (UNODC). (2013). *Global study on homicide 2013: Trends, contexts, data*. UNODC.

World Health Organization. (2016). *The ICD-10 classification of mental and behavioural disorders: Clinical descriptions and diagnostic guidelines* (10th ed.). Geneva: World Health Organization.

Appendix A: Confidentiality Form for Fieldworkers

GENDER & HEALTH RESEARCH UNIT

A national study of injury-related mortality, with a focus on homicide in South Africa

Research ID number ______________

I understand that the information collected from both mortuaries and interviews constitutes confidential information. I will not disclose any information that I learn during the data collection.

I hereby agree to keep the information collected from mortuaries and during interviews confidential:

Fieldworker name………………………………..................

Fieldworker signature…………………………………………

Date………………...…

Witness's name………………………………………………..

Witness's signature…………………………………………....

Date………………...

Appendix B: Information Sheet for Police Members Interviewed Telephonically

GENDER & HEALTH RESEARCH UNIT

A national study of injury-related mortality, with a focus on homicide in South Africa

My name is, …………………………, I am a researcher with the Gender & Health Research Unit of the South African Medical Research Council. This study does not involve examining the quality of the investigation nor are we assessing the investigating officer. We are undertaking research into the homicide/murder of men, women and children in South Africa for the year 2017. This will be the first in-depth male homicide/murder study. A similar study was conducted by us in 2009 exploring the murder of women and children. We now want to conduct a follow up study to establish whether we are seeing a difference in the pattern in the murder of women and children in South Africa. We will be comparing female and child murders for 2017 to the data we have for 2009. This will allow us to establish current trends and patterns with regard to female and child murder in South Africa.

Intimate femicide is considered to be the most extreme form and consequence of intimate partner violence and child murder is considered the most extreme form of violence against children. Levels of intimate femicide and child murder can therefore be considered to be an extreme measure of violence against women and children in South Africa. We anticipate that this study will allow us to establish whether the legislation protecting women and children as well as an increase in services targeted at protecting women and children are having an effect. We anticipate that the findings of this research will be very valuable for the police and criminal justice system as well as women and children’s rights groups. The findings will also assist Government departments and non-governmental organisations to consider prevention strategies and improve our understanding of how effective our interventions strategies have been. We have discussed the project with the Ministry of Police and have their full support for the work.

I have identified cases with the following numbers

at ______________________FPS mortuary and from the details held there determined that you are/were the Investigating officer in the case. I would be extremely grateful if you would be prepared to give me some further information about the case. I would like to ask you about the personal details of the victim (but will not ask his/her name); whether there is a known perpetrator and details of him/her (but not his/her name); further details of the crime; and the legal position of the case. All the information should be available in the docket. If you cannot answer any questions because the information is not available or you feel providing me with certain information might compromise the criminal investigation, you should just tell me. We recognise that the answers to all the questions asked will not be available in every case.

The information provided will only be used for research purposes and will not influence the legal case. The research report and any written publications will not include the names of any victims or perpetrators, nor of any officers providing information to the study. I would be extremely grateful if you would be prepared to participate in the study. If you decline, I will ensure that you do not face any problems in your work and that no one knows that I approached you and you declined.

We have obtained permission for the study from Office of the National Commissioner of the South African Police Service. (see attached letter).

If you agree to assist us, please could you sign the attached consent form and either fax it back to me on 021 938 0310 or email me: bianca.dekel@mrc.ac.za and then I will be able to proceed with contacting you by phone. Please retain the original copy for your records.

If you require any further information about the study or clarification please contact:

Prof Naeemah Abrahams: Acting Unit Director
Gender & Health Research Unit: South African Medical Research Council
PO Box 19070
Tygerberg 7505
Tel: 021 938 0448
Fax: 021 938 0310
Email: naeemah.abrahams@mrc.ac.za

If you have further questions about this study, you could contact the SAMRC Ethics Committee:
Secretariat ~~Prof K Moodley~~ (021) 938-0687 or email: adri.labuschagne@mrc.ac.za

Thank you, your help is appreciated

This research has been approved by the South African Medical Research Council Ethics Committee.

Appendix C: Information Sheet for Police Members Interviewed Face-to Face

GENDER & HEALTH RESEARCH UNIT

A national study of injury-related mortality, with a focus on homicide in South Africa

My name is, …………………………., I am a researcher with the Gender & Health Research Unit of the South African Medical Research Council. This study does not involve examining the quality of the investigation nor are we assessing the investigating officer. We are undertaking research into the homicide/murder of men, women and children in South Africa for the year 2017. This will be the first in-depth male homicide/murder study. A similar study was conducted by us in 2009 exploring the murder of women and children. We now want to conduct a follow up study to establish whether we are seeing a difference in the pattern in the murder of women and children in South Africa. We will be comparing female and child murders for 2017 to the data we have for 2009. This will allow us to establish current trends and patterns with regard to female and child murder in South Africa.

Intimate femicide is considered to be the most extreme form and consequence of intimate partner violence and child murder is considered the most extreme form of violence against children. Levels of intimate femicide and child murder can therefore be considered to be an extreme measure of violence against women and children in South Africa. We anticipate that this study will allow us to establish whether the legislation protecting women and children as well as an increase in services targeted at protecting women and children are having an effect. We anticipate that the findings of this research will be very valuable for the police and criminal justice system as well as women and children's rights groups. The findings will also assist Government departments and non-governmental organisations to consider prevention strategies and improve our understanding of how effective our interventions strategies have been. We have discussed the project with the Ministry of Police and have their full support for the work.

I have identified cases with the following numbers

at ______________________FPS mortuary and from the details held there determined that you are/were the Investigating officer in the case. I would be extremely grateful if you would be prepared to give me some further information about the case. I would like to ask you about the personal details of the victim (but will not ask his/her name); whether there is a known perpetrator and details of him/her (but not his/her name); further details of the crime; and the legal position of the case. All the information should be available in the docket. If you cannot answer any questions because the information is not available or you feel providing me with certain information might compromise the criminal investigation, you should just tell me. We recognise that the answers to all the questions asked will not be available in every case.

The information provided will only be used for research purposes and will not influence the legal case. The research report and any written publications will not include the names of any victims or perpetrators, nor of any officers providing information to the study. I would be extremely grateful if you would be prepared to participate in the study. If you decline, I will ensure that you do not face any problems in your work and that no one knows that I approached you and you declined.

We have obtained permission for the study from Office of the National Commissioner of the South African Police Service. (see attached letter).

If you agree to assist us, please could you sign the attached consent form and then we can proceed with the interview.

If you require any further information about the study or clarification please contact:

Prof Naeemah Abrahams: Acting Unit Director
Gender & Health Research Unit: South African Medical Research Council
PO Box 19070
Tygerberg 7505
Tel: 021 938 0448
Fax: 021 938 0310
Email: naeemah.abrahams@mrc.ac.za

If you have further questions about this study, you could contact the SAMRC Ethics Committee:
Secretariat ~~Prof K Moodley~~ (021) 938-0687 or email: adri.labuschagne@mrc.ac.za

Thank you, your help is appreciated

This research has been approved by the South African Medical Research Council Ethics Committee.

Appendix D: Consent Form for Police Members

GENDER & HEALTH RESEARCH UNIT

A national study of injury-related mortality, with a focus on homicide in South Africa

Research ID number _______________

I have read or I have had this information read to me in the information sheet regarding the national study of male, female and child murder study. It is written in a language with which I am fluent and comfortable. I have had a chance to ask questions and all my questions have been adequately answered.

I understand that my participation is voluntary and that I can withdraw from the study at any time and that I have not been pressurised to take part.

I hereby agree to participate in the study:

Yes: ___________________________ ☐

No not interested: _______________ ☐

Participant Name:………...........

Signature:……………………………………………………………………….   Date:…………………………………………….

____

Witness Name:………....

Signature:……………………………………………………………………….   Date:………………………………………………….

Appendix E: Part 1: FPS Homicide Mortuary Data Collection Questionnaire

samrc advancinglife

GENDER & HEALTH RESEARCH UNIT

A national study of injury-related mortality, with a focus on homicide in South Africa

**FPS MORTUARY DATA SHEET**<br>This information is gathered at the FPS mortuary from the death register and FPS mortuary file.			
1	Fieldworker code		
2	Date data collected	[ ] [ ] [ ] [ ] 20 [ ] [ ]<br>D D M M Y Y	
3	Research study number		
4	FPS Mortuary name	FPS Mortuary Name:<br>Province:	
5	FPS Mortuary number		
6	PM Number		
7	Police station (where case reported)		
8	CAS number		
9	Manner of death (from register)/admission history		
**DATA FROM PM REPORT**			

10	Autopsy done	Yes……1<br>No……2<br>Unknown……3	
11	Date of autopsy	[ ] [ ] [ ] [ ] 20 [ ] [ ]<br>D D M M Y Y	
12	Date of death	[ ] [ ] [ ] [ ] 20 [ ] [ ]<br>D D M M Y Y	
13	Date of birth (from official record)<br>If age unknown then enter: 99/99/9999	[ ] [ ] [ ] [ ] 20 [ ] [ ]<br>D D M M Y Y	
14	Age estimated (no official documents)	______ years	
15	Under 1 year	______ months if under 1 year	
16	Under 1 month	______ days if under 1 month (0 days if under 1 day)	
17	Day of the week of death	Monday……1<br>Tuesday……2<br>Wednesday……3<br>Thursday……4<br>Friday……5<br>Saturday……6<br>Sunday……7<br>Unknown……8	
18	Sex of victim	Female ……1<br>Male……2<br>Unknown……3	
19	Race of victim	African ……1<br>Coloured……2<br>White……3<br>Indian/Asian……4<br>Unknown……5	
20	Suspicion of sexual assault	Yes……1<br>No……2<br>Unknown……3	22
21	Was the sexual assault kit used?	Yes……1<br>No……2	
22	Suspicion of pregnancy	Yes……1<br>No……2<br>Unknown……3	
23	Specimen for blood alcohol taken	Yes……1<br>No……2<br>Unknown……3	26
24	FA number for blood alcohol specimens		
25	Alcohol Level g %		

26	External Circumstances of death (from PM report)	Gunshot……1<br>Stab……2<br>Blunt force……3<br>Strangled……4<br>Asphyxiated/smothered……5<br>Fire……6<br>Drowned……7<br>Undetermined by autopsy……8<br>Multiple injuries……9<br>Concealed pregnancy……10<br>Maternal death (abortion related)……11<br>Other……12<br><br>Please specify________________	
**Data on abandoned foetus : Check if Q 16 completed**			
27	Where was the foetus found	Open veld……1<br>Buried in a grave……2<br>Lake/river/dam……3<br>Toilet pit……4<br>Sewage pipe……5<br>Garbage bin/dump……6<br>Other……7<br><br>Please specify________________	
28	Gestation estimated	Yes……1<br>No……2	
29	Gestational age	________________weeks	
30	Viable	Yes……1<br>No……2<br>Unknown……3	
31	Live birth	Yes……1<br>No……2<br>Stillbirth……3<br>Unknown……4	
32	Body dimensions length	________________cm	
33	Body dimensions mass	________________ g	
34	Placenta	Attached……1<br>Detached……2<br>Unknown……3	
35	Umbilical cord	Clamped……1<br>Not clamped……2<br>Unknown……3	

Appendix F: Injury Mortality Survey

BURDEN OF DISEASE RESEARCH UNIT

A national study of injury-related mortality, with a focus on homicide in South Africa

Injury Mortality Survey

A UNIQUE STUDY RECORD NUMBER SHOULD BE CREATED AS THE DATA UPLOADS TO THE CENTRAL DATABASE

1 **What data entry is this?** [Initial | 20th record]   2 **Fieldworker code** [|]

IF "20th record" THEN ALLOW DUPLICATES FOR FIELD 4 "DR NO" AND FIELD 22 "BI-1663"
THIS SHOULD BE LINKED TO MORTUARY CODE

3 **Mortuary code** [|]   4 **DR No.** [| | | | | |]

5 **Record**
- [ ] Found
- [ ] Missing PM
- [ ] Missing folder → EXIT AFTER 11
- [ ] Storage → EXIT AFTER 11

6 **Date of death** [d | d | m | m | yy | yy]

7 **Date of birth** [d | d | m | m | yy | yy]

AUTOMATICALLY CALCULATE AGE: 109 CUT-OFF
IF NO D.O.B *skip* to Estimated Age

8 **Estimated Age** [y | y | y] [m | m]

999 IF UNKNOWN, NO CUT-OFF

9 **Sex** [M | F | U]

10 **Population group** [B | C | A | W | U]

11 **Cause of death**
- [ ] Non-natural PROCEED TO 12
- [ ] Natural EXIT
- [ ] Foetus EXIT
- [ ] Undetermined PROCEED TO 12

12 **Apparent manner of death (if Non-natural)**
- [ ] Homicide ↓ *skip* 13
- [ ] Suicide ↓ *skip* 14
- [ ] Transport ↓ *skip* 15
- [ ] Other Unintentional ↓ *skip* 16
- [ ] Undetermined ↓ *skip* 17

13 **Circumstances of injury (if Homicide)**
- [ ] Firearm Discharge
- [ ] Sharp force (cut / stabbed)
- [ ] Blunt force (beaten with object, punched or kicked)
- [ ] Strangled/Asphyxiated/Suffocated
- [ ] Poison, ingestion
- [ ] Poison, gassing
- [ ] Fire /other burn
- [ ] Abandoned baby (if <1YEAR)
- [ ] Pushed from height
- [ ] Crushing
- [ ] Electrocution
- [ ] Drowning, immersion
- [ ] Explosive blast
- [ ] Other __________
- [ ] Unknown

skip → 18a

14 **Circumstances of injury (if Suicide)**
- [ ] Firearm Discharge
- [ ] Sharp force (cut / slit)
- [ ] Hanging
- [ ] Poison, ingestion (e.g. overdose)
- [ ] Poison, gassing (e.g. exhaust)
- [ ] Fire /other burn
- [ ] Jumped from height
- [ ] Railway pedestrian
- [ ] Other __________
- [ ] Unknown

skip → 18a

15 **Circumstances of injury (if Transport)**

☐	MV Pedestrian	☐	Bicycle
☐	MV Passenger	☐	Railway pedestrian
☐	MV Driver	☐	Railway passenger
☐	MV Unspecified	☐	Aviation casualty
☐	Motor-cycle Driver	☐	Other ________
☐	Motor-cycle Passenger	☐	Unknown

skip → 18a

16 **Circumstances of injury (if Other Unintentional)**

☐	Firearm Discharge	☐	Animal contact (e.g. dog bite)
☐	Sharp force (cut)	☐	Machinery (e.g. farm/recreational)
☐	Blunt force	☐	Lightning
☐	Asphyxiated / Suffocated	☐	Natural/Environmental factors
☐	Poison, ingestion (e.g. overdose)	☐	Electrocution
☐	Poison, gassing (e.g. exhaust)	☐	Explosive blast
☐	Fire /other burn	☐	Circumcision
☐	Fall	☐	Surgical/Medical Misadventure
☐	Crushing	☐	Other ________
☐	Drowning, immersion	☐	Unknown

skip → 18a

17 **Circumstances of injury (if Undetermined)**

☐	Firearm Discharge	☐	Abandoned baby	☐	Animal contact (e.g. dog bite)
☐	Sharp force	☐	MV Pedestrian	☐	Machinery (e.g. farm/recreational)
☐	Blunt force	☐	MV Passenger	☐	Crushing
☐	Asphyxiated / strangled	☐	MV Driver	☐	Lightning
☐	Hanging	☐	MV Unspecified	☐	Natural/Environmental factors
☐	Poison, ingestion	☐	Motor-cycle Driver	☐	Electrocution
☐	Poison, gassing	☐	Motor-cycle Passenger	☐	Explosive blast
☐	Fire /other burn	☐	Bicycle	☐	Circumcision
☐	Fall/push/jump	☐	Railway pedestrian	☐	Surgical/Medical Misadventure
☐	Drowning, immersion	☐	Railway passenger	☐	Other ________
		☐	Aviation casualty	☐	Unknown

18a Was the injury **work-related**? | Y | N | U |

↓ *skip if No/Unknown* 19

18b Was the injury related to **Mining?** | Y | N | U |

19 Was the **"Sexual Assault Kit"** used? | Y | N | U |

20 Was the death in custody (police, prison etc): | Y | N | U |

21 **Blood Alcohol level** | 0 | . | | | | g/100ml

22 **BI-1663** | A | O | | | | | | | | ALLOW DUPLICATE IF FIELD 1="20th record" OTHERWISE MUST BE UNIQUE NUMBER

NOTE: EVERY 20TH RECORD/FOLDER SHOULD BE FLAGGED TO PUT ASIDE FOR DOUBLE DATA ENTRY BY A 2ND FIELDWORKER

Appendix G: Part 2: Police Homicide Data Collection Questionnaire

GENDER & HEALTH RESEARCH UNIT

A national study of injury-related mortality, with a focus on homicide in South Africa

Name of fieldworker: ____ | | |

Date: ___ ___ /___ ___ / 20 ___

D D M M Y Y

Study number: | | | | |

Province name: ____ | | | |

FPS Mortuary name & number: ______________________________________

Police station: ____

[Check that the CAS number and the questionnaire numbers match those on the sheet]

**Docket CAS number:**										

Multiple victims: Yes ……. 1
No……….0

Field edit: ____ (initial)
Study ID Checked: ____ (initial)

CASE INFORMATION FOR FOLLOW- UP AND OFFICE USE

PM Number____
Police Case Number____
Investigating Officer's Name____
Phone number of Investigating Officer____
Initials of person checking form____
Contacts with Police (provide date and type of contact)
1.____
2.____
3.____
4.____

NOTES

Complete before starting the interview

	Female		Male	
Adult female	Y	N	Y	N
Child	Y	N	Y	N
Age of victim				

Form A (1): Adult female victims 18 years and older

Section 1:
Victim background information and circumstances surrounding death

No	Question	Categories	Skip
101	Fieldworker Code		
102	Date of interview	___ ___ ___ ___ 20 ___ ___<br>D D M M Y Y	
103	Research Study Number		
104	Police CAS No		
105	Name of town/village/township/district of injury		
106	Place of injury (complete later)	Major urban centre/provincial capital……1<br>Small town ……2<br>Rural area/village……3<br>Commercial farming area……4<br>Unknown……5	
107	Where did you get the information from?	Investigating Officer doing the investigation……1<br>Info obtained from another SAPS member……2<br>Record review……3<br>No data sourced……4	109
108	What was the reason for not being able to obtain information about the case?	No case traced……1<br>Docket missing……2<br>Other……3<br>Please specify: ______	
**IF NO DATA AVAILABE THEN INTERVIEW CANNOT CONTINUE AND ENDS HERE**			
109	What type of investigation is being followed for this case?	Inquest case……1<br>Other ……2<br>Homicide …… 3<br>Please specify: ______	
110	Was the victim employed at the time of the killing?	Yes……1<br>No……2<br>Unknown ……3	
111	What type of work did she normally do?	Unemployed/Homemaker……1<br>Professional: Nurse, doctor, lawyer, accountant, social worker……2<br>White collar: Secretary, office or bank worker, sales person……3	

		Blue collar: Factory worker, waitress, employed in a shop……………………4<br>Sex worker……………………………………………………………………………5<br>Domestic work/gardening /cooking………………………………………………6<br>Selling/ trading or making/growing things to sell……………………………7<br>Farm worker……………………………………………………………………………8<br>Security industry: Police, armed force, security guard……………………9<br>Street person…………………………………………………………………………10<br>Scholar…………………………………………………………………………………11<br>Student (post school)………………………………………………………………12<br>Unknown………………………………………………………………………………13<br>Other ……………………………………………………………………………………14<br><br>Please specify: ______________________________	
112	Where was the victim last seen?	Home (own)……………………………………………………………………………1<br>Someone else home …………………………………………………………… 2<br>Work………………………………………………………………………………………3<br>Walking home…………………………………………………………………………4<br>Bar/shebeen……………………………………………………………………………5<br>Street……………………………………………………………………………………6<br>Park/open ground……………………………………………………………………7<br>School……………………………………………………………………………………8<br>Unknown…………………………………………………………………………………9<br>Other ……………………………………………………………………………………10<br><br>Please specify: ______________________________	
113	Where was the victim injured	Victims home……………………………………………………………………………1<br>Perpetrator's home …………………………………………………………………2<br>Other home/Yard………………………………………………………………………3<br>Please specify: ______________________________<br><br>Public Space ……………………………………………………………………………4<br>Victim's workplace……………………………………………………………………5<br>Place Unknown…………………………………………………………………………6<br>Other ……………………………………………………………………………………7<br>Please specify: ______________________________	
114	Was more than one victim involved?	Yes…………………………………………………………………………………………1<br>No…………………………………………………………………………………………2	 119
115	If yes, total number of victims (Include injured and deceased)		
116	Number of victims who died		
117	Who were these other victims who were killed or injured? (You can have more than one category)	Victim Parent/s………………………………………………………………………1<br>Victim Sibling/s………………………………………………………………………2<br>Victim's children ……………………………………………………………………3<br>Victims husband/boyfriend…………………………………………………………4<br>Victim's other relative/s……………………………………………………………5<br>Neighbour/s……………………………………………………………………………6<br>Friends/Acquaintances………………………………………………………………7<br>Passerby/strangers……………………………………………………………………8	

		Other…………………………………………………………………………………9<br>Please specify: ________________________________	
118	Were members of the nuclear family killed in the incident?	Yes……………………………………………………………………………………1<br>No……………………………………………………………………………………2	
119	Did the perpetrator commit suicide?	Yes……………………………………………………………………………………1<br>No……………………………………………………………………………………2<br>Unknown……………………………………………………………………………3	 201
120	How many days after the killing did he commit suicide?	Within 24 hrs………………………………………………………………………1<br>Between 2-7 days…………………………………………………………………2<br>After 7 days…………………………………………………………………………3	
**Section 2: Perpetrator information**			
Now I will be asking you about the person who is suspected or known to have committed the crime			
201	Is the perpetrator of crime known/suspect or unknown?	Known/suspect……………………………………………………………………1<br>Unknown……………………………………………………………………………2	
202	Was more than one person known or suspected to have committed the crime?	Yes……………………………………………………………………………………1<br>No……………………………………………………………………………………2<br>Unknown……………………………………………………………………………3	 205<br>205
203	If yes, how many other perpetrators were involved?	________________________________	
204	We would now like to continue to collect information on the person who is thought to be the main perpetrator. Were you able to identify a main perpetrator?	Yes……………………………………………………………………………………1<br>No……………………………………………………………………………………2<br>Please explain: ________________________________<br>________________________________<br>________________________________<br>All perpetrators equally important…………………………………………3<br>Please explain________________________________<br>________________________________<br>________________________________	
205	What was the relationship known? i.e. relationship between suspected perpetrator to victim?	Husband (any legal or customary marriage or completed lobola)……………1<br>Ex-husband…………………………………………………………………………2<br>Cohabitating boyfriend (Partial lobola or no marriage)…………………………3<br>Ex- cohabitating boyfriend (partial lobola or no marriage)……………………4<br>Boyfriend (current) ………………………………………………………………5<br>Ex- boyfriend………………………………………………………………………6<br>Same Sex Partner…………………………………………………………………7<br>Rejected man proposing a relationship…………………………………………8<br>Biological father……………………………………………………………………9<br>Step father/ mother's boyfriend…………………………………………………10<br>Mother………………………………………………………………………………11<br>Foster parent/guardian …………………………………………. …………………12<br>Other relative………………………………………………………………………14<br>Please specify: ________________________________<br><br>In-laws………………………………………………………………………………13<br>Friend/ Person know by sight/ acquaintance ……………………………………14<br>Stranger……………………………………………………………………………15<br>Female perpetrator romantically involved with victims current or ex-husband/boyfriend (love triangle) ……………………………………………………16	

		Unknown……17<br>Other……18<br><br>Please specify: ______________________	
206	What is the sex of the perpetrator?	Male……1<br>Female……2<br>Unknown……3	
207	Is the age of the perpetrator known, unknown or estimated?	Known……1<br>Estimated……2<br>Unknown……3	 <br>209
208	How old is the perpetrator?	______________________	
209	What is the race of the perpetrator?	African……1<br>Coloured……2<br>White……3<br>Indian/Asian……4<br>Unknown……5	
210	Did the perpetrator work at the time of the crime?	Yes……1<br>No……2<br>Unknown……3	
211	What type of work did he/she normally do?	Professional: nurse, doctor, teacher, lawyer, accountant, etc……1<br>Business man owns a company ……2<br>White collar: secretary, office or bank worker, sales person etc……3<br>Blue collar: factory worker, builder, waiter, postman etc…… 4<br>Security Industry: Police……5<br>Security Industry: Army……6<br>Security Industry: Private sector……7<br>Domestic worker/gardening/ car guard/ driver……8<br>Taxi owner/ driver or bus driver……9<br>Selling/trading or making/ growing things to sell……10<br>Farm worker……11<br>Scholar……12<br>Student (post school)……13<br>Farm worker……14<br>Street person……15<br>Employed but occupation unknown……16<br>Other……17<br>Please specify: ______________________	
212	Was the perpetrator known to drink heavily?<br>(Heavy meaning two or more alcoholic beverages per day)	Yes……1<br>No……2<br>Unknown……3	
213	Was the perpetrator known to use illegal drugs?	Yes……1<br>No……2<br>Unknown……3	
214	Did he own one or more legal firearms?	Yes……1<br>No……2<br>Unknown……3	

215	Did he own one or more illegal firearms?	Yes…1<br>No…2<br>Unknown…3	
216	Does he have any prior convictions?	Yes, time served in prison…1<br>Yes, suspended sentence…2<br>Yes, out on bail…3<br>No…4<br>Unknown…5	 <br><br>218<br>218
217	Specify what the previous conviction/s were for?	________________________________	
218	Where is the perpetrator now?	Arrested, waiting trial in prison…1<br>Out on bail…2<br>Out on bail but whereabouts unknown…3<br>Suspected, not enough evidence to arrest…4<br>Whereabouts Unknown…5<br>Other…6<br>Please specify: ________________________	
219	Did death occur in the context of another crime?	Yes…1<br>No…2<br>Unknown…3	 221<br>221
220	Specify the primary context of the "other" crime	Robbery…1<br>Housebreaking/theft…2<br>Hijacking…3<br>Taxi Violence…4<br>Gang violence…5<br>Rape …6<br>Abduction…7<br>Other …8<br>Please specify: ________________________	
221	Was rape/sexual assault suspected?	Yes…1<br>No…2<br>Unknown…3	
222	Did the perpetrator make a formal written confession?	Yes…1<br>No…2<br>Unknown…3	

Form B: Perpetrator/Intimate partner information			
Check question 205 for the victim perpetrator relationship. if the perpetrator fit the following criteria: the victim was involved with the perpetrator in an intimate relationship (current or ex- husband, current or ex- boyfriend, current or common-law partner, a person who was rejected by the victim after he showed a romantic interest, same sex partners). Now continue to ask the investigating officer about the victim-perpetrator relationship if the perpetrator is an intimate partner.			
301	Were the victim and perpetrator living together at the time of the incident?	Yes……1<br>No……2<br>Unknown……3	
302	Had the perpetrator ever been physically violent towards the victims?	Yes……1<br>No……2<br>Unknown……3	
303	Had she previously reported the perpetrator to the police?	Yes……1<br>No……2<br>Unknown……3	
304	Has she ever previously applied for a protection order or interdict?	Yes……1<br>No……2<br>Unknown……3	306
305	If yes, did she have a PO at the time of the murder?	Yes……1<br>No……2<br>Unknown……3	
306	Had the police ever been called out to her home because of fighting between the perpetrators and victim?	Yes……1<br>No……2<br>Unknown……3	
307	Had the perpetrator ever previously been arrested for assault or threats on the victim?	Yes……1<br>No……2<br>Unknown……3	
308	Had the perpetrator previously injured the victim so that she had had medical treatment?	Yes……1<br>No……2<br>Unknown……3	
309	Events leading to the murder	Female partner ending relationship……1<br>Argument over money……2<br>Argument over male partner's infidelity……3<br>Argument: reason unknown……4<br>Female partner did not obey male partner……5<br>Female partner refused to have sex with male partner……6<br>Female partner "nagging" male partner……7<br>Alleged infidelity of female partner ……8<br>Female partner alleged to have insulted male partner's sexual prowess……9<br>Financial gain……10<br>Accident……11<br>No explanation……12<br>Other……13<br>Please specify:<br>____	

310	What was the status of the relationship at the time of the murder?	Couple living together…………………………1<br>Couple divorced…………………………2<br>Couple in the process of separating…………………………3<br>Couple separated…………………………4<br>Couple dating and living in separate households…………………………5<br>Other …………………………6<br>Please specify:<br>____	312
311	If separated/ separating who initiated the separation?	Man…………………………1<br>Woman…………………………2<br>Unknown…………………………3	
312	Did they have children together?	Yes…………………………1<br>No…………………………2<br>Unknown…………………………3	
313	Were there any step children living with them?	Yes…………………………1<br>No…………………………2<br>Unknown…………………………3	 End
314	Whose children (in question 313) were they?	Victim…………………………1<br>Perpetrator…………………………2	

Form A(2): Children under the age of 18 years

Section 1:
Victim background information and circumstances surrounding death

The first questions I want to ask you are about the victim and the circumstances around the death. Please try and relax, there are no right or wrong answers. Remember that everything you tell me will be kept confidential. If there is a question you do not want to answer please tell me and we will skip to the next question

No	Question	Categories	Skip
401	Fieldworker Code		
402	Date of interview	___ ___ ___ ___ 20 ___ ___<br>D D M M Y Y	
403	Research Study Number		
404	Police CAS No		
405	Name of town/village/township or district of injury		
406	Place of injury (complete later)	Major urban centre/provincial capital…………………………1<br>Small town …………………………2<br>Rural area/village…………………………3<br>Commercial farming area…………………………4<br>Unknown…………………………5	
407	Where did you get the information from?	Investigating Officer doing the investigation…………………………1<br>Info obtained from another officer…………………………2<br>Record review…………………………3<br>No data sourced…………………………4	410<br>410<br>410

408	What was the reason for not being able to obtain information about the case?	No case traced……………………………………………………………………1<br>Docket missing…………………………………………………………………2<br>Other………………………………………………………………………………3<br>Please specify: ___________________________	
409	If no data available then interview cannot continue and ends here<br>If abandoned newborn/fetus- provide detail of how body was found (find this in the police form in the FPS mortuary file)		
410	What type of investigation is being followed for this case?	Inquest case……………………………………………………………………1<br>Other ……………………………………………………………………………2<br>Please specify: ___________________________	
411	Can you tell me who was taking care of the child at the time his/her death?<br><br>Code this question afterwards – make notes on the response	___________________________<br>___________________________<br>___________________________<br>___________________________<br>___________________________<br><br>Codes:<br>Biological mother and father…………………………………………1<br>Biological Mother only………………………………………………2<br>Biological Father only………………………………………………3<br>Grandparent……………………………………………………………4<br>Relative…………………………………………………………………5<br>Please specify: ___________________________<br><br>Foster parents…………………………………………………………6<br>Children home…………………………………………………………7<br>On the street……………………………………………………………8<br>Unknown…………………………………………………………………9<br>Other……………………………………………………………………10<br>Please specify: ___________________________	
412	Only ask question if 6 years old or more<br>(if younger mover to Q 414)<br>Was the victim at school at the time of the murder?	Yes…………………………………………………………………………1<br>No……………………………………………………………………………2<br>Unknown…………………………………………………………………3	
413	What is the highest standard or grade the victim completed at school?	Grade R……………………………………………………………………1<br>SUB A/ Grade 1…………………………………………………………2<br>SUB B/ Grade 2…………………………………………………………3<br>STD 1/ Grade 3…………………………………………………………4<br>STD 2/ Grade 4…………………………………………………………5<br>STD 3/ Grade 5…………………………………………………………6<br>STD 4/ Grade 6…………………………………………………………7<br>STD 5/ Grade 7…………………………………………………………8<br>STD 6/ Grade 8…………………………………………………………9<br>STD 7/ Grade 9…………………………………………………………10<br>STD 8/ Grade 10………………………………………………………11<br>STD 9/ Grade 11………………………………………………………12	

		STD 10/ Grade 12……………………………………………………………………13<br>Incomplete further degree or qualification……………………………………14<br>Never attended School ………………………………………………………………15<br>Unknown……………………………………………………………………………………16	
414	If not a newborn,<br>Where was the victim last seen?	Home (own)…………………………………………………………………………………1<br>Someone else home …………………………………………………………………… 2<br>Work…………………………………………………………………………………………3<br>Walking home……………………………………………………………………………4<br>Bar/shebeen………………………………………………………………………………5<br>Street…………………………………………………………………………………………6<br>Park/open ground……………………………………………………………………7<br>School………………………………………………………………………………………8<br>Unknown……………………………………………………………………………………9<br>Other ………………………………………………………………………………………10<br>Please specify: ____	
415	Where was the victim injured	Victims home……………………………………………………………………………1<br>Perpetrator's home ……………………………………………………………………2<br>Other home/Yard………………………………………………………………………3<br>Please specify: ____<br><br>Public Space ………………………………………………………………………………4<br>Victim's workplace……………………………………………………………………5<br>Place Unknown……………………………………………………………………………6<br>Other ………………………………………………………………………………………7<br><br>Please specify: ____	
416	Was more than one victim involved?	Yes……………………………………………………………………………………………1<br>No……………………………………………………………………………………………2	421
417	If yes, total number of victims (include injured and died)		
418	Number of victims who died		
419	Who were these other victims who were killed or injured? (You can have more than one category)	Father………………………………………………………………………………………1<br>Mother ………………………………………………………………………………………2<br>Victim Sibling/s…………………………………………………………………………3<br>Victim's children ………………………………………………………………………4<br>Victims husband/boyfriend/ girlfriend ……………………………………………5<br>Victim's other relative/s………………………………………………………………6<br>Neighbour/s………………………………………………………………………………7<br>Friends/Acquaintances…………………………………………………………………8<br>Passerby/strangers………………………………………………………………………9<br>Other…………………………………………………………………………………………10<br>Please specify: ____	
420	Were members of the nuclear family killed in the incident?	Yes……………………………………………………………………………………………1<br>No……………………………………………………………………………………………2	
421	Did the perpetrator commit	Yes……………………………………………………………………………………………1	

	suicide?	No……………………………………………………………………………………………………2	501
422	How many days after the killing did he commit suicide?	Within 24 hrs…………………………………………………………………………………1<br>Between 2-7 days……………………………………………………………………………2<br>After 7 days……………………………………………………………………………………3	

Section 2: Perpetrator information			
Now I will be asking you about the person who is suspected or known to have committed the crime			
501	Is the perpetrator of crime known, unknown or a suspect?	Known……………………………………1<br>Suspected……………………………………2<br>Unknown……………………………………3	
502	Was more than one person known or suspected to have committed the crime?	Yes……………………………………1<br>No……………………………………2<br>Unknown……………………………………3	 504<br>504
503	If yes, how many other perpetrators were involved?	______________________________	
504	We would now like to continue to collect information on the person who is thought to be the main perpetrator. Were you able to identify a main perpetrator?	Yes……………………………………1<br>No……………………………………2<br>Please explain______________________<br>______________________________<br>______________________________<br><br>All perpetrators equally important……………………3<br>Please explain______________________<br>______________________________<br>______________________________	
505	What is the sex of the perpetrator?	Male……………………………………1<br>Female……………………………………2<br>Unknown……………………………………3	
506	Can you tell me if there was a relationships between the victim and the known/suspected perpetrator ?<br>Let the IO describe – code later	______________________________<br>______________________________<br>______________________________<br>______________________________<br>______________________________<br><br>**Codes:**<br>Husband/wife (any legal or customary marriage or completed lobola)……1<br>Ex-husband……………………………………2<br>Cohabitating boy/girlfriend (partial lobola or no marriage)……………3<br>Ex- cohabitating boy/girlfriend (partial lobola or no marriage)…………4<br>Boy/girlfriend (current) ……………………………5<br>Ex- boy/girlfriend……………………………………6<br>Same sex Partner……………………………………7<br>Rejected person proposing a relationship…………………8<br>Biological Father……………………………………9<br>Step father/mother or a parents boy/girlfriend……………10<br>Biological Mother……………………………………11<br>Other relative……………………………………12<br>Please specify_______________________<br><br>In-laws……………………………………13<br>Friend/person know by sight/acquaintance ……………………14<br>Stranger……………………………………15<br>Female perpetrator romantically involved with victims current or ex-husband/wife/boy/girlfriend (love triangle) ……………………16	

		Child carer……17<br>Unknown……18<br>Other……19<br>Please specify________________________	
507	Was there a suspicion of child abuse/incest or concealment of pregnancy?	Yes confirmed……1<br>Suspected only……2<br>Ruled out ……3<br>Unknown……4	
508	How old is the perpetrator?	________________________	
509	What is the race of the perpetrator?	African……1<br>Coloured……2<br>White……3<br>Indian/Asian……4<br>Unknown……5	
510	Did the perpetrator work at the time of the crime?	Yes……1<br>No……2<br>Unknown……3	
511	What type of work did he/she normally do?	Professional: Nurse, doctor, teacher, lawyer, accountant, etc……1<br>Business man owns a company ……2<br>White collar: secretary, office or bank worker, sales person etc……3<br>Blue collar: factory worker, builder, waiter, postman etc…… 4<br>Security Industry: Police/……5<br>Security Industry army……6<br>Security Industry: Private sector……7<br>Domestic worker/gardening/ car guard/ driver……8<br>Taxi owner/driver or bus driver……9<br>Selling/trading or making/growing things to sell……10<br>Farm worker……11<br>Scholar……12<br>Student (post school)……13<br>Farm worker……14<br>Street person……15<br>Employed but occupation unknown……16<br>Other……17<br>Please specify________________________	
512	Was the perpetrator known to drink heavily?	Yes……1<br>No……2<br>Unknown……3	
513	Was the perpetrator known to use drugs?	Yes……1<br>No……2<br>Unknown……3	
514	Did he own one or more legal guns?	Yes……1<br>No……2<br>Unknown……3	
515	Did he own one or more illegal guns?	Yes……1<br>No……2<br>Unknown……3	
516	Does he have any prior convictions?	Yes, time served in prison……1<br>Yes, suspended sentence……2	

		Yes, out on bail…………………………………………3<br>No……………………………………………………4<br>Unknown………………………………………………5	 518<br>518
517	Specify what the previous conviction were for?	______________________________	
518	Where is the perpetrator now?	Arrested, awaiting trial in prison…………………………1<br>Out on bail……………………………………………2<br>Out on bail but whereabouts unknown………………………3<br>Suspected, not enough evidence to arrest…………………4<br>Whereabouts Unknown……………………………………5<br>Other…………………………………………………6<br>Please specify______________________________	
519	Did death occur in the context of another crime?	Yes……………………………………………………1<br>No……………………………………………………2<br>Unknown………………………………………………3	 521<br>521
520	Specify the primary context of the "other" crime	Robbery………………………………………………1<br>Housebreaking/theft……………………………………2<br>Hijacking……………………………………………3<br>Taxi Violence…………………………………………4<br>Gang violence…………………………………………5<br>Rape /incest…………………………………………6<br>Abduction……………………………………………7<br>Other …………………………………………………8<br>Please specify______________________________	
521	Was rape/sexual assault suspected?	Yes……………………………………………………1<br>No……………………………………………………2<br>Unknown………………………………………………3	
**Check question 506**<br>**If victim was killed by an intimate partner complete form b of adult questionnaire**<br>**Continue with form C for all children**			

Form C: Children: Child abuse/neglect

We want to find out more about the social context in which children lived and ask the following questions for all children

601	Does the child and the perpetrator live in the same house	Yes……………………………………………………1<br>No……………………………………………………2	
602	Is the child being cared for by the perpetrator during the day	Yes……………………………………………………1<br>No……………………………………………………2	
603	Was the child known to have a chronic illness or a disability	Yes……………………………………………………1<br>No……………………………………………………2	
604	What is the age of the mother	______________________________	
605	What is the age of the father	______________________________	

606	Does the mother receive a government grant for the child	Yes...1<br>No...2	
607	Was the child referred to a social worker or other social care?	Yes…………………………………………………………………………………1<br>No………………………………………………………………………………….2<br>Unknown…………………………………………………………………………3	
608	Was the child seen by a social worker or any other form of social care?	Yes…………………………………………………………………………………1<br>No………………………………………………………………………………….2<br>Unknown…………………………………………………………………………3	610
609	Can you tell me why the child was referred or seen by a social worker or other social care/support?<br><br>Let the IO describe this and code it later	____________________________________<br>____________________________________<br>____________________________________<br>____________________________________<br>____________________________________<br><br>**Codes:**<br>Child is in foster care/children home...................................1<br>Child has been to juvenile jail/court/special centre/children's home...2<br>Child neglect known or suspected......................................3<br>Child abuse known or suspected ……………………….................4<br>Child abandoned…………………………………………………………5<br>Mother/ Father died…………………………………………………….6<br>Behaviour problems……………………………………………………7<br>Please specify______________________________<br>____________________________________<br>____________________________________<br><br>Other …………………………………………………………………………8<br>Please specify______________________________<br>____________________________<br>____________________________________<br>____________________________________	
610	Did mother have another child or other children who had died?	Yes…………………………………………………………………………………1<br>No………………………………………………………………………………….2<br>Unknown…………………………………………………………………………3	 701<br>701
611	How many had died	____________________________________	
612	What did they die of?		

Form D: Court processes

Section 1: Charges [this refers to when the suspect is formally charged in court]

701	On what date was the accused charged in court?	___ ___ /___ ___ / ___ ___<br>D D M M Y Y	
702	How many accused	____	

	appear on the charge sheet/indictment page?		
703	What was the accused charged with in court in connection with the murder?	Murder ……………………………………1<br>Attempted murder ……………………………………2	
704	Number of other charges brought against the accused?	[ ] [ ] 0 0 if none	801
705	Specify the other charges brought against the accused?	a) Robbery……………………………a) Yes 1 No 0<br>b) Housebreaking……………………………b) Yes 1 No 0<br>c) Attempted murder……………………………c) Yes 1 No 0<br>d) Kidnapping……………………………d) Yes 1 No 0<br>e) Pointing a firearm……………………………e) Yes 1 No 0<br>f) Assault ……………………………f) Yes 1 No 0<br>g) Sexual assault……………………………g) Yes 1 No 0<br>h) Other ……………………………h) Yes 1 No 0	

Form D: Section 2: Bail

801	Did the accused apply for bail?	Yes…………………………………… 1<br>No……………………………………0<br>Matter withdrawn before bail application……………………2	803
802	Which of the following grounds for release did the accused put forward in his/her bail application?	a) Fixed address and length of time lived there ……………a) Yes 1 No 0<br>b) No current cases against accused ……………b) Yes 1 No 0<br>c) No previous convictions ……………c) Yes 1 No 0<br>d) No passport or relatives in foreign country ……………d) Yes 1 No 0<br>e) Accused will not interfere with police investigation……………e) Yes 1 No 0<br>f) Accused will stick to bail conditions ……………f) Yes 1 No 0<br>g) Weak state case ……………g) Yes 1 No 0<br>h) Accused currently employed ……………h) Yes 1 No 0<br>i) Accused has dependent/s ……………i) Yes 1 No 0<br>j) Medical condition which needs treatment……………j) Yes 1 No 0<br>k) Other ……………h) Yes 1 No 0<br><br>Please specify____<br>____	
803	If accused was a minor, was he/she	a) Put through a formal bail hearing……………a) Yes 1 No 0<br>b) Released into parental/legal guardian's custody…………. ………b) Yes 1 No 0<br>c) Released into a place of safety……………c) Yes 1 No 0	
804	On what date(s) was the first bail hearing?	___ ___ /___ ___ / ___ ___<br>D D M M Y Y<br><br>___ ___ /___ ___ / ___ ___<br>D D M M Y Y	

		___ ___ /___ ___ / ___ ___<br>D D M M Y Y	
805	Did the state oppose bail?	Yes……………………1<br>No……………………0	807
806	If yes, on what grounds was bail opposed?	a) Attempts to evade trial……………a) Yes 1 No 0<br>b) Attempts to influence or intimidate witness/conceal or destroy evidence ……………b) Yes 1 No 0<br>c) Interferes with justice system…………… c) Yes 1 No 0<br>d) Disturbs the public order, peace and security……………d) Yes 1 No 0<br>e) Endangers the safety of the public/particular individual or commit a schedule 1 offence ……………e) Yes 1 No 0<br>f) Schedule 6 offence[4]……………f) Yes 1 No 0<br>g) No fixed address ……………g) Yes 1 No 0<br>h) Previous convictions……………h) Yes 1 No 0<br>i) Victim lives in same area as accused……………i) Yes 1 No 0<br>j) Unknown……………j) Yes 1 No 0<br>k) Other ……………k)Yes 1 No 0	
807	Was the accused granted bail?	Yes…………………… 1<br>No……………………0	901
808	How much was bail posted at?	R________________________	
809	Were the conditions attached to the bail?	a) Magistrate cites usual conditions……………a) Yes 1 No 0<br>b) No contact with complainant/witnesses……………b) Yes 1 No 0<br>c) Report to person of authority at specific times……………c) Yes 1 No 0<br>d) Not to leave the province/country……………d) Yes 1 No 0<br>e) Not to destroy or interfere with evidence……………e) Yes 1 No 0<br>f) Restriction of accused's movements……………f) Yes 1 No 0<br>g) Anything additional confirmed by the court……………g) Yes 1 No 0	
810	Did the accused breach or skip bail?	Yes…………………… 1<br>No……………………0	901
811	If yes, was bail revoked?	Yes……………………1<br>No……………………0	
812	Did the perpetrator commit further offences while out on bail?	Yes…………………… 1<br>No……………………0<br><br>Please specify offence:________________________	

Form D: Section 3: Plea and sentence agreements (Plea bargains)

901	Did the accused plead guilty to murder?	Guilty……………………1<br>Not guilty……………………0<br>Did not plead……………………2	903

[4] Schedule 6 offences include premeditated murder; murder of a law enforcement officer; murder of a witness; multiple rape; gang rape; armed robbery; robbery causing grievous bodily harm; and indecent assault on a child under the age of 16 years involving the infliction of grievous bodily harm.

902	Was a sentence agreement entered into?	Yes……………………………………………………1<br>No……………………………………………………0	
903	What was the sentence handed down?	a) Fine……………………………a) Yes 1 No 0<br>b) Prison sentence……………………………b) Yes 1 No 0<br>c) Suspended prison sentence……………………c) Yes 1 No 0<br>d) Correctional supervision……………………d) Yes 1 No 0	

Form D: Section 4: Outcome of case (information from charge sheet)			
1001	On what date was the outcome (guilty charge) of the trial?	___ ___ /___ ___ / ___ ___<br>D D M M Y Y	
1002	In relation to the murder charge, was the accused found:	Guilty of murder……………………………………1<br>Guilty of another crime……………………………2<br>Not guilty/acquitted……………………………3<br>Discharged in terms of Section 174[5]……………………4<br>Matter stopped by the prosecution in terms of Section 6……………5	
1003	What was the sentence handed down?	a) Fine……………………………a) Yes 1 No 0<br>b) Prison sentence……………………………b) Yes 1 No 0<br>c) Suspended prison sentence……………………c) Yes 1 No 0<br>d) Correctional supervision……………………d) Yes 1 No 0	
1004	What was the sentence of imprisonment?	[ ] [ ] years<br>Life sentence……………………………………1<br>Unknown……………………………………0	

Form E: Investigating officer		
We are near the end and I would now like to ask you a few questions about yourself and your work as an investigating officer as we will be using this information to make recommendations regarding the training of investigating officers		
1101	Was this IO interviewed by us before in the study	Yes……………………………………1<br>No……………………………………2
1102	Gender of the IO	Male……………………………………1<br>Female……………………………………2
1103	How long have you been a detective?	________________________
1104	How long have you been investigating murder cases?	________________________
1105	What was/is your rank in the police service when you received the case to investigate?	________________________

[5] 174. Accused may be discharged at close of case for prosecution.— If, at the close of the case for the prosecution at any trial, the court is of the opinion that there is no evidence that the accused committed the offence referred to in the charge or any offence of which he may be convicted on the charge, it may return a verdict of not guilty

1106	Did you receive training in investigating cases of domestic violence?	Yes..1<br>No..2
1107	Did you receive training in the investigation of sexual assault cases?	Yes..1<br>No..2
1108	Did you receive training in the investigation of murder cases?	____________________________________
1109	How would you score your skills to attend to and investigate domestic violence cases	Excellent..1<br>Good..2<br>Can be improved..3<br>Poor..4
1110	How would you score your skills to attend to and investigate to cases child abuse	Excellent..1<br>Good..2<br>Can be improved..3<br>Poor..4
1111	Have you completed any of the following courses	Detective Learning Program (or equivalent for older members)<br>Serious and Violent Crime Course<br>Psychologically Motivated Crimes/Investigative Psychology Course<br>FCS Course<br>Organised Crime Course<br>Other: _____________________

**To be completed by the person checking the form**		
	Based on the above information, what type of murder was this?	Female homicide..1<br>Intimate homicide..2<br>Child homicide..3<br>Unknown..4<br>Suspected female homicide..5<br>Suspected intimate homicide..6<br>Suspected child homicide..7

ENDING: Thank the IO. and ask him/her if he/she has any questions

Appendix H: Principle Investigator CV (Prof. Naeemah Abrahams)

BIOGRAPHICAL SKETCH

**NAME**	**POSITION TITLE**
Naeemah Abrahams	Acting Unit Director<br>Gender & Health Research Unit<br>South African Medical Research Council

EDUCATION/TRAINING

**INSTITUTION AND LOCATION**	**DEGREE**	**YEAR**	**FIELD OF STUDY**
Nico Malan Nursing College	General Nursing	1982	Nursing
Bellville Technicon	Community Nurs	1984	Nursing
Red Cross Children's Hospital	Pediatric Nurs	1987	Nursing
University of Western Cape (South Africa)	MPH	1997	Public Health
University of Cape Town (South Africa)	Ph.D.	2002	Public Health

A. POSITIONS AND HONOURS

2014-present Acting Unit Director: Gender & Health Research Unit (GHRU), South African Medical Research Council (SAMRC)

AWARDS

Finalist in the NRF Black Scientist of the Year award - 2005

Awarded MRC Flagship Award for Rape Impact Cohort Study (RICE) - Feb 2014 – Jan 2018 Silver Medalist for the MRC Scientific Merit awards – October 2013

PERSONAL STATEMENT:

Naeemah is a Chief Specialist Scientist and Unit Director in the Gender & Health Research Unit of the South African Medical Research Council. She is part of the Gender & Health Research Unit research team that have been acknowledged as world leaders on gender based violence and health research. She is an author on more than 50 peer reviewed journal articles and numerous reports and policy briefs. She is a Principal Investigator and Co-investigator on multiple research projects all linked to gender based violence and the interface with health. She has two honorary appointments: as an Associate Professor with University of Cape Town and as Extraordinary Professor with University of Western Cape.

CURRENT RESEARCH PROJECTS

1. The impact of rape in women on HIV acquisition and retention and linkages to care: a longitudinal study (Rape Impact Cohort Study (RICE)) (Principal Investigator)
2. Developing and pilot testing a gender-based HIV AIDS risk reduction and coping skills intervention for HIV positive men and women in South Africa (Principal Investigator)
3. Research on exploring the social context in which intimate femicide occurs (Co-Investigator)
4. An exploration of disabled women's experiences of violence in South Africa. (Co-Investigator)
5. Research in female and child murders in South Africa 2009: comparing the epidemiology of the 1999 femicide study with 2009 as well as describing the epidemiology of child murders (Principal Investigator)
6. Research on the mental health services for rape survivors in primary health care settings (Principal Investigator).
7. Global Burden of disease 2010 study: Systematic review of prevalence and health effects of Intimate Partner Violence, Childhood Sexual Abuse and Non-Partner Sexual Violence reviews (Co-investigator in collaboration with LSHTM and WHO)
8. Research on the development and pilot testing of interventions for HIV positive women (Principal investigator)
9. Research on the associations between HIV and IPV during pregnancy in Zimbabwe (Co-investigator)

10. Research on sexual violence in UCT students residences (Co-investigator)
11. Research on violence among disabled women (Co-investigator)
12. Research on estimating the burden of disease in South Africa (violence and Injury Foci). (Co-investigator)
13. Research on the global prevalence of child homicide

PEER REVIEWED PUBLICATIONS

1. Matzopoulos R., M. Prinsloo, V. Pillay-van Wyk, N. Gwebushe, S. Mathews, L. J. Martin, R. Laubscher, **N. Abrahams**, W. Msemburi, C. Lombaard and D. Bradshaw (2015). "Injury-related mortality in South Africa: a retrospective descriptive study of postmortem investigations." Bulletin of the World Health Organization **93**: 303-313
2. **Abrahams, N**., K. Devries, C. Watts, C. Pallitto, M. Petzold, S. Shamu and C. García-Moreno (2014). "Prevalence of non-partner sexual violence: a review of global data." Oxford Textbook of Violence Prevention: Epidemiology, Evidence, and Policy: 49
3. Mathews, C. and **N. Abrahams** (2014). "5.4 Gender Issues and the Burden of Disease in Women." HIV and Psychiatry: 256
4. García-Moreno, C., C. Zimmerman, A. Morris-Gehring, L. Heise, A. Amin, **N. Abrahams**, O. Montoya, P. Bhate-Deosthali, N. Kilonzo and C. Watts (2014). "Addressing violence against women: a call to action." The Lancet.
5. Mathews, S., R. Jewkes and **N. Abrahams** (2015). "'So now I'm the man': Intimate partner femicide and its interconnections with expressions of masculinities in South Africa." British Journal of Criminology **55**(1): 107-124
6. Shamu, S., C. Zarowsky, T. Shefer, M. Temmerman and **N. Abrahams** (2014). "Intimate partner violence after disclosure of HIV test results among pregnant women in Harare, Zimbabwe." PloS one **9**(10): e109447.
7. **Abrahams N,** Devries K, Watts C, et al. (2014). Worldwide prevalence of non-partner sexual violence: a systematic review. The Lancet; **383**(9929): 1648-54
8. van der Heijden, I. and N. Abrahams (2013). "Psychosocial group interventions for improving quality of life in adults living with HIV." The Cochrane Library.
9. **Abrahams, N**., S. Mathews, L. J. Martin, C. Lombard and R. Jewkes (2013). "Intimate Partner Femicide in South Africa in 1999 and 2009." PLoS Medicine **10**(4).
10. Devries, K. M., J. Y. T. Mak, C. García-Moreno, M. Petzold, J. C. Child, G. Falder, S. Lim, L. J. Bacchus, R. E. Engell, L. Rosenfeld, C. Pallitto, T. Vos, **N. Abrahams** and C. H. Watts (2013). "The Global Prevalence of Intimate Partner Violence Against Women." Science **340** (6140): 1527-1528
11. Stockl, H., K. Devries, A. Rotstein, **N. Abrahams**, J. Campbell, C. Watts and C. G. Moreno (2013). "The global prevalence of intimate partner homicide: a systematic review." Lancet, available online 20 June 2013.
12. Shamu, S., **N. Abrahams**, M. Temmerman and C. Zarowsky (2013). "Opportunities and obstacles to screening pregnant women for intimate partner violence during antenatal care in Zimbabwe." Culture, Health and Sexuality **15**(5): 511-524.
13. **Abrahams, N**. and R. Jewkes (2013). "Depressive symptomatology after a sexual assault: Understanding victim-perpetrator relationships and the role of social perceptions." African Journal of Psychaitry **16**(4): 288-293.
14. Mathews, S., **N. Abrahams**, R. Jewkes, L. J. Martin and C. Lombard (2013). "The Epidemiology of Child Homicides in South Africa." WHO Bulletin **91**: 562-568.
15. Mathews, S., **N. Abrahams** and R. Jewkes (2013). "Exploring Mental Health Adjustment of Children Post Sexual Assault in South Africa." Journal of Child Sexual Abuse **22**(6): 639-657.
16. Mathews, S., **N. Abrahams**, R. Jewkes and L. J. Martin (2013). "Underreporting child abuse deaths: Experiences from a national study on child homicide." South African Medical Journal **103**(3): 132-133.

17. Shamu, S., **N. Abrahams**, M. Temmerman, T. Shefer and C. Zarowsky (2012). ""That pregnancy can bring noise into the family": Exploring intimate partner sexual violence during pregnancy in the context of HIV in Zimbabwe." PLoS ONE **7**(8).
18. **Abrahams, N**. and R. Jewkes (2012). "Managing and resisting stigma: a qualitative study among people living with HIV in South Africa." J Int AIDS Soc **15**(2): 17330.
19. Shamu, S., N. Abrahams, M. Temmerman, A. Musekiwa and C. Zarowsky (2011). "A systematic review of African studies on intimate partner violence against pregnant women: Prevalence and risk factors." PLoS ONE **6**(3).
20. **Abrahams, N.**, R. Jewkes, L. J. Martin and S. Mathews (2011). "Forensic medicine in South Africa: Associations between medical practice and legal case progression and outcomes in female murders." PLoS ONE **6**(12).
21. Mathews, S., R. Jewkes and **N. Abrahams** (2011). "'I had a Hard Life': Exploring childhood adversity in the shaping of masculinities among men who killed an intimate partner in South Africa." British Journal of Criminology **51**(6): 960-977.
22. **Abrahams N** and R. Jewkes (2010). "'I believe I should not take the pills as people who are HIV positive take them': Barriers to Post Exposure Prophylaxis (PEP) Completion After Rape in South Africa." Cult Health Sex **12**: 471-484.
23. **Abrahams N,** Jewkes R, Lombard C, Mathews S, Campbell J and B. Meel (2010). "Impact of telephonic psycho-social support on adherence to post exposure prophylaxis (PEP) after rape: a randomised controlled trial." AIDS Care **16**: 1-9.
24. Norman, R., M. Schneider, D. Bradshaw, R. Jewkes, **N. Abrahams**, R. Matzopoulos and T. Vos (2010). "Interpersonal violence: An important risk factor for disease and injury in South Africa." Population Health Metrics **8**.
25. **Abrahams, N.,** R. Jewkes and S. Mathews (2010). "Guns and gender-based violence in South Africa." South African Medical Journal **100**(9): 586-588.
26. **Abrahams, N.,** R. Jewkes, L. J. Martin, S. Mathews, L. Vetten and C. Lombard (2009). "Mortality of women from intimate partner violence in South Africa: A national epidemiological study." Violence and Victims **24**(4): 546-556.
27. Mathews, S., **N. Abrahams**, R. Jewkes, L. J. Martin, C. Lombard and L. Vetten (2009). "Injury patterns of female homicide victims in South Africa." The Journal of trauma **67**(1): 168-172.
28. Mathews, S., **N. Abrahams**, R. Jewkes, L. J. Martin and C. Lombard (2009). "Alcohol use and its role in female homicides in the Western Cape, South Africa." Journal of studies on alcohol and drugs **70**(3): 321-327.
29. **Abrahams, N.,** L. J. Martin, R. Jewkes, S. Mathews, L. Vetten and C. Lombard (2008). "The epidemiology and the pathology of suspected rape homicide in South Africa." Forensic Science International **178**(2-3): 132-138.
30. **Abrahams, N**. and S. Mathews (2008). "Services for child sexual abuse lacking." S Afr Med J **98**(7): 494.
31. Campbell, J. C., **N. Abrahams** and L. Martin (2008). "Perpetration of violence against intimate partners: Health care implications from global data." CMAJ **179**(6): 511-512.
32. Mathews, S., **N. Abrahams**, R. Jewkes, L. J. Martin, C. Lombard and L. Vetten (2008). "Intimate femicide-suicide in South Africa: A cross-sectional study." Bulletin of the World Health Organization **86**(7): 552-558.
33. Norman, R., D. Bradshaw, M. Schneider, R. Jewkes, S. Mathews, **N. Abrahams**, R. Matzopoulos, T. Vos and G. South African Comparative Risk Assessment Collaborating (2007). "Estimating the burden of disease attributable to interpersonal violence in South Africa in 2000." S Afr Med J **97**(8 Pt 2): 653-656.
34. **Abrahams**, N., S. Mathews and P. Ramela (2006). "Intersections of 'sanitation, sexual coercion and girls' safety in schools'." Tropical Medicine and International Health **11**(5): 751-756.
35. Olley, B. O., N. **Abrahams** and D. J. Stein (2006). "Association between sexual violence and psychiatric morbidity among HIV positive women in South Africa." African journal of medicine and medical sciences **35 Suppl**: 143-147.
36. **Abrahams**, N., R. Jewkes, R. Laubscher and M. Hoffman (2006). "Intimate partner violence: Prevalence and risk factors for men in Cape Town, South Africa." Violence and Victims **21**(2): 247-263.

37. Outwater, A., N. **Abrahams** and J. C. Campbell (2005). "Women in South Africa: Intentional violence and HIV/AIDS: Intersections and prevention." Journal of Black Studies **35**(4): 135-154.
38. **Abrahams**, N. and R. Jewkes (2005). "Effects of South African men's having witnessed abuse of their mothers during childhood on their levels of violence in adulthood." American Journal of Public Health **95**(10): 1811-1816.
39. Christofides, N. J., R. K. Jewkes, N. Webster, L. Penn-Kekana, N. **Abrahams** and L. J. Martin (2005). ""Other patients are really in need of medical attention" - The quality of health services for rape survivors in South Africa." Bulletin of the World Health Organization **83**(7): 495-502.
40. Dyer, S. J., N. **Abrahams**, N. E. Mokoena, C. J. Lombard and Z. M. van der Spuy (2005). "Psychological distress among women suffering from couple infertility in South Africa: A quantitative assessment." Human Reproduction **20**(7): 1938-1943.
41. Dyer, S. J., N. **Abrahams**, N. E. Mokoena and Z. M. van der Spuy (2004). "'You are a man because you have children': Experiences, reproductive health knowledge and treatment-seeking behaviour among men suffering from couple infertility in South Africa." Human Reproduction **19**(4): 960-967.
42. **Abrahams**, N., R. Jewkes, M. Hoffman and R. Laubsher (2004). "Sexual violence against intimate partners in Cape Town: Prevalence and risk factors reported by men." Bulletin of the World Health Organization **82**(5): 330-337.
43. **Abrahams**, N., R. Adhikari, I. P. Bhagwat, N. Christofides, M. Djibuti, A. Dyalchand, G. Gotsadze, O. Grzmava, L. L. Huertas, T. Jacobs, R. Jewkes, N. Kapadia-Kundu, M. G. Karnikowski, S. Kimboka, A. Y. Kitua, J. U. Lens, A. Lopez, H. Lugina, L. Malecela, Y. Mashalla, A. Mishra, S. K. Mishra, R. Mlay, M. J. Moreno, S. Mpanda, F. Mwanga, G. Ndossi, G. Nigenda, A. Nkwera, O. T. Nobrega, S. K. Pahari, S. A. Paz, W. Phoolchareon, P. Ramachandran, R. P. Rannan-Eliya, K. G. Rodrigues, A. Salazar, P. S. Sarma, J. Shija, L. D. Silver, P. Tatsanavivat, K. R. Thankappan, A. J. Tuesta, O. Vasadze, A. C. Velez, N. Webster and C. A. Yesudian (2004). "Changing the debate about health research for development. International Health Research Awards Recipients." Journal of public health policy **25**(3-4): 259-287.
44. **Abrahams**, N., R. Jewkes and Z. Mvo (2002). "Indigenous healing practices and self-medication amongst pregnant women in Cape Town, South Africa." African Journal of Reproductive Health **6**(2): 79-86.
45. Dyer, S. J., N. **Abrahams**, M. Hoffman and Z. M. Van Der Spuy (2002). "Infertility in South Africa: Women's reproductive health knowledge and treatment-seeking behaviour for involuntary childlessness." Human Reproduction **17**(6): 1657-1662.
46. Dyer, S. J., N. **Abrahams**, M. Hoffman and Z. M. Van Der Spuy (2002). "'Men leave me as I cannot have children': Women's experiences with involuntary childlessness." Human Reproduction **17**(6): 1663-1668.
47. Jewkes, R. and N. **Abrahams** (2002). "The epidemiology of rape and sexual coercion in South Africa: An overview." Social Science and Medicine **55**(7): 1231-1244.
48. **Abrahams**, N., R. Jewkes and Z. Mvo (2001). "Health care-seeking practices of pregnant women and the role of the midwife in Cape town, South Africa." Journal of Midwifery and Women's Health **46**(4): 240-247.
49. Jewkes, R., C. Watts, N. **Abrahams**, L. Penn-Kekana and C. García-Moreno (2000). "Ethical and methodological issues in conducting research on gender-based violence in Southern Africa." Reproductive Health Matters **8**(15): 93-103.
50. **Abrahams**, N. (2000). "Research on rape survivors - what is wrong with a gender analysis?" Agenda(46): 71-74.
51. **Abrahams**, N. and R. Jewkes (1998). "Men on Violence Against Women." Psychology Bulletin **8**(2): 30-34.
52. Jewkes, R., N. **Abrahams** and Z. Mvo (1998). "Why do nurses abuse patients? Reflections from South African obstetric services." Social Science and Medicine **47**(11): 1781-1795.
53. **Abrahams**, N., K. Wood and R. Jewkes (1997). "Barriers to cervical screening: women's and health workers' perceptions." Curationis **20**(1): 50-52.
54. Wood, K., R. Jewkes and N. **Abrahams** (1997). "Cleaning the womb: Constructions of cervical screening and womb cancer among rural black women in South Africa." Social Science and Medicine **45**(2): 283-29.

CONFERENCE PRESENTATIONS (last 3 years only (2010-2014)-more than 100 oral conference presentations

<u>2014</u>

Abrahams N. Overview of the Intersection of HIV and Violence. 9th Workshop of HIV Transmission-Satellite Session October 25th, 2014 Cape Town, South Africa

Abrahams N. *The Role of Schools and the Education Sector in Sexual Violence Prevention*

IATT 2014 SYMPOSIUM. School of Public Health, University of the Western Cape, Cape Town, South Africa 23 to 25 February 2014

Abrahams N . Child homicide in South Africa. KNOW Violence in Childhood Initiative. Launch and Meeting 24-25 November 2014 New Delhi India

<u>2013</u>

Abrahams N, Devries K, Watts C, Pallitto C, Petzold M, Sham S, Garcia-Moreno C. **(2013).** Global prevalence of non-partner sexual violence. Paper presented at the Sexual Violence Research Initiative (SVRI) Forum, 14-17 October, Royal Orchid Sheraton Hotel and Towers, Bangkok, Thailand

Abrahams N (2013). Evidence of the linkages between GBV and HIV. Round Table discussion on HIV and GBV. 9th December 2013 . 17th ICASA Cape Town 7-11 December 2013.Cape Town, South Africa (I**nvited**)

Abrahams N. (2013*). Defining and Conceptualizing Femicide: Observations from the South African case study*. ECost Meeting, European Cooperation in Science and Technology. Cost Action 1206 Femicide across Europe. Jerusalem Israel Oct 24 2013**. (Invited)**

Abrahams N. (2013). *Gender based Violence in South Africa: Exploring the role of gun-violence in South Africa* Global Health Institute. George Washington University 23 May 2013. Washington USA. (**Invited**)

Abrahams N. (2013). *The Role of Alcohol in Gender based Violence.* Alcohol and Violence workshop: University of Cape Town. 21 March 2013 Cape Town, South Africa. (**Invited**)

Simukai Shamu**,** Christina Zarowsky, Tamara Shefer, Marleen Temmerman, **Naeemah Abrahams**. Intimate partner violence after disclosure of HIV test results among pregnant women in Harare, Zimbabwe. Paper presented at the Sexual Violence Research Initiative (SVRI) Forum, 14-17 October, Royal Orchid Sheraton Hotel and Towers, Bangkok, Thailand

Simukai Shamu**,** Christina Zarowsky, Kristien Roelens, Marleen Temmerman, **Naeemah Abrahams**. Intimate partner violence during pregnancy and maternal and child health outcomes in Harare, Zimbabwe. Paper presented at the Sexual Violence Research Initiative (SVRI) Forum, 14-17 October, Royal Orchid Sheraton Hotel and Towers, Bangkok, Thailand

Simukai Shamu, Christina Zarowsky, Tamara Shefer, Marleen Temmerman , **Naeemah Abrahams**. "The Dynamics of Intimate Partner Violence during pregnancy and linkages with HIV infection and disclosure in Zimbabwe". Second Annual UWC SOPH Graduates' Research and Networking Day, March 18, 2013, School of Public Health, University of the Western Cape. South Africa

<u>2012</u>

Abrahams N, Mathews S, & Jewkes R, Martin L & Lombard C. (2012). *Intimate Femicide In South Africa: Comparing Two Studies 10 Years Apart.* 11th World Conference on Injury Prevention and Safety Promotion. 1-3 October 2012 New Zealand.

Mathews S, **Abrahams N** & Jewkes R, Martin L & Lombard C. (2012). *Patterns of Child Homicide in South Africa*. SAPSAC Conference 25th – 27th June 2012 Pretoria, South Africa.

Mathews S, **Abrahams N** & Jewkes R, Martin L & Lombard C (2012). *The epidemiology of child homicide in South Africa: is there a link to child abuse*? ISPCAN Conference Istanbul 9th -12th September 2012, Istanbul Turkey.

Gevers, A., **Abrahams, N**. & Jewkes, R. (2012). *Whose mental health and well-being are we promoting? The need to promote mental health among service providers in primary care sexual assault services in South Africa.* Oral presentation at the 7th World Conference on the Promotion of Mental Health and the Prevention of Mental and Behavioural Disorders, Perth, Australia.

Abrahams N. (2012) Gender based violence in South Africa. Presentation to the Auckland University New Zealand. 4th October 2012. Auckland, New Zealand.

Abrahams N. Devries K, Watts C & Garcia-Morena C . (2012). *Review of the linkages on Gender based violence and HIV*. On behalf of the Global Burden of Disease study Injuries and Risk Factor Studies Expert Group on Interpersonal Violence. Gender Based Violence and HIV Workshop. Nairobi 30 July 2012. Nairobi Kenya.

TEACHING/TRAINING

- Gender based violence module for the Gender & Health Course for MPH students, UCT
- Gender based violence for 4th Year medical students doing Family Medicine Module, UCT
- Teaching on the training program of African Sexuality Resource Centre
- Co-facilitate 2 week course with PATH, Liverpool VCT, Care & Treatment KENYA, Addis Continental Institute of Public Health, Intercambios Nicaragua. Raising Voices Uganda, Research training of gender based violence in Africa. (South Africa 2005, Kenya 2007, Tanzania 2008)
- Co-facilitate Monitoring and Evaluation 1 week course for prevention of gender based violence programmes for UN Trust Fund grantees in Asia
- Qualitative methods on the Reproductive Health Research Methods course
- Gender and Health module on the UWC Masters in Gender Studies course

POST GRADUATE SUPERVISION

Master Students : Completed (6)

- Shanaaz Mathews: Masters in Public Health, UCT, graduated in 2006
- Nelisiwe Khuzwayo: Master in Health Promotion, University of KZN, graduated 2008
- Lydia Shilongo: Master in Public Health, UWC, graduated in 2010
- Adefolalu Adegoke Olusegun Master in Public Health, UWC, graduated in 2010
- Steve Mashele: Master Nursing Science, University of Stellenbosch, graduated 2012
- Kaala Moomba: Master in Public Health, UWC, Graduated 2012

Ph.D Students Completed (2)

- Shanaaz Mathews PHD Public Health University of Wit Waters Rand 2010
- Simukai Shamu PHD Public Health University of Western Graduated 2013

<u>**Current Students**</u>

Masters students

- Catherine Nguni, MPH UWC

PHD Students

- Tania De Villiers: PhD Nursing Science UCT
- Ingrid van Der Heijden –PhD Public Health UCT
- Bianca – PhD Psychology Dept. UWC

INTERNATIONAL POSITIONS

- Technical Advisor to the WHO Multi-Country Study on Domestic Violence and Women Health in Namibia.
- Foreign Faculty member of Johns Hopkins School of Nursing, Baltimore
- Teaching on the African Sexuality Resource Course
- Collaboration with PATH, Liverpool VCT, Care & Treatment KENYA, Addis Continental Institute of Public Health, Intercambios Nicaragua and Raising Voices Uganda, Research training of gender based violence research in Africa. (South Africa 2005, Kenya 2007, Tanzania 2008, Addis Ababa 2009)
- Collaboration with WHO, PATH, Intercambios to develop material to strengthen the global research on femicide. Writing of a technical report as an addendum to the Violence against women research training material

- Member of Project Advisory Board for the study for NIH/women's health UCT study: A Structural Intervention to Integrate Reproductive Health Care with HIV/AIDS Care in the Western Cape, South Africa.
- Member of Expert Advisory group for the study on adapting an evidence-based HIV prevention intervention to out-of-school pregnant and postpartum adolescents in Cape Town
- Collaboration with LSHTM and a member of the research team of the Estimates for the Global Burden of Disease Project 2010: Intimate Partner Violence, Childhood Sexual Abuse and Non-Partner Sexual Violence
- Senior technical advisor to the Wellsprings ICRW project for building GBV research capacity in Africa – Rwanda, Tanzania, Uganda, Malawi
- Appointed member of the Advisory Group to the Gender Based Violence Research Exchange of the Global Women's Institute of George Washington University 2013
- Invited as an author Lancet Series on VAW – Publication in November 2014
- Member of team on Global study on Child Homicide (LSHTM & WHO)

AWARDS

Finalist in the NRF Black Scientist of the Year award - 2005

Awarded MRC Flagship Award for Rape Impact Cohort Study (RICE) - Feb 2014 – Jan 2018

Silver Medalist for the MRC Scientific Merit awards – October 2013

RESEARCH FUNDING

Oct 2014- August 2015

Children's Fund - UNICEF

Grant Number: LRPS-PMB-2014-9114355

Surveillance system for Violence against Women and Children in South Africa

R *Role:* Principal Investigator

R 780 636

July 2014 – June 2019

CDC-MRC Cooperative Agreement

Grant number: 1U2GGH001150-01

The National Gender Based Violence Surveillance System Study

R *Role:* Principal Investigator

$ 2 250000 (450 000 per year for 5 years)

Feb 2014- Jan 2017: (PI: N Abrahams)

The MRC Flagship Award MRC-RFA-IFSP-01-2013 :

Rape Impact Cohort Study (RICE)

Role: Principal Investigator

R 8 250 000

Feb 2011- Jan 2012 **(PI: N Abrahams)**

CDC Grant Number U2G/PS001137/ 01/ 02:

U.S. President's Emergency Plan for AIDS Relief (PEPFAR) for HIV and AIDS prevention,

care and treatment. Cooperative Agreement to the Medical Research Council of South Africa

Capacity Assessment for Mental Health Services for Rape Victims in Primary Health Care'

Role: Principal Investigator

R 522 840

Feb 2012- Jan 2013 (PI: N Abrahams)

CDC Grant Number U2G/PS001137/ 03/ 04:

Developing and pilot testing a gender-based HIV AIDS risk reduction and coping skills intervention for HIV positive men and women in South Africa'.

Role: Principal Investigator

R2 930 951

COMPLETED

April 2010-March 2012 (PI: N Abrahams)

Grant: Open Society Foundation :

National study of Female and Child Homicide in South Africa

Role: Principal Investigator

R 510 000

Sep 2006-Dec 2007

Grant: IRISH AID (PI: N Abrahams)

Testing an intervention to improve Post Exposure Prophylaxis adherence.

Description: RCT of a post-rape PEP adherence intervention

Role: Principal Investigator

R 456 465

____

Signed

Appendix I: Prof Jewkes

CV FORMAT FOR HEALTH PROFESSIONALS

Trial title: **A national study of injury-related mortality, with a focus on homicide in South Africa**

Protocol no: /

CURRICULUM VITAE

Prof Rachel JEWKES, MBBS MSc(distin.) MFPHM MD
NRF rating: A1

Present position: *Director*, Gender and Health Research Unit, Medical Research Council,
Director of What Works To Prevent Violence: A global programme to prevent violence against women and girls
Secretary of the Sexual Violence Research Initiative

Formerly: *Vice-President (Research Support)*, Medical Research Council, acting position from Feb 2013-September 2014

Address: Gender & Health Research Unit, Medical Research Council, Private Bag X385, Pretoria 0001

Phone & Fax: 012-339-8525 (w) 012-339-8582 (fax) email: rjewkes@mrc.ac.za

QUALIFICATIONS:

Accredited Specialist in Public Health Medicine March 1995
(UK General Medical Council)

Doctor of Medicine (MD) December 1994
London School of Hygiene and Tropical Medicine (LSHTM)
MD Thesis: 'Meanings of community in community participation in health promotion'

Membership of the Faculty of Public Health Medicine (MFPHM) June 1993
London, UK.

MSc Community Medicine (distinction) September 1991
London School of Hygiene and Tropical Medicine

Bachelor of Medicine, Bachelor of Surgery (MBBS) June 1986
St Thomas's Hospital Medical School, University of London.

AWARDS, PRIZES AND HONOURS

NRF Rating 2016 **A1**
NRF Rating 2011 **A2**

EMPLOYMENT:

February 2013- acting Vice-president (Research Support) MRC

January 2004- Director, Gender & Health Research Unit, MRC

January 2001 – 2003 Director, Gender & Health Group**,** Medical Research Council, Pretoria**.**

A sample of publications in peer reviewed journals

1. Christofides N, Jewkes R, Dunkle K, Nduna M, Jama-Shai N, Sterk C. Early pregnancy increases risk of incident HIV infection in the Eastern Cape, South Africa: a prospective study. Journal of the International AIDS Society. 2014 Mar 19;17(1):18585. doi: 10.7448/IAS.17.1.18585. eCollection 2014.

2. Gibbs A, Sikweyiya Y, Jewkes R. (2014) "Men value their dignity": securing respect and identity construction in urban informal settlements in South Africa. Global Health Action 7, 23676.

3. Gibbs A, Jewkes R, Mbatha N, Washington L, Willan S. (2014) Jobs, food, taxis and journals: complexities of implementing Stepping Stones and Creating Futures in urban informal settlements in South Africa. AJAR 13(2):161-7. doi: 10.2989/16085906.2014.927777.

4. Gibbs, Jewkes R, Sikweyiya Y, Willan S. (2015) Reconstructing Masculinity? A qualitative evaluation of the Stepping Stones and Creating Futures intervention in urban informal settlements in South Africa. Culture, Health & Sexuality 17(2):208-22 http://dx.doi.org/10.1080/13691058.2014.966150.

5. Christofides NJ, Jewkes RK, Dunkle KL, McCarty F, Jama Shai N, Nduna M, Sterk CE. (2014) Risk factors for unplanned and unwanted teenage pregnancies over two years of follow up among a cohort of young South African women Global Health Action 7:23719 http://dx.doi.org/10.3402/gha.v7.23719

6. Christofides N, Jewkes R, Dunkle K, Nduna M, Jama-Shai N. (2014) Perpetration of physical and sexual abuse and subsequent fathering of pregnancies among a cohort of young South African men. BMC Public Health 14:947. doi: 10.1186/1471-2458-14-947.

7. Jewkes R, Sikweyiya Y, Jama-Shai N (2014) The challenges of research on violence in post-conflict Bougainville. The Lancet 383(9934):2039-40. http://dx.doi.org/10.1016/S0140-6736(14)60969-7

8. Jewkes R, Gibbs A, Jama-Shai N, Willan S, Misselhorn S, Mushinga M, Washington L, Mbatha N, Sikweyiya Y. (2014) Stepping Stones and Creating Futures Intervention: Outcomes of a shortened interrupted time series evaluation of behavioural and structural intervention for young people in informal settlements in Durban, South Africa BMC Public Health 14:1325 DOI 10.1186/1471-2458-14-1325

9. Sikweyiya Y, Dunkle K, Jewkes R (2014) Impact of HIV on and the constructions of masculinities among HIV positive men in South Africa: Implications for secondary prevention programs. Global Health Action **7**: 24631 - http://dx.doi.org/10.3402/gha.v7.24631

10. Jewkes R, Flood M, Lang J (2015) From working with men and boys to changing social norms and reducing inequities in gender relations: a paradigm shift in prevention of violence against women and girls. The Lancet 385(9977):1580-9.
http://dx.doi.org/10.1016/ S0140-6736(14)61683-4

11. Mathews S, Jewkes R, Abrahams N. (2015) "So now I'm the man": Intimate partner femicide and its interconnections with expressions of masculinities in South Africa. British Journal of Criminology. 55 (1): 107-124. doi:10.1093/bjc/azu076
12. Jewkes R (2014) (How) Can we reduce gender-based violence by 50% over the next 30 years? Plos Medicine 11(11): e1001761. doi:10.1371/journal.pmed.1001761
13. Jewkes R. (2015) SHARE: a milestone in joint programming for HIV and intimate partner violence. The Lancet Global Health Jan;3(1):e2-3 doi: 10.1016/S2214-109X(14)70374-2
14. Jina R, Jewkes R, Vetten L, Christofides N, Sigsworth R, Loots L (2015) Genito-anal injury patterns and associated factors in rape survivors in an urban province of South Africa: a cross-sectional study. BMC Public Health 15: 29. DOI 10.1186/s12905-015-0187-0
15. Jewkes R, Dunkle K, Jama-Shai N, Gray G. (2015) Impact of exposure to intimate partner violence on CD4+ and CD8+ T cell decay in HIV infected women: longitudinal study. Plos One. 2015 Mar 27;10(3):e0122001. doi: 10.1371/journal.pone.0122001. eCollection 2015.
16. Jewkes R, Sikweyiya Y, Dunkle K, Morrell R. (2015) Relationship between single and multiple perpetrator rape perpetration in South Africa: A comparison of risk factors in a population-based sample. BMC Public Health (2015) 15:616 DOI 10.1186/s12889-015-1889-9
17. Jewkes R, Penn-Kekana L (2015) Mistreatment of women in child birth: time for action on this important dimension of violence against women. Plos Medicine. 12(6): e1001849.

doi:10.1371/journal.pmed.1001849
18. Jewkes R, Morrell R, Hearn J, Lundqvist E, Blackbeard D, Lindegger G, Quayle M, Sikweyiya Y, Gottzen L (2015) Hegemonic masculinity: combining theory and practice in gender interventions. Culture Health and Sexuality. 17:sup2, 96-111, DOI: 10.1080/13691058.2015.1085094
19. Gibbs A, Willan S, Jama-Shai N, Washington L, Jewkes R "Eh! I felt I was sabotaged!": Facilitators understandings of success in a participatory HIV and IPV prevention intervention in urban South Africa Health Education Research 2015 30 (6): 985-995 doi: 10.1093/her/cyv059
20. Shamu S, Gevers A, Mahlangu P, Jama-Shai N, Chirwa E, Jewkes R (2015) Prevalence and risk factors for intimate partner violence among Grade 8 learners in urban South Africa: baseline analysis from the Skhokho Supporting Success cluster randomised controlled trial. International Health doi:10.1093/inthealth/ihv068
21. Jina R, Jewkes R, Christofides N, Loots L. 2014. A cross-sectional study on the effect of post-rape training on knowledge and confidence of health professionals in South Africa. Int J Gynaecol Obstet., 126(2):187-92.
22. Machisa M, Christofides N, Jewkes R (2016) Structural Pathways between Child Abuse, Poor Mental Health Outcomes and Male-Perpetrated Intimate Partner Violence (IPV). PLoS ONE 11(3):e0150986. doi:10.1371/journal.pone.0150986
23. Abrahams N, Mathews S, Martin LJ, Lombard C, Nannan N, Jewkes R. (2016) Gender differences in homicide of neonates, infants and under-five year olds in South Africa: Results from the 2009 national child homicide study Plos Medicine 13(4): e1002003. doi:10.1371/journal.pmed.1002003
24. Jewkes R (2016) Violence against women must concern obstetrician–gynaecologists. International Journal of Gynaecology and Obstetrics 133, 1-2. http://dx.doi.org/10.1016/j.ijgo.2016.01.003
25. Jewkes R, Nduna M, Jama-Shai N, Chirwa E, Dunkle K (2016) Understanding the relationships between gender inequitable behaviours, childhood trauma and socio-economic status in single and multiple perpetrator rape in rural South Africa: structural equation modelling. Plos One 11(5):e0154903. doi:10.1371/journal.pone.0154903
26. Gibbs A, Govender K, Jewkes R (2016) An Exploratory Analysis of Factors Associated with Depression in a Vulnerable Group of Young People Living in informal settlements in South Africa. Global Public Health http://dx.doi.org/10.1080/17441692.2016.1214281
27. Gibbs A, Sikweyiya Y, Jewkes R (2016) "I tried to resist and avoid bad friends": The role of social contexts in shaping the transformation of masculinities in a gender-transformative and livelihood strengthening intervention in South Africa. Men and Masculinities DOI: 10.1177/1097184X17696173 | First Published January 1, 2017
28. Fulu E, Miedema S, Roselli T, Ko Ling Chan E, Jewkes R. Pathways between violence against children and intimate partner violence and child maltreatment: Findings from the cross-sectional UN Multi-country Study on Men and Violence in Asia and the Pacific. Lancet Global Health (accepted)
29. Jewkes R, Fulu E, Tabassam Naved R, Chirwa E, Dunkle K, Haardoerfer R, Garcia-Moreno C. Comparing reports of past year prevalence of intimate partner violence and rape from women and men, and women's risk factors for IPV: findings from the UN Multi-country Cross–sectional Study on Men and Violence in Asia and the Pacific. Plos Medicine (submitted)
30. Morrell R, Dunkle K, Ibragimov U, Jewkes R (2016) Fathers who care and those that don't: Men and childcare in South Africa. South Africa Journal of Sociology http://dx.doi.org/10.1080/21528586.2016.1204240
31. Jewkes R, Morrell R. Hegemonic Masculinity, Violence and Gender Equality: using latent class analysis to investigate the origins and correlates of differences between men. Men and Masculinities **Article first published online:** March 15, 2017 DOI: https://doi.org/10.1177/1097184X1769617
32. McFarlane J, Karmaliani R, Khuwaja H, Gulzar S, Somani R, Saeed Ali T, Somani YH, Bhamani SS, Krone RD, Paullson RM, Muhammed A, Jewkes R. Preventing Violence Against Children: Methods and Baseline Data of a Cluster Randomized Controlled Trial in Pakistan. Global Health: Science and Practice (in press)
33. Jewkes R, Jama-Shai N, Sikweyiya Y. Enduring impact of conflict on mental health and gender-based violence perpetration in Bougainville, Papua New Guinea: a cross-sectional study. (submitted)
34. Abrahams N, Mathews S, Lombard C, Martin LJ, Jewkes R. Sexual homicides in South Africa: a national cross-sectional epidemiological study of adult women and children. Forensic Science International (submitted)
35. Jama Shai N, Van Der Heijden I, Sikweyiya Y, Abrahams N, Jewkes R. 'I was in the darkness but the group brought me light': Development, Acceptability and Feasibility of the Sondela HIV Adjustment and Coping Intervention PLOS ONE (under review)
36. Machisa MT, Christofides N, Jewkes R. Mental ill health in structural pathways to women's experiences of intimate partner violence . Plos ONE (under review)
37. Coetzee J, Gray G, Jewkes R. Cross-sectional study of female sex workers in Soweto, South Africa: factors associated with HIV infection Plos ONE (under review)
38. Tazeen Saeed Ali, Rozina Karmaliani, Judith Mcfarlane, Yasmin Somani, Hussain MA Khuwaja,

Esnat D. Chirwa, Rachel Jewkes Preventing Violence Against Women by Changing Youth Attitudes Toward Gender Roles: Baseline Findings from an RCT of 1,752 Youth in Pakistan Global Health Action

39. Hussain MA Khuwaja, Rozina Karmaliani, Judith Mcfarlane, Rozina Somani, Saleema Gulzar, Tazeen Saeed Ali, Zahra Shaheen, Esnat D. Chirwa, Rachel Jewkes The Intersection of School Corporal Punishment and Youth Peer Violence: baseline results From a Randomized Controlled Trial in Pakistan. Child Abuse & Neglect

40. Rozina Karmaliani, Judith Mcfarlane, Hussain MA Khuwaja, Saleema Gulzar, Rozina Somani, Tazeen Saeed Ali, Zahra Shaheen, Rachel Jewkes Victimization And Perpetration Of Youth: Perceptions And Experiences Among Six To Eight Grade Students And Teachers In Pakistan Journal of Adolescent Research

41. Rozina Karmaliani, Judith Mcfarlane, Rozina Somani, Hussain MA Khuwaja, Shireen Shehzad Bhamani,Tazeen Saeed Ali, Saleema Gulzar, Yasmeen Somani, Esnat D. Chirwa, Rachel Jewkes. Peer violence perpetration and victimization: prevalence, associated factors and pathways among 1752 sixth grade boys and girls in schools in Pakistan PloS ONE

42. Nargis Asad, Rozina Karmaliani, Judith McFarlane, Shireen Shehzad Bhamani, Yasmeen Somani, Esnat D. Chirwa, Rachel Jewkes The Intersection of Adolescent Depression and Peer Violence: Baseline Results from A Randomized Controlled Trial of 1,752 Youth in Pakistan. Journal of Child and Adolescent Mental health

Appendix J: Prof. Martin

CV FORMAT FOR HEALTH PROFESSIONALS

Trial title: A national study of male, female and child homicide in South Africa

Protocol no: /

Designation:

1. Personal details

Name	**Lorna Jean Martin**	Tel no.	
Address	**Division of Forensic Medicine & Toxicology,**	Work tel no.	**0214066110**
	**Department of Pathology**	Fax no.	**0214481249**
	**University of Cape Town**	Cell phone no	**0826006350**
		Email	**Lornaj.martin@uct.ac.za**

2. Academic and professional qualifications

[illegible]

INSTITUTION AND LOCATION	DEGREE *(if applicable)*	Completion Date MM/YYYY	FIELD OF STUDY
Colleges of Medicine of South Africa	Fellowship For Path.	2011	Forensic Pathology
Colleges of Medicine of South Africa	Associate F For Path.	2002	Forensic Pathology
University of Cape Town	M Med Path (Foren).	1999	Forensic Pathology
Colleges of Medicine of South Africa	Dip For Med.	1992	Forensic Pathology
University of the Witwatersrand	MB.BCh.	1989	Medicine

3. Health Professions Council of South Africa (HPCSA) registration number if applicable (or other health professions body registration particulars if applicable – e.g. Nursing Council)

HPCSA MP0347850

4. Current personal medical malpractice insurance details [medical and dental practitioners] (please put MRC, not N/A, if you do not have other insurance)

Medical Protection Society

5. Relevant related work experience (brief) and current position

2.1 PROFESSOR AND HEAD OF DIVISION: DIVISION OF FORENSIC MEDICINE, UNIVERSITY OF CAPE TOWN; & HEAD: CLINICAL DEPARTMENT WCG HEALTH

UNIVERSITY OF CAPE TOWN & WESTERN CAPE GOVERNMENT: DEPARTMENT OF HEALTH
1^{ST}SEPTEMBER 2004 – PRESENT,

Inaugural: *"No woman No Cry" – a journey into forensic pathology services and violence against women in South Africa, 5 October 2011*

2.2 HEAD OF DEPARTMENT

DEPARTMENT OF CLINICAL LABORATORY SCIENCES, UNIVERSITY OF CAPE TOWN
1ST SEPTEMBER 2007 – 31st December 2013,

2.3 REGISTRAR (JULY 1996– JUNE 1999)

SPECIALIST (JULY 1999–AUGUST 2004)

WESTERN CAPE GOVERNMENT: DEPARTMENT OF HEALTH/ DIVISION OF FORENSIC MEDICINE, UNIVERSITY OF CAPE TOWN
1ST JULY 1996 – 31ST AUGUST 2004,

2.4 REGISTRAR

GAUTENG: DEPARTMENT OF HEALTH/ DEPARTMENT OF FORENSIC PATHOLOGY, UNIVERSITY OF THE WITWATERSRAND.
1^{ST}JANUARY 1995 – 30 JUNE 1996,

2.5 MEDICAL OFFICER (FULL-TIME) 1/01/91– 31/12/92

SENIOR MEDICAL OFFICER (FULL-TIME) 1/01/93– 31/12/94

SENIOR MEDICAL OFFICER (PART-TIME) 1/01/95– 30/06/96

GAUTENG: DEPARTMENT OF HEALTH, DISTRICT SURGEON'S OFFICE, JOHANNESBURG
1ST JANUARY 1991 TO 30TH JUNE 1996

2.6 INTERN

DEPARTMENT OF HEALTH, TRANSVAAL: BARAGWANATH HOSPITAL, SOWETO
1ST JANUARY 1990 TO 31ST DECEMBER 1990

8. Date of last GCP training (as a participant or presenter)
2004

9. Any additional relevant information supporting abilities to participate in conducting this research. [Briefly]

NAME IN FULL: **Lorna Jean Martin**

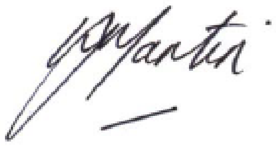

17th January 2018

Signature: **Date:**

Appendix K: Prof. Lombard

CV FORMAT FOR HEALTH PROFESSIONALS

Trial title: **A national study of injury-related mortality, with a focus on homicide in South Africa**

Protocol no: /

Designation:

1. Personal details

Name	**Carl Lombard**	Tel no.	**0219380924**
Address	**Biostatistics Unit**	Work tel no.	**0219380924**
	**SAMRC**	Fax no.	
	**Cape Town**	Cell phone no	**0824114323**
		Email	**carl.lombard@mrc.ac.za**

2. Academic and professional qualifications

Degree	Field of study	University	Year
**MSc**	**Statistics**	**University of the Free State**	**1976**
**PhD**	**Statistics**	**University of the Free State**	**1981**

3. Health Professions Council of South Africa (HPCSA) registration number if applicable (or other health professions body registration particulars if applicable – e.g. Nursing Council)

4. Current personal medical malpractice insurance details [medical and dental practitioners] (please put MRC, not N/A, if you do not have other insurance)

MRC

5. Relevant related work experience (brief) and current position

Period	Position	Employer
**1977-1982**	**Lecturer/Senior lecturer**	**University of the Free State**
**1983-2000**	**Specialist Statistician**	**SAMRC**
**2001-2016**	**Director Biostats Unit**	**SAMRC**
**2016-**	**Specialist Statistician**	**SAMRC**

6. Participation in clinical trials research in the last three years (title, protocol number, designation) [If multiple trials, only list those with relevance to this application, or in the last year.]

R01 2014-2017 COBALT: Comorbid Affective Disorders, AIDS/HIV, and Long Term Health. National Institute of Mental Health (NIMH): PI's Thornicroft G, Petersen I, Fairall L. COBALT is a pragmatic cluster randomized controlled trial in public sector primary care clinics in the North West province of South Africa. The trial will evaluate the effectiveness of a facility-based intervention combining depression case detection by non-physician clinicians with individual and group psychosocial counselling for depression delivered by lay-health workers, on mental health and HIV outcomes in depressed adults receiving antiretroviral treatment (ART).
Role: Co-investigator/study statistician

MRC/DFID/Welcome Trust 2015-2018
Strengthening South Africa's health system through integrating treatment for mental illness into chronic disease care (Project MIND). To assess the feasibility and acceptability of a vertical and horizontal model for the integrating mental health care in to HIV and diabetes services and to compare the effectiveness of these two options relative to treatment as usual. PI: Myers B. (SAMRC) Co-PI Sorsdal K.
Role: Co-investigator/study statistician

CIHR/IPPH/IDRC/SAMRC 2015-2018
IINDIAGO:Integrated Intervention for Diabetes risk after Gestational diabetes. An integrated health systems intervention aimed at redcuing type 2 diabetes risk in disadvantaged women after gestational diabetes in South Africa. PI: Levitt N (UCT)

Role : Co-investigator/ study statistician

7. Peer-reviewed publications in the past 3 years

1. Uptake and predictors of early postnatal follow-up care amongst mother-baby pairs in South Africa: Results from three population-based surveys, 2010-2013.

   Larsen A, Cheyip M, Aynalem G, Dinh TH, Jackson D, Ngandu N, Chirinda W, Mogashoa M, Kindra G, Lombard C, Goga A.

   J Glob Health. 2017 Dec;7(2):021001. doi: 10.7189/jogh.07.021001.

   PMID: 29302327 [PubMed - in process] **Free PMC Article**

   Similar articles

2. Adolescent Access to Care and Risk of Early Mother-to-Child HIV Transmission.

   Ramraj T, Jackson D, Dinh TH, Olorunju S, Lombard C, Sherman G, Puren A, Ramokolo V, Noveve N, Singh Y, Magasana V, Bhardwaj S, Cheyip M, Mogashoa M, Pillay Y, Goga AE.

   J Adolesc Health. 2017 Dec 19. pii: S1054-139X(17)30504-9. doi: 10.1016/j.jadohealth.2017.10.007. [Epub ahead of print]

   PMID: 29269045 [PubMed - as supplied by publisher] **Free Article**

   Similar articles

3. Correction to: Effects of early feeding on growth velocity and overweight/obesity in a cohort of HIV unexposed South African infants and children.

   Ramokolo V, Lombard C, Chhagan M, Engebretsen IMS, Doherty T, Goga AE, Fadnes LT, Zembe W, Jackson DJ, Van den Broeck J.

   Int Breastfeed J. 2017 Nov 13;12:47. doi: 10.1186/s13006-017-0138-5. eCollection 2017.

   PMID: 29158772 [PubMed - in process] **Free PMC Article**

   Similar articles

4. Evaluation of a Mass-Media Campaign to Increase the Awareness of the Need to Reduce Discretionary Salt Use in the South African Population.

   Wentzel-Viljoen E, Steyn K, Lombard C, De Villiers A, Charlton K, Frielinghaus S, Crickmore C, Mungal-Singh V.

   Nutrients. 2017 Nov 12;9(11). pii: E1238. doi: 10.3390/nu9111238.

   PMID: 29137143 [PubMed - in process] **Free PMC Article**

   Similar articles

5. Maternal cardiac output response to colloid preload and vasopressor therapy during spinal anaesthesia for caesarean section in patients with severe pre-eclampsia: a randomised, controlled trial.

   Dyer RA, Daniels A, Vorster A, Emmanuel A, Arcache MJ, Schulein S, Reed AR, Lombard CJ, James MF, van Dyk D.

   Anaesthesia. 2018 Jan;73(1):23-31. doi: 10.1111/anae.14040. Epub 2017 Oct 31.

   PMID: 29086911 [PubMed - in process]

   Similar articles

6. In Utero ART Exposure and Birth and Early Growth Outcomes Among HIV-Exposed Uninfected Infants Attending Immunization Services: Results From National PMTCT Surveillance, South Africa.

   Ramokolo V, Goga AE, Lombard C, Doherty T, Jackson DJ, Engebretsen IM.

   Open Forum Infect Dis. 2017 Aug 30;4(4):ofx187. doi: 10.1093/ofid/ofx187. eCollection 2017 Fall.

   PMID: 29062860 [PubMed] **Free PMC Article**

   Similar articles

7. Sexual homicides in South Africa: A national cross-sectional epidemiological study of adult women and children.

   Abrahams N, Mathews S, Lombard C, Martin LJ, Jewkes R.

   PLoS One. 2017 Oct 17;12(10):e0186432. doi: 10.1371/journal.pone.0186432. eCollection 2017.

PMID: 29040329 [PubMed - indexed for MEDLINE] **Free PMC Article**

Similar articles

8. Study protocol for a longitudinal study evaluating the impact of rape on women's health and their use of health services in South Africa.

   Abrahams N, Seedat S, Lombard C, Kengne AP, Myers B, Sewnath A, Mhlongo S, Ramjee G, Peer N, Garcia-Moreno C, Jewkes R.

   BMJ Open. 2017 Sep 29;7(9):e017296. doi: 10.1136/bmjopen-2017-017296.

   PMID: 28965098 [PubMed - in process] **Free PMC Article**

   Similar articles

9. A randomised comparison of bolus phenylephrine and ephedrine for the management of spinal hypotension in patients with severe preeclampsia and fetal compromise.

   Dyer RA, Emmanuel A, Adams SC, Lombard CJ, Arcache MJ, Vorster A, Wong CA, Higgins N, Reed AR, James MF, Joolay Y, Schulein S, van Dyk D.

   Int J Obstet Anesth. 2017 Aug 11. pii: S0959-289X(17)30172-3. doi: 10.1016/j.ijoa.2017.08.001. [Epub ahead of print]

   PMID: 28899735 [PubMed - as supplied by publisher]

   Similar articles

10. Core Temperature Monitoring in Obstetric Spinal Anesthesia Using an Ingestible Telemetric Sensor.

    du Toit L, van Dyk D, Hofmeyr R, Lombard CJ, Dyer RA.

    Anesth Analg. 2018 Jan;126(1):190-195. doi: 10.1213/ANE.0000000000002326.

    PMID: 28799968 [PubMed - indexed for MEDLINE]

    Similar articles

11. Support for alcohol policies from drinkers in the City of Tshwane, South Africa: Data from the International Alcohol Control study.

    Parry CDH, Trangenstein P, Lombard C, Jernigan DH, Morojele NK.

    Drug Alcohol Rev. 2017 May 10. doi: 10.1111/dar.12554. [Epub ahead of print]

    PMID: 28493419 [PubMed - as supplied by publisher]

    Similar articles

12. Breastfeeding patterns and its determinants among mothers living with Human Immuno-deficiency Virus -1 in four African countries participating in the ANRS 12174 trial.

    Somé EN, Engebretsen IMS, Nagot N, Meda N, Lombard C, Vallo R, Peries M, Kankasa C, Tumwine JK, Hofmeyr GJ, Singata M, Harper K, Van De Perre P, Tylleskar T; ANRS 12174 Trial Group.

    Int Breastfeed J. 2017 May 2;12:22. doi: 10.1186/s13006-017-0112-2. eCollection 2016.

    PMID: 28469697 [PubMed] **Free PMC Article**

    Similar articles

13. Scanty smears associated with initial loss to follow-up in South African tuberculosis patients.

    Claassens MM, Dunbar R, Yang B, Lombard CJ.

    Int J Tuberc Lung Dis. 2017 Feb 1;21(2):196-201. doi: 10.5588/ijtld.16.0292.

    PMID: 28234084 [PubMed - in process]

    Similar articles

14. Has universal screening with Xpert® MTB/RIF increased the proportion of multidrug-resistant tuberculosis cases diagnosed in a routine operational setting?

    Naidoo P, Dunbar R, Caldwell J, Lombard C, Beyers N.

    PLoS One. 2017 Feb 15;12(2):e0172143. doi: 10.1371/journal.pone.0172143. eCollection 2017.

    PMID: 28199375 [PubMed - indexed for MEDLINE] **Free PMC Article**

    Similar articles

15. Structural Level Differences in the Mother-to-Child HIV Transmission Rate in South Africa: A Multilevel Assessment of Individual-, Health Facility-, and Provincial-Level Predictors of Infant HIV Transmission.

Woldesenbet SA, Jackson DJ, Lombard CJ, Dinh TH, Ramokolo V, Doherty T, Sherman GG, Pillay Y, Goga AE.

J Acquir Immune Defic Syndr. 2017 Apr 15;74(5):523-530. doi: 10.1097/QAI.0000000000001289.

PMID: 28107227 [PubMed - indexed for MEDLINE] **Free PMC Article**

Similar articles

16. Socioeconomic and modifiable predictors of blood pressure control for hypertension in primary care attenders in the Western Cape, South Africa.

Folb N, Bachmann MO, Bateman ED, Steyn K, Levitt NS, Timmerman V, Lombard C, Gaziano TA, Fairall LR.

S Afr Med J. 2016 Dec 1;106(12):1241-1246. doi: 10.7196/SAMJ.2016.v106.i12.12005.

PMID: 27917771 [PubMed]

Similar articles

17. Sex differences in insulin sensitivity and insulin response with increasing age in black South African men and women.

Goedecke JH, George C, Veras K, Peer N, Lombard C, Victor H, Steyn K, Levitt NS.

Diabetes Res Clin Pract. 2016 Dec;122:207-214. doi: 10.1016/j.diabres.2016.11.005. Epub 2016 Nov 17.

PMID: 27889690 [PubMed - indexed for MEDLINE]

Similar articles

18. Educational Outreach with an Integrated Clinical Tool for Nurse-Led Non-communicable Chronic Disease Management in Primary Care in South Africa: A Pragmatic Cluster Randomised Controlled Trial.

Fairall LR, Folb N, Timmerman V, Lombard C, Steyn K, Bachmann MO, Bateman ED, Lund C, Cornick R, Faris G, Gaziano T, Georgeu-Pepper D, Zwarenstein M, Levitt NS.

PLoS Med. 2016 Nov 22;13(11):e1002178. doi: 10.1371/journal.pmed.1002178. eCollection 2016 Nov.

PMID: 27875542 [PubMed - indexed for MEDLINE] **Free PMC Article**

Similar articles

19. School-based interventions for preventing HIV, sexually transmitted infections, and pregnancy in adolescents.

Mason-Jones AJ, Sinclair D, Mathews C, Kagee A, Hillman A, Lombard C.

Cochrane Database Syst Rev. 2016 Nov 8;11:CD006417. Review.

PMID: 27824221 [PubMed - indexed for MEDLINE] **Free PMC Article**

Similar articles

20. Changes in drinking patterns during and after pregnancy among mothers of children with fetal alcohol syndrome: A study in three districts of South Africa.

Urban MF, Olivier L, Louw JG, Lombard C, Viljoen DL, Scorgie F, Chersich MF.

Drug Alcohol Depend. 2016 Nov 1;168:13-21. doi: 10.1016/j.drugalcdep.2016.08.629. Epub 2016 Sep 1.

PMID: 27610936 [PubMed - indexed for MEDLINE]

Similar articles

21. Differential associations of cardiovascular disease risk factors with relative wealth in urban-dwelling South Africans.

Peer N, Lombard C, Steyn K, Levitt N.

J Public Health (Oxf). 2016 Sep;38(3):e232-e239. Epub 2015 Oct 31.

PMID: 26521021 [PubMed - indexed for MEDLINE]

Similar articles

22. A parenting programme to prevent abuse of adolescents in South Africa: study protocol for a randomised controlled trial.

Cluver L, Meinck F, Shenderovich Y, Ward CL, Romero RH, Redfern A, Lombard C, Doubt J, Steinert J, Catanho R, Wittesaele C, De Stone S, Salah N, Mpimpilashe P, Lachman J, Loening H, Gardner F, Blanc D, Nocuza M, Lechowicz M.

Trials. 2016 Jul 19;17(1):328. doi: 10.1186/s13063-016-1452-8.

PMID: 27435171 [PubMed - indexed for MEDLINE] **Free PMC Article**

Similar articles

23. Increased risk of dysglycaemia in South Africans with HIV; especially those on protease inhibitors.

    Levitt NS, Peer N, Steyn K, Lombard C, Maartens G, Lambert EV, Dave JA.

    Diabetes Res Clin Pract. 2016 Sep;119:41-7. doi: 10.1016/j.diabres.2016.03.012. Epub 2016 Apr 23.

    PMID: 27423428 [PubMed - indexed for MEDLINE]

    Similar articles

24. Effects of an exclusive breastfeeding intervention for six months on growth patterns of 4-5 year old children in Uganda: the cluster-randomised PROMISE EBF trial.

    Fadnes LT, Nankabirwa V, Engebretsen IM, Sommerfelt H, Birungi N, Lombard C, Swanevelder S, Van den Broeck J, Tylleskär T, Tumwine JK; PROMISE-EBF Study Group.

    BMC Public Health. 2016 Jul 12;16:555. doi: 10.1186/s12889-016-3234-3.

    PMID: 27405396 [PubMed - indexed for MEDLINE] **Free PMC Article**

    Similar articles

25. Trial size, HIV pre-exposure prophylaxis, and breastfeeding - Authors' reply.

    Lombard C, Cousens S, Tylleskär T, Van de Perre P, Nagot N.

    Lancet. 2016 May 21;387(10033):2091. doi: 10.1016/S0140-6736(16)30540-2. No abstract available.

    PMID: 27301822 [PubMed - indexed for MEDLINE]

    Similar articles

26. Dietary Intake of the Urban Black Population of Cape Town: The Cardiovascular Risk in Black South Africans (CRIBSA) Study.

    Steyn NP, Jaffer N, Nel J, Levitt N, Steyn K, Lombard C, Peer N.

    Nutrients. 2016 May 13;8(5). pii: E285. doi: 10.3390/nu8050285.

    PMID: 27187459 [PubMed - indexed for MEDLINE] **Free PMC Article**

    Similar articles

27. Gender Differences in Homicide of Neonates, Infants, and Children under 5 y in South Africa: Results from the Cross-Sectional 2009 National Child Homicide Study.

    Abrahams N, Mathews S, Martin LJ, Lombard C, Nannan N, Jewkes R.

    PLoS Med. 2016 Apr 26;13(4):e1002003. doi: 10.1371/journal.pmed.1002003. eCollection 2016 Apr.

    PMID: 27115771 [PubMed - indexed for MEDLINE] **Free PMC Article**

    Similar articles

28. Effect on mortality of point-of-care, urine-based lipoarabinomannan testing to guide tuberculosis treatment initiation in HIV-positive hospital inpatients: a pragmatic, parallel-group, multicountry, open-label, randomised controlled trial.

    Peter JG, Zijenah LS, Chanda D, Clowes P, Lesosky M, Gina P, Mehta N, Calligaro G, Lombard CJ, Kadzirange G, Bandason T, Chansa A, Liusha N, Mangu C, Mtafya B, Msila H, Rachow A, Hoelscher M, Mwaba P, Theron G, Dheda K.

    Lancet. 2016 Mar 19;387(10024):1187-97. doi: 10.1016/S0140-6736(15)01092-2. Epub 2016 Mar 10.

    PMID: 26970721 [PubMed - indexed for MEDLINE]

    Similar articles

29. Comparing Tuberculosis Diagnostic Yield in Smear/Culture and Xpert® MTB/RIF-Based Algorithms Using a Non-Randomised Stepped-Wedge Design.

    Naidoo P, Dunbar R, Lombard C, du Toit E, Caldwell J, Detjen A, Squire SB, Enarson DA, Beyers N.

    PLoS One. 2016 Mar 1;11(3):e0150487. doi: 10.1371/journal.pone.0150487. eCollection 2016.

    PMID: 26930400 [PubMed - indexed for MEDLINE] **Free PMC Article**

    Similar articles

30. Cost-Effectiveness of Peer Counselling for the Promotion of Exclusive Breastfeeding in Uganda.

    Chola L, Fadnes LT, Engebretsen IM, Nkonki L, Nankabirwa V, Sommerfelt H, Tumwine JK, Tylleskar T,

Robberstad B; PROMISE-EBF Study Group.

PLoS One. 2015 Nov 30;10(11):e0142718. doi: 10.1371/journal.pone.0142718. eCollection 2015.

PMID: 26619338 [PubMed - indexed for MEDLINE] **Free PMC Article**

Similar articles

31. Extended pre-exposure prophylaxis with lopinavir-ritonavir versus lamivudine to prevent HIV-1 transmission through breastfeeding up to 50 weeks in infants in Africa (ANRS 12174): a randomised controlled trial.

Nagot N, Kankasa C, Tumwine JK, Meda N, Hofmeyr GJ, Vallo R, Mwiya M, Kwagala M, Traore H, Sunday A, Singata M, Siuluta C, Some E, Rutagwera D, Neboua D, Ndeezi G, Jackson D, Maréchal V, Neveu D, Engebretsen IM, Lombard C, Blanche S, Sommerfelt H, Rekacewicz C, Tylleskär T, Van de Perre P; ANRS 12174 Trial Group.

Lancet. 2016 Feb 6;387(10018):566-73. doi: 10.1016/S0140-6736(15)00984-8. Epub 2015 Nov 19.

PMID: 26603917 [PubMed - indexed for MEDLINE]

Similar articles

32. Serum retinol in post-partum mothers and newborns from an impoverished South African community where liver is frequently eaten and vitamin A deficiency is absent.

van Stuijvenberg ME, Schoeman SE, Nel J, Lombard CJ, Dhansay MA.

Matern Child Nutr. 2017 Jan;13(1). doi: 10.1111/mcn.12223. Epub 2015 Nov 12.

PMID: 26564246 [PubMed - indexed for MEDLINE]

Similar articles

33. Multimorbidity, control and treatment of noncommunicable diseases among primary healthcare attenders in the Western Cape, South Africa.

Folb N, Timmerman V, Levitt NS, Steyn K, Bachmann MO, Lund C, Bateman ED, Lombard C, Gaziano TA, Zwarenstein M, Fairall LR.

S Afr Med J. 2015 Sep 21;105(8):642-7.

PMID: 26449692 [PubMed - indexed for MEDLINE] **Free PMC Article**

Similar articles

34. The use of phenylephrine to obtund oxytocin-induced hypotension and tachycardia during caesarean section.

Rumboll CK, Dyer RA, Lombard CJ.

Int J Obstet Anesth. 2015 Nov;24(4):297-302. doi: 10.1016/j.ijoa.2015.08.003. Epub 2015 Aug 11.

PMID: 26421702 [PubMed - indexed for MEDLINE]

Similar articles

35. Did HealthKick, a randomised controlled trial primary school nutrition intervention improve dietary quality of children in low-income settings in South Africa?

Steyn NP, de Villiers A, Gwebushe N, Draper CE, Hill J, de Waal M, Dalais L, Abrahams Z, Lombard C, Lambert EV.

BMC Public Health. 2015 Sep 23;15:948. doi: 10.1186/s12889-015-2282-4.

PMID: 26400414 [PubMed - indexed for MEDLINE] **Free PMC Article**

Similar articles

36. Implementation of the HealthKick intervention in primary schools in low-income settings in the Western Cape Province, South Africa: a process evaluation.

de Villiers A, Steyn NP, Draper CE, Hill J, Dalais L, Fourie J, Lombard C, Barkhuizen G, Lambert EV.

BMC Public Health. 2015 Aug 22;15:818. doi: 10.1186/s12889-015-2157-8.

PMID: 26297447 [PubMed - indexed for MEDLINE] **Free PMC Article**

Similar articles

37. Tenofovir Gel for the Prevention of Herpes Simplex Virus Type 2 Infection.

Abdool Karim SS, Abdool Karim Q, Kharsany AB, Baxter C, Grobler AC, Werner L, Kashuba A, Mansoor LE, Samsunder N, Mindel A, Gengiah TN; CAPRISA 004 Trial Group.

N Engl J Med. 2015 Aug 6;373(6):530-9. doi: 10.1056/NEJMoa1410649.

PMID: 26244306 [PubMed - indexed for MEDLINE] **Free PMC Article**

Similar articles

38. Missed Opportunities along the Prevention of Mother-to-Child Transmission Services Cascade in South Africa: Uptake, Determinants, and Attributable Risk (the SAPMTCTE).

    Woldesenbet S, Jackson D, Lombard C, Dinh TH, Puren A, Sherman G, Ramokolo V, Doherty T, Mogashoa M, Bhardwaj S, Chopra M, Shaffer N, Pillay Y, Goga A; South African PMTCT Evaluation (SAPMCTE) Team.

    PLoS One. 2015 Jul 6;10(7):e0132425. doi: 10.1371/journal.pone.0132425. eCollection 2015.

    PMID: 26147598 [PubMed - indexed for MEDLINE] **Free PMC Article**

    Similar articles

39. The complex relationship between human immunodeficiency virus infection and death in adults being treated for tuberculosis in Cape Town, South Africa.

    Osman M, Seddon JA, Dunbar R, Draper HR, Lombard C, Beyers N.

    BMC Public Health. 2015 Jun 18;15:556. doi: 10.1186/s12889-015-1914-z.

    PMID: 26082037 [PubMed - indexed for MEDLINE] **Free PMC Article**

    Similar articles

40. Chest radiographic abnormalities in HIV-infected African children: a longitudinal study.

    Pitcher RD, Lombard CJ, Cotton MF, Beningfield SJ, Workman L, Zar HJ.

    Thorax. 2015 Sep;70(9):840-6. doi: 10.1136/thoraxjnl-2014-206105. Epub 2015 Jun 9.

    PMID: 26060256 [PubMed - indexed for MEDLINE]

    Similar articles

41. Correction: Impact of Maternal HIV Seroconversion during Pregnancy on Early Mother to Child Transmission of HIV (MTCT) Measured at 4-8 Weeks Postpartum in South Africa 2011-2012: A National Population-Based Evaluation.

    Dinh TH, Delaney KP, Goga A, Jackson D, Lombard C, Woldesenbet S, Mogashoa M, Pillay Y, Shaffer N.

    PLoS One. 2015 Jun 4;10(6):e0130321. doi: 10.1371/journal.pone.0130321. eCollection 2015. No abstract available.

    PMID: 26043225 [PubMed] **Free PMC Article**

    Similar articles

42. Impact of Maternal HIV Seroconversion during Pregnancy on Early Mother to Child Transmission of HIV (MTCT) Measured at 4-8 Weeks Postpartum in South Africa 2011-2012: A National Population-Based Evaluation.

    Dinh TH, Delaney KP, Goga A, Jackson D, Lombard C, Woldesenbet S, Mogashoa M, Pillay Y, Shaffer N.

    PLoS One. 2015 May 5;10(5):e0125525. doi: 10.1371/journal.pone.0125525. eCollection 2015. Erratum in: PLoS One. 2015;10(6):e0130321.

    PMID: 25942423 [PubMed - indexed for MEDLINE] **Free PMC Article**

    Similar articles

43. Prevalence of fetal alcohol syndrome in a South African city with a predominantly Black African population.

    Urban MF, Olivier L, Viljoen D, Lombard C, Louw JG, Drotsky LM, Temmerman M, Chersich MF.

    Alcohol Clin Exp Res. 2015 Jun;39(6):1016-26. doi: 10.1111/acer.12726. Epub 2015 May 2.

    PMID: 25941030 [PubMed - indexed for MEDLINE]

    Similar articles

44. Low intake of calcium and vitamin D, but not zinc, iron or vitamin A, is associated with stunting in 2- to 5-year-old children.

    van Stuijvenberg ME, Nel J, Schoeman SE, Lombard CJ, du Plessis LM, Dhansay MA.

    Nutrition. 2015 Jun;31(6):841-6. doi: 10.1016/j.nut.2014.12.011. Epub 2014 Dec 31.

    PMID: 25933491 [PubMed - indexed for MEDLINE]

    Similar articles

45. Effects of early feeding on growth velocity and overweight/obesity in a cohort of HIV unexposed South African infants and children.

Ramokolo V, Lombard C, Chhagan M, Engebretsen IM, Doherty T, Goga AE, Fadnes LT, Zembe W, Jackson DJ, Van den Broeck J.

Int Breastfeed J. 2015 Apr 2;10:14. doi: 10.1186/s13006-015-0041-x. eCollection 2015. Erratum in: Int Breastfeed J. 2017 Nov 13;12 :47.

PMID: 25873986 [PubMed] **Free PMC Article**

Similar articles

46. Predictors of poor adherence among people on antiretroviral treatment in Cape Town, South Africa: a case-control study.

    Dewing S, Mathews C, Lurie M, Kagee A, Padayachee T, Lombard C.

    AIDS Care. 2015;27(3):342-9. doi: 10.1080/09540121.2014.994471. Epub 2015 Jan 3.

    PMID: 25559444 [PubMed - indexed for MEDLINE] **Free PMC Article**

47. First population-level effectiveness evaluation of a national programme to prevent HIV transmission from mother to child, South Africa.

    Goga AE, Dinh TH, Jackson DJ, Lombard C, Delaney KP, Puren A, Sherman G, Woldesenbet S, Ramokolo V, Crowley S, Doherty T, Chopra M, Shaffer N, Pillay Y; South Africa PMTCT Evaluation Team.

    J Epidemiol Community Health. 2015 Mar;69(3):240-8. doi: 10.1136/jech-2014-204535. Epub 2014 Nov 4.

    PMID: 25371480 [PubMed - indexed for MEDLINE] **Free PMC Article**

8. Date of last GCP training (as a participant or presenter)

9. Any additional relevant information supporting abilities to participate in conducting this research. [Briefly]

I have been involved with the two previous national surveys on this topic conducted by the SAMRC

NAME IN FULL: Carl Jacobus Lombard

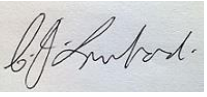

Signature: **Date:** _5 Februrary 2018_

Appendix L: Prof. Mathews

CV FORMAT FOR HEALTH PROFESSIONALS

Trial title: A national study of male, female and child homicide in South Africa

Protocol no: /

Designation:

1. Personal details

Name	Shanaaz Mathews	Tel no.	021 5311965
Address	22 Alices Ride	Work tel no.	021 6501473
	Pinelands	Fax no.	021 6501460
	Cape Town	Cell phone no	0832461676
		Email	shanaaz.mathews@uct.ac.za

2. Academic and professional qualifications

Degree	Field of study	University	Year
PhD	Public Health	University of the Witwatersrand	2010
Masters	Public Health	University of Cape Town	2005
B.Soc.Sc. Hons	Social Work	University of Cape Town	1986
B.Soc.Sc.		University of Cape Town	1995

3. Health Professions Council of South Africa (HPCSA) registration number if applicable (or other health professions body registration particulars if applicable – e.g. Nursing Council)

N/A

4. Current personal medical malpractice insurance details [medical and dental practitioners] (please put MRC, not N/A, if you do not have other insurance)

N/A

5. Relevant related work experience (brief) and current position

Period	Position	Employer
October 2012 – to date	Director / Professor	Children's Institute, Faculty of Health Sciences, University of Cape Town
Jan 2002 – Sept 2012	Specialist Scientist	Gender and Health Research Unit, SA Medical Research Council
Jan 1999 – Dec 2001	Programme Manager	Gender Advocacy Programme
April 1997 – Dec 1998	Programme Manager	Western Cape Community Partnerships Project University of the Western Cape
Sept 1987 – February 1997	Clinical Social Worker	Red Cross Children's Hospital
January 1987 – August 1987	Social Worker	Dept of Health and Welfare

6. Participation in clinical trials research in the last three years (title, protocol number, designation) [If multiple trials, only list those with relevance to this application, or in the last year.]

Nil

7. Peer-reviewed publications in the past 3 years

- Osano B, Were F & **Mathews S.** 2017. Mortality among 5-17-year old children in Kenya. Pan Afr Med J: 27: 121 doi:10.11604/pamj.2017.27.121.10727
- Abrahams N, **Mathews S**, Martin LJ, Lombard C & Jewkes R. (2017). Sexual homicides in South Africa: a national study of adult females and children. PLoS ONE 12(10): e0186432. https://doi.org/10.1371/journal.pone.0186432
- Dawson M, **Mathews S**, Abrahams N & Campbell J, 2017. Death reviews in the context of domestic homicide in low- to middle-income countries: South Africa as a case study. In (ed) Dawson M. *Domestic Homicides and Death Reviews: An International Perspective.* Palgrave Macmillan Hampshire UK.
- **Mathews S** & Martin L., 2016 Developing an understanding of fatal child abuse and neglect: Results from the South African child death review pilot. 106(12):1160-1163. DOI:10.7196.
- **Mathews S**, Martin L, Coetzee D, Scott, C & Brijmohun Y. 2016. Child deaths in South Africa: Lessons from the child death review pilot. SAMJ, 106(9), 851-852.
- **Mathews S**, Martin L, Coetzee D, Scott, C, Naidoo T, Brijmohun Y & Quarrie K. 2016. The South African child death review pilot: a multi-agency approach to strengthen healthcare and protection for children. SAMJ. 106(9), 895-899.
- **Mathews S**, Hendricks N & Abrahams N. 2016. A Psychosocial Understanding of child sexual abuse disclosure among a group of female children in Cape Town, South Africa. Child Sexual Abuse. 25:6, 636-654, DOI: 10.1080/10538712.2016.1199078
- Abrahams N, **Mathews S**, Martin LJ, Lombard C, Nannan N & Jewkes R. 2016. Gender differences in homicide of neonates, infants and under-five year olds in South Africa: Results from the 2009 national child homicide study. Plosmed. 13(4): 1-15. e1002003. doi:10.1371/journal. pmed.1002003.
- Matzopoulos R, Prinsloo M, Bradshaw D, Pillay-van Wyk V, Gwebushe N, **Mathews S**, Martin L, Laubscher R, Lombard C, Abrahams N. (2015) Analysis of South African injury mortality based on the first nationally representative sample: a retrospective study of post mortem investigations, *Bulletin of the WHO*, 2015 Mar 13. DOI: 10.2471/BLT.14.145771

8. Date of last GCP training (as a participant or presenter)

July 2015 (participant)

9. Any additional relevant information supporting abilities to participate in conducting this research. [Briefly]

I was part of the team that implemented the first female homicide study in 2002 – 2004 and was responsible for the field work. I was also part of the team that conceptualised the second female homicide and child homicide study. I was responsible for the implementation of the field work of the study; initial data management analysis. I have also been part of the team that led the National Injury Mortality Survey.

NAME IN FULL

Signature: 19/12/2017

Date:

Appendix M: Dr. Matzopoulos

CV FORMAT FOR HEALTH PROFESSIONALS

Trial title: **A national study of injury-related mortality, with a focus on homicide in South Africa**

Protocol no: /

Designation: Deputy Director. Burden of Disease Research Unit, Medical Research Council.

1. Personal details

**Name**	Richard Matzopoulos	**Tel no.**	/
**Address**	University of Cape Town<br>School of Public Health and Family Medicine<br>Anzio Rd<br>Observatory 7925<br>South Africa	**Work tel no.**	021 938 0305/0327
		**Fax no.**	021 406 6163
		**Cell phone no**	/
		**Email**	Richard.Matzopoulos@mrc.ac.za

2. Academic and professional qualifications

Degree	Field of study	University	Year
PhD	Public Health	University of Cape Town	2007 - 2012
MPhil	Epidemiology	University of Cape Town	1997 - 2001
BBusSci	Operational Research and Statistics	University of Cape Town	1990 - 1994

3. Health Professions Council of South Africa (HPCSA) registration number if applicable (or other health professions body registration particulars if applicable – e.g. Nursing Council) /

4. Current personal medical malpractice insurance details [medical and dental practitioners] (please put MRC, not N/A, if you do not have other insurance) MRC

5. Relevant related work experience (brief) and current position

Period	Position	Employer
September 2017 – pre	Deputy Director:<br>Burden of Disease<br>South African Medical Research Cou	SAMRC
April 2015 – present	Epidemiologist (Chief Specialist Scien<br>Burden of Disease Research Unit, SAMRC	SAMRC
February 2013 – March 2015	Epidemiologist (Senior Specialist Scientist). Burden of Disease Researc Unit, Medical Research Council.	SAMRC
September 2009 – Jar 2013	Epidemiologist (Specialist Scientist). Burden of Disease Researc Unit, Medical Research Council.	SAMRC

6. Participation in clinical trials research in the last three years (title, protocol number, designation) [If multiple trials, only list those with relevance to this application, or in the last year.] /

7. **Peer-reviewed publications in the past 3 years**

1. Jabar A, Oni T, Engel M, Cvetkovic N, **Matzopoulos R**. *(in press).* Rationale and design of the Violence, injury and trauma observatory (VITO): The Cape Town VITO pilot studies protocol. *BMJ Open*

2. ***Matzopoulos R**, Simonetti J, Prinsloo M, Neethling I, Groenewald P, Dempers J, Martin LJ, Rowhani-Rahbar A, Myers J, Thompson ML. (in press).* A retrospective time trend study of firearm and non-firearm homicide in Cape Town from 1994 to 2013. *South African Medical Journal*.

3. Groenewald P, Neethling I, Evans J, Azevedo V, Naledi T, **Matzopoulos R**, Daniels J, Bradshaw D. (in press). Mortality trends in Cape Town Metro between 2001 and 2013: reducing inequities in health. *South African Medical Journal*.

4. GBD 2016 Risk Factors Collaborators. Global, regional, and national comparative risk assessment of 84 behavioural, environmental and occupational, and metabolic risks or clusters of risks, 1990–2016: a systematic analysis for the Global Burden of Disease Study 2016. Lancet [Internet]. 2017;390(10100):1345–422. Available from: http://linkinghub.elsevier.com/retrieve/pii/S0140673617323668
5. Prinsloo M, Bradshaw D, Joubert J, **Matzopoulos R**, Groenewald P. 2017. South Africa's vital statistics are currently not suitable for monitoring progress towards injury and violence Sustainable Development Goals. *South African Medical Journal* 107(6):470. http://www.samj.org.za/index.php/samj/article/view/11922

6. Jabar, A. & **Matzopoulos, R**., 2017. Violence and injury observatories. *South African Crime Quarterly*, (59), pp.47–57.

7. Pillay-van Wyk V, Msemburi W, Laubscher R, Dorrington RE, Groenewald P, Glass T, Nojilana B, Joubert JD, **Matzopoulos R**, Prinsloo M, Nannan N, Gwebushe N, Vos T, Somdyala N, Sithole N, Neethling I, Nicol E, Rossouw A, Bradshaw D. 2016. Mortality trends and differentials in South Africa, 1997-2012. *Lancet Global Health*. 4(9):e642-e653.

8. Oni T, Smit W, **Matzopoulos R**, Hunter Adams J, Rother A, Pentecost M, Albertyn Z, Behroozi F, Alaba O, Kaba M, Van der Westhuizen C, Shung King M, Parnell S, Levitt NS, Lambert EV. 2016. Urban health research in Africa: themes and priority research questions. *Journal of Urban Health* 93(4):722-30.

9. Jabar A, Lawal A, Mehtar Z, **Matzopoulos R**. 2016. Substance Abuse Programs that Reduce Violence in a Youth Population: A Systematic Review. *Journal of Alcohol and Drug Education*. 60(2):8-15.

10. GBD 2015 Risk Factors Collaborators. 2016. Global, regional, and national comparative risk assessment of 79 behavioural, environmental and occupational, and metabolic risks or clusters of risks, 1990-2015: a systematic analysis for the global burden of disease study 2015. *Lancet*. 388:1658-1724.

11. GBD 2015 Mortality and Causes of Death Collaborators. 2016. Global, regional, and national life expectancy, all-cause mortality, and cause-specific mortality for 249 causes of death, 1980-2015: a systematic analysis for the global burden of disease study 2015. *Lancet*. 388:1459-1544.

12. GBD 2015 DALYs and HALE Collaborators. 2016. Global, regional, and national disability-adjusted life-years (DALYs) for 315 diseases and injuries and healthy life expectancy (HALE), 1990–2015: a systematic analysis for the global burden of disease study 2015. *Lancet*. 388:1603-1658.

13. GBD 2015 SDG Collaborators. 2016. Measuring the health-related sustainable development goals in 188 countries: a baseline analysis from the global burden of disease study 2015. Lancet: 1813–1850.

14. **Matzopoulos R**. 2016. ICECI: Injury surveillance in South Africa. *Injury Prevention.* 22(2): A17.

15. Haagsma JA, Graetz N, Bolliger I, Naghavi M, Higashi H, Mullany EC, Abera SF, Abraham JP, et al. 2016. The global burden of injury: incidence, mortality, disability-adjusted life years, and time trends from the Global Burden of Disease Study 2013. *Injury Prevention.* 22:3-18.

16. Cassidy A, Bowman B, McGrath C, **Matzopoulos R**. 2016 (Jul). Brief report on a systematic review of youth violence prevention through media campaigns: Does the limited yield of strong

evidence imply methodological challenges or absence of effect? *Journal of Adolescence* 52: 22-26.

17. **Matzopoulos R**. 2016 (Jun). Gun control saves lives. *South African Medical Journal*. 106(6):544.
18. **Matzopoulos R**, Groenewald P, Abrahams N, Bradshaw D. 2016 (Jun). Where have all the gun deaths gone? *South African Medical Journal* 106(6):589-591.
19. **Matzopoulos R**, Bowman B. 2016 (May). SDGs put violence prevention on the map. *Journal of Public Health Policy* 37: 260-262. doi: 10.1057/jphp.2016.13
20. Prinsloo M, **Matzopoulos R**, Laubscher R, Myers J, Bradshaw D. 2016 (Feb). Validating the decline in Western Cape homicide rates: findings from the 2009 Injury Mortality Survey. *South African Medical Journal* 106(2):193-195.

8. Date of last GCP training (as a participant or presenter)

/

9. Any additional relevant information supporting abilities to participate in conducting this research. [Briefly]

NAME IN FULL

Signature: ____________________ **Date:** ________________

Appendix N: Prof. Labuschagne

CV FORMAT FOR HEALTH PROFESSIONALS

Trial title: A national study of male, female and child homicide in South Africa

Protocol no: /

Designation:

1. Personal details

Name	*Gerard Nicholas Labuschagne*	Tel no.	*0824140527*
Address	*542 Alaska Road*	Work tel no.	
	Faerie Glen	Fax no.	
	Pretoria	Cell phone no	*0824140527*
	0081	Email	*doc@threatsa.co.za*

2. Academic and professional qualifications

Degree	Field of study	University	Year
BA	*Psychology*	*Pretoria*	*1993*
BA(Hons)	*Psychology*	*Pretoria*	*1995*
MA	*Clin Psychol*	*Pretoria*	*1997*
MA	*Criminology*	*Pretoria*	*2007*
PhD	*Psychology*	*Pretoria*	*2001*
LLB	*Law*	*UNISA*	*2015*

3. Health Professions Council of South Africa (HPCSA) registration number if applicable (or other health professions body registration particulars if applicable – e.g. Nursing Council)
Professional Board for Psychology (HPCSA) PS0059048

4. Current personal medical malpractice insurance details [medical and dental practitioners] (please put MRC, not N/A, if you do not have other insurance)
MPS

5. Relevant related work experience (brief) and current position

Period	Position	Employer
1998-2001	*Clinical Psychologist*	*Gauteng Dept Health/ UP*
2001-2016	*Brigadier*	*SAPS*
2013- current	*Hon Ass Professor*	*Forensic Medicine: WITS*
2013-2016	*Extraordinary Prof*	*Dept Police Practice: UNISA*
2008-2011	*Extraordinary Prof*	*Dept Criminology: UNISA*
2008-2016	*Adjunct Faculty Member*	*California School of Forensic Studies*
2016-current	*Director*	*L&S Threat Management Pty Ltd*

6. Participation in clinical trials research in the last three years (title, protocol number, designation) [If multiple trials, only list those with relevance to this application, or in the last year.]
No clinical trials research.

7. Peer-reviewed publications in the past 3 years
Labuschagne, G.N. (2015). Criminal Investigative Analysis: An Applied Perspective. In Zinn, R.

Dintwe, S. (Eds.), Forensic Investigation: Legislative Principles and Investigative Practice. Cape Town: Juta & Co.

Van der Watt, M., Benson, B., & Labuschagne, G. (2015). From 'stranger' to 'serial': (Re)emphasising the value of docket analysis as a linkage tool in serial rape identification. Acta Criminologica: Southern African Journal of Criminology, 28(2), 62-77.

Labuschagne, G.N. (2017). Stewart "Boetie Boer" Wilken: Serial murder, necrophilia, and cannibalism: A South African case study. In L. Mellor, A. Aggrawal & E. Hickey (Eds.), Understanding Necrophilia: A Global Multidisciplinary Approach (pp.411-417). USA: Cognella Academic Publishing.

8. Date of last GCP training (as a participant or presenter)

9. Any additional relevant information supporting abilities to participate in conducting this research.

14,5 years experience in the SAPS as the head of the Investigative Psychology Section. This involved assisting in the investigation of high profile murder cases throughout the Republic. Trained as a homicide investigator by the Los Angeles Sheriffs Department. Participated in the MRC's national rape study released in 2017.

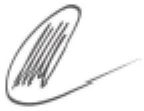

____

Gerard Nicholas Labuschagne

Date: ___03/01/2017___

Appendix O: Miss Dekel

CV FORMAT FOR HEALTH PROFESSIONALS

Trial title: **A national study of injury-related mortality, with a focus on homicide in South Africa**

Protocol no: /

Designation: Researcher

1. Personal details

Name	**Bianca Dekel**	Tel no.	**/**
Address	**Francie van Zijl Drive**	Work tel no.	**021 9380838**
	**Tygerberg**	Fax no.	
	**Cape Town**	Cell phone no	**0769403941**
		Email	**Bianca.dekel@mrc.ac.za**

2. Academic and professional qualifications

Degree	Field of study	University	Year
**BA Degree**	**Psychology**	**UNISA**	**2011**
**BA Honours Degree**	**Psychology**	**UWC**	**2012**
**BA Master's Degree**	**Research Psychology**	**UWC**	**2013**
**PhD: Psychology**	**Psychology**	**UWC**	**2015-2018**

3. Health Professions Council of South Africa (HPCSA) registration number if applicable (or other health professions body registration particulars if applicable – e.g. Nursing Council)
PS 0128880

4. Current personal medical malpractice insurance details [medical and dental practitioners] (please put MRC, not N/A, if you do not have other insurance) **MRC**

5. Relevant related work experience (brief) and current position

Period	Position	Employer
**2012-2013**	**Workshop Facilitator**	**HSRC**
**2013**	**Research Assistant**	**UWC**
**2014**	**Master's Research Intern**	**VIPRU: SAMRC**
**2015-Current**	**PhD Candidate**	**G&H: SAMRC**

6. Participation in clinical trials research in the last three years (title, protocol number, designation) [If multiple trials, only list those with relevance to this application, or in the last year.] **N/A**

7. Peer-reviewed publications in the past 3 years

- Hendricks, G., Savahl, S., Mathews, K., Raats, C., Jaffer, L., Matzdorff, A., **Dekel, B.,** Larke, C., Magodyo, T., van Gesselleen, M., & Pedro, A. (2015). Influences on life aspirations among adolescents in a low-income community in Cape Town, South Africa. *Journal of Psychology in Africa, 25*(4), 320-326.
- **Dekel, B.,** & Andipatin, M. (2016). Abused Women's Understandings of Intimate Partner Violence and the Link to Intimate Femicide. Forum Qualitative Sozialforschung /Forum: Qualitative Social Research, 17(1), Art. 9, http://nbn-resolving.de/urn:nbn:de:0114-fqs160196.
- Stöckl, H., **Dekel, B**., Morris-Gehring, A., Watts, C., & Abrahams, N. (2017). Child homicide perpetrators worldwide - a systematic review. *BMJ Paediatrics Open, 1*(1), e000112.
- **Dekel, B**., & Van Niekerk, A. (In Press). Women's Recovery, Negotiation of Appearance, and Social Reintegration Following a Burn Injury.
- **Dekel, B.,** Stokl, H., Andipatin, M., & Abrahams, N. (Under review). Mapping the scientific evidence of infant homicide: A systematic review.

- **<u>Dekel, B.,</u>** Abrahams, N., & Andipatin, M. (Under review). Exploring adverse parent-child relationships from the perspective of convicted child murderers: A South African
- **<u>Dekel, B.,</u>** Abrahams, N., & Andipatin, M. (Under review). Towards a feminist understanding of the intersection between violence against women and violence against children from the perspective of parents and caregivers convicted of child homicide in South Africa
- **<u>Dekel, B.,</u>** Abrahams, N., & Andipatin, M. (Under review). The role of poly-victimization within multiple domains: Using an ecological framework to understand convicted parental child murderers pathways to violence in South Africa

8. Date of last GCP training (as a participant or presenter) **30 January 2017**

9. Any additional relevant information supporting abilities to participate in conducting this research. [Briefly]

NAME IN FULL

Signature: **Date:**

Appendix P: Miss Prinsloo

CV FORMAT FOR HEALTH PROFESSIONALS

Trial title: A national study of injury-related mortality, with a focus on homicide in South Africa

Protocol no: /

Designation:

1. Personal details

Name	Megan Prinsloo	Tel no.	
Address	SA Medical Research Council (SAMRC)	Work tel no.	021 938 0952
	P.O. Box 19070	Fax no.	086 679 9611
	Tygerberg, 7505	Cell phone no	
	Cape Town, 8000	Email	megan.prinsloo@mrc.ac.za

2. Academic and professional qualifications

Degree	Field of study	University	Year
PhD (current studies)	Public Health	University of Cape Town	
Masters	Public Health	University of the Western Cape	2004
Postgraduate Diploma	Public Health	University of the Western Cape	2002
Postgraduate Certificate	Public Health	University of the Western Cape	2001
National Diploma	Bio-Medical Technology	Peninsula Technikon	1999

3. Health Professions Council of South Africa (HPCSA) registration number if applicable (or other health professions body registration particulars if applicable – e.g. Nursing Council)

4. Current personal medical malpractice insurance details [medical and dental practitioners] (please put MRC, not N/A, if you do not have other insurance)

5. Relevant related work experience (brief) and current position

Period	Position	Employer
2011-current	Senior Scientist (Entry-Competent)	Burden of Disease Research Unit, SAMRC
2008-2011	Scientist	Burden of Disease Research Unit, SAMRC
2005-2008	Scientist	MRC-UNISA Crime, Violence & Injury Lead Programme
2003-2005	Junior Scientist	MRC-UNISA Crime, Violence & Injury Lead Programme
2001-2003	Research Technologist	MRC-UNISA Crime, Violence & Injury Lead Programme
2000-2001	Research Assistant	Technology & Business Development Group, SAMRC
Jan-Dec 1999	Clinical Pathology Intern	Red Cross Children's Hospital

6. Participation in clinical trials research in the last three years (title, protocol number, designation) [If multiple trials, only list those with relevance to this application, or in the last year.]

Appendix Q: Miss. Rapiya

CV FORMAT FOR HEALTH PROFESSIONALS

Trial title: Homicides in South Africa **Protocol no**:

Designation: Research Technologist

1. Personal details

Name	**Bongwekazi Rapiya**	Tel no.	
Address	**Francie Van Zijl Drive**	Work tel no.	**021 9380822**
	**Parow Valley**	Fax no.	
	**7500**	Cell phone no	**0835505655**
		Email	**brapiya@mrc.ac.za**

2. Academic and professional qualifications

Degree	Field of study	University	Year
**BA HONORS**	**Gender Studies**	**University of South Africa**	**2016**

3. Health Professions Council of South Africa (HPCSA) registration number if applicable (or other health professions body registration particulars if applicable – e.g. Nursing Council)
MP:

4. Current personal medical malpractice insurance details [medical and dental practitioners] (please put MRC, not N/A, if you do not have other insurance)
SAMRC

5. Relevant related work experience (brief) and current position

Period	Position	Employer
**31 May 2012- to date**	**Fieldwork Coordinator**	**GHRU SAMRC**
**01 June 2007- 31 May 2012**	**Fieldwork Coordinator**	**GHRU SAMRC**
**01 June 2006 – 31 March 2007**	**Fieldwork Coordinator**	**Statistics SA**

6. Participation in clinical trials research in the last three years (title, protocol number, designation) [If multiple trials, only list those with relevance to this application, or in the last year.]
No

7. Peer-reviewed publications in the past 3 years

No

8. Date of last GCP training (as a participant or presenter)

2016 As a participant

9. Any additional relevant information supporting abilities to participate in conducting this research. [Briefly]

I have an array of fieldwork experiences, including the 2009 Femicide Study. Having worked in different projects has afforded me the skills below:

- Coordinate and supervise fieldwork, field team and aspects of studies with little or no supervision
- Proactivity and work independently as well as in a team
- Have good persuading skills
- People management and good interpersonal, communication and writing skills
- Understand and adhere to the scientific protocols, policies and procedures
- Limited analysis, interpretation and reporting of research data skills
- Basic understanding of scientific methods
- Data and knowledge Management Methodologies
- Organizational and Presentation skills

NAME IN FULL _Bongwekazi Rapiya_________________ **Signature:**

Date:__13 December 2017___________________

Appendix R: Checklist

ETHICS COMMITTEE CHECKLIST FOR QUANTITATIVE RESEARCH

The **SAMRC Ethics Committee** wishes to process applications for clearance as speedily as possible. To help us do this applicants need to provide a clear and comprehensive protocol for assessment.

Please note that all applications will be checked for completeness by the administration before submission to the Committee. All incomplete proposals will be returned to the applicant for updating which could result in unfortunate delays in the review process.

Below is a checklist to help achieve this.
Please complete and attach the checklist to your submission

		Yes	No
1	**Is the application labelled?**<br>Give project title, unit.	Yes	
2	**Are details of the investigators provided?**<br>Name, title, full mailing address, telephone and fax numbers, e-mail address of principal investigator and co-investigators from each collaborating organisation.	Yes	
3	**Have key words been given?**<br>Up to 6 scientific descriptors (key words) for the project.	Yes	
4	**Is the following declaration provided, dated and signed?**<br>I, - ***name of principal investigator***- have read the Department of Health: *Ethics in health research: principles, processes and structures, second edition*, 2015, the *Guidelines for Good Practice in the Conduct of Clinical Trials with Human Participants in South Africa,* Second Edition, 2006, Department of Health, Pretoria, South Africa (where applicable), and the Declaration of Helsinki (2013) and have prepared this proposal with due cognisance of its content. Furthermore I will adhere to the principles expressed when conducting this proposed research project.	Yes	

Did you have any difficulty with any specific provision in the guidelines concerning your proposal? If so, please provide details - this will be very helpful to the SAMRC Ethics Committee.

____

____

____

Please give a statement of the research problem.

To establish the incidence of homicide in South Africa with a focus on male, female, intimate femicide, and child homicide for the year 2017, and to determine the injury burden. ____

5 **Has the application been approved through the EC's scientific review process?** Yes

____

6 **Has the application been checked for content, grammar and spelling?** Yes

If yes, by whom?

_Prof Abrahams, Miss Bianca Dekel and the scientific reviewers

____

7 **Are the ethics issues identified, and is it stated how they will be addressed?** Yes

____

8 **Are the copies of the following attached?** [N/A = not applicable] — **Yes** **N/A**

- An Executive Summary, stating the AIM, METHODS, OUTCOME and INTENDED FEEDBACK of the study. Yes
- Participant information sheet Yes
- Informed consent form Yes

Are the technical terms in the above forms explained in lay terms? Yes

Are the contact details of the SAMRC Ethics Committee given with a statement that participants can contact the Chairperson when they have queries or problems? (Prof K Moodley, tel. (021) 938 0687; e-mail: adri.labuschagne@mrc.ac.za) Yes

- Translations into languages relevant to the study area. If yes, which language(s)? N/A

____

If consent will be verbal or informed consent is not necessary, please explain why not N/A

____

9 **Is any questionnaire to be used provided?** Yes

10 **Is confidentiality clarified?** Yes

11 **Has consent from minors been explained?** N/A

If participants are under age (less than 18 years) from whom will consent be obtained, e.g. parent, guardian, etc.?

____

If minors will be included, is the application form for non-therapeutic research with minors included?

12 **Has blood sampling been clarified?** N/A

Venous or arterial, the amount and frequency and by whom?

13 **Has any drugs administration been specified?** N/A

If yes, have the drug, dose, frequency, and who will administer it been clarified?

14 **Have drug side-effects been specified?** N/A

15 **Have the following protocol details been provided?**

Table of Contents

Number the pages in the proposal and include page numbers in the contents. Yes

Methodology details: are these included in the protocol?

1 Overall aim and specific objectives. Yes

2 *Background and rationale:* This must be substantial and include references to or details of similar studies, and allow for thorough technical peer review by experts in your field. Yes

3 *Technical work plan:* Describe in considerable detail your overall experimental design, methods and research protocols. Discuss research alternatives if your original assumptions/hypotheses prove incorrect. Yes

4 *Statistical planning:* Has there been consultation with a statistician? If NO, please provide reasons. If YES, please give details including randomisation, sample size and proposed methods of analysis. Yes

5 *Time chart:* Critical path analysis identifying when each activity is to take place. Identify points at which timing is critical (e.g. a season when a particular field study would need to be done). Yes

6 References cited. Yes

7 Description of methods applied. Yes

8 Has this study been approved by the research group you operate in, or by any other peer group, for scientific validity? Yes

If yes, name the group: Gender & Heath approves this study.

Management details: are these included in the protocol?

1 Management approach: Discuss the overall management of the project. Where is managerial responsibility? Consider specific functions such as reporting, financial management, procurement of equipment and research supplies, and management of field activities. Yes

2 Staff and scientific collaboration: Who will do what, when and where? (one page). Yes

3 Facilities: Describe the facilities and resources available for the proposed research. Yes

Budget details: are these included in the protocol?

1 *Budget:* full detailed budget for each year. The following headings can act as a guide: Salaries, equipment, its repair and maintenance, materials and supplies, training, consultation, travel, other, indirect costs/overheads. Yes

2 *Budget justification:* Explain how the individual items of the budget were calculated. Justify major or unusual expenses. Yes

3 Budget summary. Yes

4 Has your research group reviewed and accepted the budget? Yes

5 Do you believe the budget is fully sufficient to conduct the study ethically and scientifically? Yes

6 Has the name of the sponsor of the study (if applicable) been indicated on the participant information sheet? N/A

Details of researchers

CVs (Health Professionals) (non-Health Professionals) and publication lists of all senior personnel involved in the project. Yes

NOTE: Only provide qualifications and scientific experience, e.g. publications, projects, presentations. A one-page biosketch with the ten most important references will suffice.

Other details: are these included in the protocol?

1 *Ethical considerations:* This must address all relevant ethical issues including: details of possible negative consequences to the study animals/participants, information to be given to participants, reporting back procedures to the community/authorities and an example of the consent form to be used. Yes

2 *Additional review bodies:* does this protocol need to be reviewed by another institution N/A

or Ethics Committee? If so, has it been submitted and what was the outcome? Please provide copies of relevant documentation.

3 *Similar studies:* Please list titles of any similar studies previously approved.

National Femicide Study in 1999

National femicide and child homicide study in 2009

4 *Research translation:* Summary details of the implementation of research results and outputs, e.g. policy briefs, new research techniques, diagnostic tools, therapies; health policy development, etc. — Yes

5 Please declare which of the following interests you may have in the study, such as:

• resources paid directly to you or your research account;		No
• potential financial benefits from the outcome of the study;		No
• direct financial interest in the company;		No
• any others;		No
• any gains to your family;		No
• travel sponsorship.		No

6 Name the possible (both positive and negative) short- and long-term consequences of the study. — Yes

7 Does the study have insurance for research-related injury? Are participants informed accordingly in the information sheet? — No

8 If applicable, has the application been submitted to the Medicines Control Council (MCC) for approval? — N/A
