# Supplementary material for: Femicide, intimate partner femicide, and non-intimate partner femicide in South Africa: An analysis of 3 national surveys, 1999–2017
Source: PLoS Med. 2024 Jan 18;21(1):e1004330. doi: 10.1371/journal.pmed.1004330 (PMC10796052; doi:10.1371/journal.pmed.1004330)
Supplement: S1 Table — (DOCX) [file pmed.1004330.s003.docx]

S1 Table: Count and percentages distribution of all female murder cases in the police system by mortuary strata

|  | **1999*** | | **2009** | | **2017** | |
| --- | --- | --- | --- | --- | --- | --- |
| **Mortuary strata** | All female murder cases identified† | Police data available‡ | All female murder cases identified† | Police data available‡ | All female murder cases identified† | Police data available‡ |
| **Small** | 1393 (36.7%) | 1173 (84.2%) | 770 (32.6%) | 765 (99.4%) | 628 (26.1%) | 567 (90.3%) |
| **Medium** | 836 (22.0%) | 789 (94.4%) | 935 (39.6%) | 918 (98.2%) | 773 (32.1%) | 666 (86.2%) |
| **Large** | 1564 (41.2%) | 1329 (85.0%) | 659 (27.9%) | 642 (97.4%) | 1006 (41.8%) | 795 (76.0%) |
| **Total** | **3793** | **3292 (86.8%)** | **2363** | **2325 (98.4%)** | **2407** | **2028 (84.3%)** |
| *1999: 3292/3296 cases in police system are available by mortuary strata.  † Percentage of murder cases across mortuary size  ‡ Percentage of the female murder cases identified in each mortuary strata | | | | | | |
