# Supplementary material for: Femicide, intimate partner femicide, and non-intimate partner femicide in South Africa: An analysis of 3 national surveys, 1999–2017
Source: PLoS Med. 2024 Jan 18;21(1):e1004330. doi: 10.1371/journal.pmed.1004330 (PMC10796052; doi:10.1371/journal.pmed.1004330)
Supplement: S2 Table — IPF, intimate partner femicide; IRR, incidence rate ratio; NIPF, non-intimate partner femicide. (DOCX) [file pmed.1004330.s004.docx]

S2 Table: Age standardised population rates for 1999, 2009 and 2017 for overall female murders: intimate partner femicide and non-intimate partner femicide by age and race and incidence rate ratios (IRR) of population rate estimates between surveys: weighted and non-imputed data

| **Characteristics** | **1999** | | | **2009** | | | **IRR of Population rate Estimates:**  **2009/1999**  **(95% CI)** | **2017** | | | **IRR of Population Rate Estimates 2017/2009**  **(95% CI)** |
| --- | --- | --- | --- | --- | --- | --- | --- | --- | --- | --- | --- |
|  | **Unweighted= 1.052**  **Weighted= 3.793** | | | **Unweighted= 930**  **Weighted= 2.363** | | |  | **Unweighted=1.301**  **Weighted= 2.407** | | |  |
|  | **N** | **Percent**  **(95% CI)** | **Rate per 100000 pop**  **(95% CI)** | **N** | **Percent**  **(95% CI)** | **Rate per 100000 pop**  **(95% CI)** |  | **N** | **Percent**  **(95% CI)** | **Rate per 100000 pop**  **(95% CI)** |  |
| Overall female murders | 3793 |  | 24.2 (15.5-32.9) | 2363 |  | 12.6(8.5-16.6) | 0.52 (0.48-0.56) | 2407 |  | 11.1 (9.8-12.4) | 0.88 (0.81-0.95) |
| Intimate Partner Femicide | 1349 | 50.3 (44.5-56.0) | 7.8 (4.9-10.7) | 1024 | 57.1(52.8-61.4) | 5.1 (3.3-6.9) | 0.65 (0.58-0.73) | 768 | 56.0 (53.1-58.8) | 3.4 (2.9-3.9) | 0.67 (0.59-0.76) |
| Non-Intimate Partner  Femicide | 1335 | 49.7 (44.0-55.5) | 9.1 (5.3-12.8) | 768 | 42.9 (38.6-47.2) | 4.3 (2.6-5.9) | 0.47 (0.42-0.53) | 604 | 44.0 (41.2-46.9) | 2.8 (2.4-3.3) | 0.65 (0.56-0.76) |
| **Intimate Partner Femicide by age group** |  |  |  |  |  |  |  |  |  |  |  |
| 14-29 y | 649 | 48.1 (39.9-56.4) | 3.2 (2.2-4.3) | 474 | 46.3 (41.7-50.9) | 2.1 (1.4-2.7) | 0.66 (0.56-0.77) | 353 | 46.0 (42.4-49.7) | 1.6 (1.3-1.8) | 0.76 (0.63-0.92) |
| 30-44 y | 524 | 38.8 (30.3-48.1) | 3.2 (2.1-4.4) | 430 | 41.9 (37.4-46.6) | 2.3 (1.5-3.1) | 0.72 (0.60-0.86) | 316 | 41.2 (38.4-44.0) | 1.4 (1.2-1.5) | 0.61 (0.5-0.74**)** |
| 45-59 y | 71 | 5.3 (3.3-8.4) | 0.6 (0.2-1) | 103 | 10.1 (7.5-13.3) | 0.6 (0.4-0.9) | 1.00 (0.66-1.52) | 73 | 9.5 (7.7-11.8) | 0.4 (0.3-0.4) | 0.67 (0.44-1.01) |
| 60+ y | 26 | 1.9 (1.1-3.5) | 0.2 (0.1-0.4) | 14 | 1.3 (0.6-2.8) | 0.1 (0.0-0.2) | 0.50 (0.2-1.23) | 19 | 2.4 (1.8-3.3) | 0.1 (0.1-0.1) | 1.00 (0.38-2.61) |
| Undetermined Age | 79 | 5.8 (2.7-12.0) |  | 4 | 0.4 (0.1-2.1) |  |  | 6 | 0.8 (0.3-1.9) |  |  |
| **Non-Intimate Partner Femicide by age groups** |  |  |  |  |  |  |  |  |  |  |  |
| 14-29 y | 370 | 27.7 (22.6-33.5) | 1.8 (1.1-2.6) | 249 | 32.4 (28.6-36.5) | 1.1 (0.7-1.5) | 0.61 (0.49-0.76) | 225 | 37.2 (34.1-40.4) | 1.0 (0.9-1.1) | 0.91 (0.71-1.17) |
| 30-44 y | 446 | 33.4 (26.4-41.1) | 2.8 (1.9-3.7) | 161 | 21.0 (17.6-24.9) | 0.9 (0.6-1.2) | 0.32 (0.25-0.41) | 159 | 26.4 (22.5-30.6) | 0.7 (0.6-0.8) | 0.78 (0.57-1.05) |
| 45-59 y | 260 | 19.5 (15.1-24.7) | 2.3 (1.4-3.1) | 170 | 22.1 (18.2-26.5) | 1.0 (0.7-1.4) | 0.43 (0.33-0.57) | 105 | 17.5 (14.6-20.7) | 0.5 (0.4-0.6) | 0.50 (0.36-0.70) |
| 60+ y | 223 | 16.7 (10.3-25.8) | 1.9 (0.7-3.1) | 188 | 24.5 (19.4-30.4) | 1.3 (0.8-1.8) | 0.68 (0.52-0.90) | 111 | 18.3 (15.6-21.4) | 0.6 (0.5-0.7) | 0.46 (0.33-0.64) |
| Undetermined Age | 37 |  |  |  |  |  |  | 4 | 0.7 (0.2-2.7) |  |  |
| **Overall Femicide by race group*** |  |  |  |  |  |  |  |  |  |  |  |
| African | 3019 | 80.3 (67.0-89.1) | 25.8 (14.4-37.3) | 1884 | 80.3 (69.1-88.1) | 12.9 (9.2-16.5) | 0.50 (0.46-0.54) | 2078 | 86.4 (83.9-88.5) | 12.3 (11.2-13.5) | 0.96 (0.88-1.05) |
| Colored | 516 | 13.7 (6.3-27.5) | 37.5 (6.7-68.3) | 309 | 13.2 (6.2-25.9) | 17.7 (3.4-32.0) | 0.47 (0.39-0.57) | 233 | 9.7 (7.9-11.9) | 11.9 (9.5-14.4) | 0.67 (0.53-0.85) |
| Indian | 41 | 1.1 (0.4-3.0) | 9.7 (0.0-19.9) | 31 | 1.3 (0.5-3.7) | 5.9 (0.0-12.4) | 0.61 (0.32-1.16) | 21 | 0.9 (0.6-1.4) | 3.6 (2.0-5.2) | 0.60 (0.28-1.30) |
| White | 183 | 4.9 (2.9-8.2) | 9.3 (2.9-15.7) | 116 | 4.9 (2.8-8.5) | 5.5 (2.0-9.1) | 0.60 (0.43-0.82) | 72 | 3.0 (2.4-3.7) | 3.4 (2.6-4.3) | 0.62 (0.42-0.94) |
| **Intimate Partner Femicide by race group** |  |  |  |  |  |  |  |  |  |  |  |
| African | 1023 | 75.8 (60.3-86.7) | 8.8 (5.1-12.4) | 801 | 78.4 (64.7-87.8) | 5.5 (3.8-7.1) | 0.62 (0.55-0.71) | 649 | 84.6 (80.9-87.7) | 3.9 (3.4-4.3) | 0.70 (0.61-0.81) |
| Colored | 252 | 18.7 (8.9-35.0) | 18.3 (2.8-33.9) | 173 | 16.9 (7.8-32.8) | 9.9 (1.7-18.1) | 0.54 (0.41-0.71) | 94 | 12.3 (9.4-16.0) | 4.8 (3.3-6.3) | 0.48 (0.34-0.69) |
| Indian | 21 | 1.5 (0.5-4.9) | 4.9 (0.0-10.5) | 18 | 1.8 (0.7-4.5) | 3.4 (0.0-6.8) | 0.69 (0.29-1.65) | 8 | 1.0 (0.4-2.5) | 1.3 (0.2-2.5) | 0.40 (0.12-1.26) |
| White | 53 | 4.0 (2.3-6.8) | 2.7 (0.7-4.7) | 28 | 2.8 (1.0-7.4) | 1.3 (0.0-2.8) | 0.50 (0.26-0.94) | 16 | 2.1 (1.3-3.4) | 0.8 (0.4-1.2) | 0.58 (0.25-1.35) |
| **Non-Intimate Partner Femicide by age groups** |  |  |  |  |  |  |  |  |  |  |  |
| African | 992 | 75.6 (61.3-85.8) | 8.5 (4.8-12.2) | 582 | 76.7 (62.6-86.7) | 4.0 (2.7-5.3) | 0.47 (0.41-0.54) | 516 | 85.4 (81.5-88.6) | 3.1 (2.7-3.4) | 0.77 (0.65-0.91) |
| Colored | 192 | 14.6 (6.5-29.4) | 13.9 (2.2-25.7) | 115 | 15.2 (6.9-30.0) | 6.6 (1.0-12.2) | 0.47 (0.34-0.65) | 62 | 10.2 (7.9-13.1) | 3.2 (2.4-3.9) | 0.48 (0.31-0.74) |
| Indian | 13 | 1.0 (0.4-2.5) | 3.1 (0.0-6.2) | 8 | 1.0 (0.2-4.4) | 1.5 (0.0-3.7) | 0.49 (0.15-1.68) | 0 |  |  |  |
| White | 116 | 8.9 (4.9-15.5) | 5.9 (1.4-10.4) | 52 | 6.8 (3.5-12.8) | 2.5 (0.8-4.2) | 0.42 (0.47-0.66) | 27 | 4.4 (3.1-6.1) | 1.3 (0.9-1.7) | 0.52 (0.27-1.00) |
| **All analysis on age standardized rates was calculated using the Thembisa model except for race group that used Statistics South Africa mid population estimates**  **1999 female population**: Overall: 15,775,803; By Age: 14-29 years: 6,976,810; 30-44 years: 4,564,389; 45-59 years: 2,427,045 & 60 + years: 1,807,559  **2009 female population**: Overall: 18,982,433; By Age: 14-29 years: 7,960,305; 30-44 years: 5,248,792; 45-59 years: 3,460,820 & 60 + years: 2,312,516  **2017 female population**: Overall: 21,520,499; By Age: 14-29 years: 7,872,159; 30-44 years: 6,459,190; 45-59 years: 4,195,543 & 60 + years: 2,993,607  For race groups rates: the Statistics South Africa mid population estimates were used as race population was not available for race from the Thembisa model.  1999 female population: Overall: 15,458,162; By race: African: 11,683,651; Colored: 1,375,413; Indian: 424,331 & White: 1,984,767  2009 female population: Overall: 19,027,717; By race: African: 14,655,388; Colored: 1,744,521; Indian: 529,557 & White: 2,098,251  2017 female population: Overall: 21,501,234; By race: African: 16,862,856; Colored: 1,958,404; Indian: 595,297 & White: 2,084,677  Age standardized rates were calculated to accounted for undetermined age by multiplying the age standardized rate not accounting for undetermined age with a factor calculated by taking total number of female homicide cases divided by the total number of female homicide cases minus the total number of female homicide cases with undetermined age.  Incidence rate ratio (IRR) is calculated by taking the fractions of the exposed and their total population. The 95% CI (Confidence Intervals) are then adjusted from normal approximation calculation to account for the design effect of the survey. | | | | | | | | | | | |
