# Supplementary material for: Femicide, intimate partner femicide, and non-intimate partner femicide in South Africa: An analysis of 3 national surveys, 1999–2017
Source: PLoS Med. 2024 Jan 18;21(1):e1004330. doi: 10.1371/journal.pmed.1004330 (PMC10796052; doi:10.1371/journal.pmed.1004330)
Supplement: S3 Table — IPF, intimate partner femicide; NIPF, non-intimate partner femicide. (DOCX) [file pmed.1004330.s005.docx]

Table S3: Age standardised population rates for 1999, 2009 and 2017 for all female murders· intimate partner femicide and non-intimate partner femicide by death mechanism and incidence rate ratios (IRR) of population rate estimates between surveys: weighted and non-imputed data

| **Characteristics** | **1999** | | | **2009** | | | **IRR of Population rate Estimates:**  **2009/1999**  **(95% CI)** | **2017** | | | **IRR of Population Rate Estimates 2017/2009**  **(95% CI)** |
| --- | --- | --- | --- | --- | --- | --- | --- | --- | --- | --- | --- |
|  | **Unweighted= 1052**  **Weighted= 3793**  **Intimate Partner Femicide Weighted=1349**  **Non-Intimate Partner Femicide Weighted=1335** | | | **Overall Unweighted= 930**  **Overall Weighted= 2363**  **Intimate Partner Femicide Weighted=1024**  **Non-Intimate Partner Femicide Weighted=768** | | |  | **Overall Unweighted=1301**  **Overall Weighted= 2407**  **Intimate Partner Femicide Weighted=768**  **Non-Intimate Partner Femicide Weighted=604** | | |  |
|  | **N** | **Percent**  **(95% CI)** | **Rate per 100000 pop**  **(95% CI)** | **N** | **Percent**  **(95% CI)** | **Rate per 100000 pop**  **(95% CI)** |  | **N** | **Percent**  **(95% CI)** | **Rate per 100000 pop**  **(95% CI)** |  |
| **Firearm deaths** |  |  |  |  |  |  |  |  |  |  |  |
| All female murders | ^¥^1147 | 33·4 (24.9-43.1) | 7.2 (2.7-11.7) | 462 | 19.5 (15.1-25.0) | 2.4 (1.2-3.6) | 0.33 (0.29-0.39) | 563 | 23.4 (22.2-24.7) | 2.6 (2.1-3.1) | 1.08 (0.91-1.29) |
| Intimate Partner Femicide | **†**405 | 30.6 (21.1-42.1) | 2.2 (0.9-3.6) | 179 | 17.4 (12.1-24.6) | 0.9 (0.4-1.4) | 0.41 (0.32-0.52) | 90 | 11.8 (9.6-14.3) | 0.4 (0.3-0.6) | 0.44 (0.31-0.63) |
| Non-Intimate Partner Femicide | **‡**434 | 33.7 (24.0-44.9) | 2.9 (0.8-5) | 132 | 17.2 (11.6-24.7) | 0.7 (0.2-1.2) | 0.24 (0.18-0.32) | 106 | 17.6 (15.4-20.0) | 0.5 (0.3-0.7) | 0.71 (0.5-1.02) |
| **Stab injury deaths** |  |  |  |  |  |  |  |  |  |  |  |
| All female murders | ^¥^1049 | 30.5 (24.0-37.9) | 6.6 (3.1-10.0) | 668 | 28.3 (23.6-33.5) | 3.5 (2.0-5.0) | 0.53 (0.46-0.61) | ^¥¥^897 | 37.6 (35.8-39.4) | 4.1 (3.4-4.8) | **1.17 (1.02-1.35)** |
| Intimate Partner Femicide | **†**440 | 33.3 (26.3-41.1) | 2.6 (1-4.3) | 322 | 31.5 (25.4-38.2) | 1.6 (0.9-2.4) | 0.62 (0.50-0.75) | **¥**345 | 45.0 (41.2-48.9) | 1.6 (1.2-1.9) | 1.00 (0.81-1.23) |
| Non-Intimate Partner Femicide | **‡**444 | 34.4 (24.4-46.0) | 2.9 (1-4.7) | 273 | 35.5 (29.2-42.4) | 1.5 (0.7-2.3) | 0.52 (0.42-0.64) | 257 | 42.6 (38.1-47.3) | 1.2 (0.9-1.5) | 0.80 (0.63-1.01) |
| **Blunt force injury deaths** |  |  |  |  |  |  |  |  |  |  |  |
| All female murders | 943 | 24.9 (17.7-33.8) | 6.0 (2.5-9.6) | 580 | 24.5 (20.5-29.0) | 3.1 (1.7-4.5) | 0.52(0.45-0.60) | 501 | 20.8 (19.3-22.3) | 2.3 (1.9-2.8) | 0.74 (0.63-0.88) |
| Intimate Partner Femicide | 449 | 33.3 (24.9-42.9) | 2.6 (1-4.3) | 303 | 29.6 (24.0-35.8) | 1.5 (0.8-2.3) | 0.58 (0.47-0.71) | 204 | 26.5 (23.8-29.5) | 0.9 (0.7-1.1) | 0.60 (0.47-0.77) |
| Non-Intimate Partner Femicide | 283 | 21.2 (15.0-29.2) | 2.0 (0.5-3.6) | 173 | 22.5 (16.9-29.3) | 1.0 (0.4-1.7) | 0.50 (0.38-0.65) | 117 | 19.3 (15.9-23.3) | 0.6 (0.3-0.8) | 0.60 (0.43-0.83) |
| **1999 female population**: Overall: 15,775,803  **2009 female population**: Overall: 18,982,433  **2017 female population**: Overall: 21,520,499  ^¥^N=3439; [^¥¥^N=2387](file:///\\N=2387)^;^ **†** N=1322; **‡** N=1290; **¥** N=766 CI=Confidence Interval | | | | | | | | | | | |
